# Incidence and risk factors for malignancy in patients with incidental solitary pulmonary nodules: a systematic review and meta-analysis

**DOI:** 10.1080/07853890.2025.2596547

**Published:** 2026-02-05

**Authors:** Huiyu Zheng, Zhipeng Shao, Wensong Shi, He Qian, Yuchen Zhang

**Affiliations:** Thoracic Surgery Department/Zhengzhou People’s Hospital/Zhengzhou, China

**Keywords:** Incidental solitary pulmonary nodules, malignancy, incidence, risk factors, meta-analysis

## Abstract

**Background:**

The increasing use of chest imaging has led to a higher detection rate of incidental solitary pulmonary nodules (SPNs), often causing patient anxiety. Determining the malignancy rate and associated risk factors is crucial for developing appropriate follow-up strategies to prevent overdiagnosis, overtreatment, or missed diagnoses. This meta-analysis aims to investigate the malignancy rate and risk factors in patients with incidental SPNs.

**Methods:**

A systematic search of PubMed, Embase, Web of Science, and the Cochrane Library was conducted up to June 30, 2025. Data on malignancy rates and potential risk factors were extracted from eligible studies. All pooled analyses were performed using a random-effects model.

**Results:**

Fifty-four studies involving 19,985 patients were included. The pooled malignancy rate for incidental SPNs was 56.7% (95% CI: 51.5–62.0), with significant between-study heterogeneity (*I*^2^ = 98.5%, *p* < 0.001). The pooled effect size showed a minimal change after adjustment for potential publication bias using the non-parametric Trim-and-Fill method (54.7%; 95%CI: 50.9–58.8). Risk factor analysis identified that older age, history of cancer, cigarette smoker, larger nodule diameter, spiculation, upper lobe location, lobulation, pleural indentation, vascular convergence, solid nodules, family history of cancer, and irregular or ill-defined margins were significantly associated with an increased risk of malignancy. Conversely, male sex, presence of calcification, and clear borders were significantly associated with a reduced risk of malignancy.

**Conclusion:**

This meta-analysis provides a comprehensive assessment of malignancy rates and risk factors in incidental SPNs. The high pooled malignancy rate should be interpreted considering the significant heterogeneity and the inclusion of a high proportion of retrospective studies and populations from high-risk regions. Nonetheless, these findings offer essential evidence for clinical risk stratification, supporting optimized follow-up and informed decision-making.

## 1. Introduction

Globally, lung cancer emerged as the leading malignancy in 2022, accounting for approximately 2.5 million new cases [1]. Studies reveal a dramatic disparity in outcomes: the 5-year survival rate ranges from 60% to 80% for stage I disease but drops sharply to just 12% for stage III C, underscoring the crucial importance of early diagnosis for patient prognosis [2,3]. With the widespread adoption of chest imaging technologies such as multi-detector computed tomography, the detection rate of incidental solitary pulmonary nodules (SPNs) has increased considerably in recent years [4]. SPNs are defined as incidentally discovered, well-circumscribed, round lesions measuring ≤ 3 cm in diameter on chest imaging, without associated abnormalities such as atelectasis, pneumonia, or lymphadenopathy [5].

This raising detection rate presents a dual clinical challenge. On one hand, some SPNs represent early-stage lung cancer, where delayed diagnosis can compromise treatment efficacy. On the other hand, most SPNs are benign; unnecessary testing or interventions may subject patients to physical and psychological burdens and increase healthcare costs [6]. Therefore, accurately assessing the malignancy risk of SPNs and identifying relevant risk factors is essential for clinical management.

Previous studies have explored various factors associated with SPN malignancy risk, including nodule characteristics (e.g. size, shape, density) and patient clinical features [7]. However, findings across studies often vary due to differences in study populations, sample sizes, and methodologies. For example, the malignancy risk threshold related to nodule size varies considerably, and the influence of smoking history may differ across populations [8]. This heterogeneity persists despite the development of several major clinical practice guidelines for managing incidental pulmonary nodules, such as those from the Fleischner Society, the British Thoracic Society (BTS), and the American College of Chest Physicians (ACCP) [9–11]. While these guidelines provide invaluable frameworks, they also reflect underlying uncertainties and the continuous evolution of evidence, particularly concerning the precise quantification of risk associated with specific factors in diverse populations. This ongoing challenge highlights the need for a synthesized, high-level evidence base.

In this context, a systematic review and meta-analysis of existing research can help overcome the limitations of individual studies by providing a more reliable estimate of SPN malignancy incidence and clarifying the true associations between risk factors and malignancy. This study aims to assess the overall malignancy rate in patients with incidental SPNs and to conduct an in-depth analysis of potential risk factors. The results will provide high-quality, evidence-based guidance to assist clinicians in risk stratification and the development of personalized follow-up and management strategies.

## 2. Methods

### 2.1. Data sources, search strategy, and selection criteria

This systematic review was conducted in full compliance with the PRISMA (Preferred Reporting Items for Systematic Reviews and Meta-Analyses) guidelines to ensure methodological rigor and transparency [12]. The study protocol was registered on the INPLASY platform (registration number: INPLASY202570077). Our objective was to comprehensively assess the incidence of malignancy and identify related risk factors in patients with incidental SPNs. We performed an extensive search for relevant epidemiological studies without restrictions on language or publication status, including published articles, unpublished data, and conference abstracts.

Four major databases—PubMed, Embase, Web of Science, and the Cochrane Library—were searched using core keywords and their variants, such as ‘SPNs,’ ‘malignancy,’ and ‘lung cancer.’ The search was updated through June 30, 2025, to include the most recent data. The complete search strategies for each database, including specific keyword combinations and filters, are provided in Supplementary File 1. To ensure comprehensive coverage, we also manually screened the reference lists of all identified primary studies and relevant review articles to identify any additional studies that might have been missed in the database searches.

Two reviewers independently conducted the literature screening and study selection using a standardized process. Discrepancies were resolved through discussions until consensus was reached. Studies were included if they met the following criteria: (1) Participants: patients with incidental SPNs (defined as rounded lesions ≤3 cm in diameter, detected *via* imaging without prior suspicion of malignancy); (2) Exposure: incidental SPNs confirmed as malignant or pathologically diagnosed as lung cancer; (3) Comparison: incidental SPNs confirmed as benign; (4) Outcomes: reported incidence of malignancy in incidental SPNs and associated risk factors. For a risk factor to be included in the meta-analysis, it had to be reported in at least three independent studies to ensure the stability and reliability of pooled estimates. The criteria for verifying benign and malignant nodules were pre-specified: malignancy required histopathological confirmation (*via* surgery or biopsy), while benign status was defined by either definitive histopathology; resolution or significant regression on follow-up imaging over at least 24 months; or characteristic benign calcification patterns confirmed by an experienced radiologist. Studies that did not apply comparable rigorous verification standards were excluded; (5) Study design: observational studies. Exclusion criteria were: (1) non-incidental SPNs; (2) insufficient confirmation of diagnosis; (3) incomplete data; and (4) significant imaging limitations.

To identify and eliminate duplicate publications or overlapping patient cohorts, we implemented a rigorous deduplication process after merging search results from all databases. For studies from the same institution or research group, we compared authors, patient recruitment periods, study center locations, sample sizes, and baseline cohort characteristics. In cases of suspected overlap, the study with the larger sample size, more comprehensive reporting, or longer follow-up was prioritized. If uncertainty remained, corresponding authors were contacted for clarification. No overlapping cohorts were identified in the final set of included studies.

### 2.2. Data collection and quality assessment

Two reviewers independently performed the following tasks: (1) Data extraction, collecting information on first author, publication year, study design, country, sample size, mean participant age, sex distribution (proportion of males), smoking rate, number of benign and malignant SPNs, and diagnostic methods; (2) Quality assessment using the Newcastle-Ottawa Scale (NOS), which evaluates studies based on selection, comparability, and outcome assessment [13]. All extracted data and quality assessment results were cross-checked. Disagreements were resolved by a third reviewer through verification of the original literature and discussion, ensuring data accuracy and consistency in quality assessment.

### 2.3. Statistical analysis

We performed a random-effects meta-analysis to estimate the pooled incidence of malignancy in incidental SPNs. To enhance data comparability, all raw data were log-transformed prior to analysis [14]. Restricted maximum likelihood estimation was applied to improve the accuracy of parameter estimates. Effect sizes for factors associated with malignancy were expressed as odds ratios (ORs) with 95% confidence intervals (CIs), pooled using the random-effects model [14].

Between-study heterogeneity was assessed using the *I^2^* statistic and Q-test, with *I^2^* ≥ 50% or a Q-test *P*-value < 0.10 indicating significant heterogeneity [15,16]. Sensitivity analyses were performed by sequentially excluding individual studies to evaluate the robustness of the results [17]. Subgroup analyses were conducted based on publication year, study design, geographic region, and study quality to explore potential sources of heterogeneity. Differences between subgroups were compared using interaction t-tests, assuming a normal distribution of the analytical data [18]. For sources of heterogeneity that could not be quantitatively assessed *via* meta-regression due to insufficient primary data, a qualitative assessment was performed by examining the methodologies of the included studies. Publication bias was assessed using both qualitative (funnel plot) and quantitative (Egger’s and Begg’s tests) methods [19,20]. All statistical tests were two-sided, with a significance threshold of *p* < 0.05. Data analysis was performed using STATA 18.0 (StataCorp, College Station, TX, USA).

## 3. Results

### 3.1. Literature search

**Figure 1.**
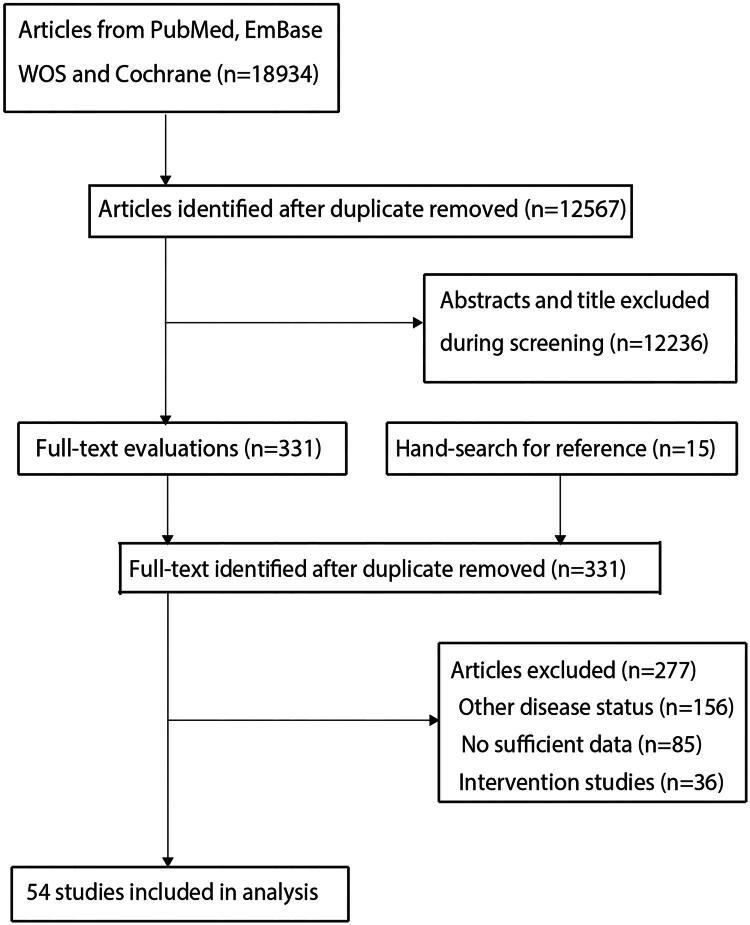
The PRISMA flowchart regarding the literature search and study selection.

Our systematic search identified 18,934 records from electronic databases. After removing duplicates, 12,567 unique studies underwent title/abstract screening, which led to the exclusion of 12,236 irrelevant publications. A full-text review of the remaining 331 potentially eligible studies resulted in the exclusion of 277 that did not meet the inclusion criteria. The final meta-analysis included 54 studies [21–74], with no additional eligible studies identified through manual screening of reference lists. The complete study selection process is illustrated in Figure 1.

### 3.2. Study characteristics

The baseline characteristics of the 54 included studies, encompassing 19,985 patients, are summarized in Table 1. Sample sizes ranged from 107 to 1,679 participants. The majority of studies were retrospective cohort designs (*n* = 52), while two were prospective cohort studies. Geographically, 45 studies were conducted in China, with the remaining nine originating from seven other countries: USA, Switzerland, Spain, Korea, Turkey, Portugal, and Greece. Quality assessment using the NOS yielded scores of 8 points (*n* = 5), 7 points (*n* = 29), and 6 points (*n* = 20) points.

**Table 1.** The baseline characteristics of identified studies and involved patients.

Study	Study design	Country	Sample size	Age (years)	Male (%)	Smoking (%)	Benign	Malignancy	Verification standards	NOS
Swensen 1997 [21]	Retrospective	USA	552	61.3	51.4	67.8	406	146	Histopathology	7
Gould 2007 [22]	Prospective	USA	375	65.9	97.9	94.1	171	204	Imaging follow-up	8
Yang 2010 [23]	Retrospective	China	390	57.1	54.4	39.2	130	260	Pathology	6
Li 2012 [24]	Retrospective	China	371	56.1	53.1	42.0	142	229	Pathology	6
Dong 2014 [25]	Retrospective	China	1679	57.9	58.9	42.5	1296	383	Histopathology	7
Shi 2014 [26]	Retrospective	China	341	59.8	55.7	NA	100	241	Imaging follow-up	6
Gómez-Sáez 2015 [27]	Prospective	Spain	413	NA	63.2	61.0	362	51	Histopathology	8
Zhang 2015 [28]	Retrospective	China	294	56.6	52.0	41.5	118	176	Imaging follow-up	7
Zheng 2015 [29]	Retrospective	China	846	54.8	54.7	25.9	533	313	Imaging follow-up	6
Choi 2016 [30]	Retrospective	Korea	107	60.0	57.0	36.4	31	76	Histopathology	6
Hanauer 2016 [31]	Retrospective	Switzerland	181	63.0	50.3	61.3	74	107	Histopathology	6
Hu 2016 [32]	Retrospective	China	112	54.7	32.1	NA	30	82	Pathology	6
Yu 2016 [33]	Retrospective	China	317	57.0	50.8	27.8	155	162	Pathology	7
Li 2016 [34]	Retrospective	China	200	61.0	52.4	NA	122	78	Histopathology	6
Xiang 2016 [35]	Retrospective	China	203	59.9	54.2	46.3	44	159	Pathology	7
Bellier 2017 [36]	Retrospective	Switzerland	140	65.0	54.0	54.0	34	106	Histopathology	6
Ma 2017 [37]	Retrospective	China	137	66.7	66.4	59.1	69	68	Histopathology	6
She 2017 [38]	Retrospective	China	899	58.9	55.8	20.2	294	605	Pathology	7
She 2017 [39]	Retrospective	China	449	54.2	36.5	11.4	242	207	Pathology	7
Yang 2017 [40]	Retrospective	China	1078	55.4	60.0	38.4	182	721	Histopathology	7
Wang 2018 [41]	Retrospective	China	250	52.5	25.2	NA	85	165	Histopathology	8
Wang 2018 [42]	Retrospective	China	268	58.4	52.6	34.3	112	156	Pathology	7
Yang 2018 [43]	Retrospective	China	198	54.0	51.0	NA	66	132	Pathology	7
Chen 2019 [44]	Retrospective	China	493	52.3	49.1	33.7	279	214	Histopathology	7
Xiao 2019 [45]	Retrospective	China	362	55.2	48.3	27.6	49	313	Pathology	7
Chen 2020 [46]	Retrospective	China	216	51.2	26.4	8.3	56	160	Histopathology	7
Chen 2020 [47]	Retrospective	China	150	55.4	55.3	NA	73	77	Histopathology	7
Feng 2020 [48]	Retrospective	China	218	56.6	55.0	NA	92	126	Pathology	6
Zhang 2020 [49]	Retrospective	China	1412	55.0	46.0	31.3	706	706	Histopathology	8
Feng 2020 [50]	Retrospective	China	123	56.1	56.1	NA	58	65	Pathology	7
Guo 2020 [51]	Retrospective	China	312	60.1	55.1	26.0	97	215	Pathology	7
Erdogu 2021 [52]	Retrospective	Turkey	180	58.1	63.9	67.8	63	117	Histopathology	7
Hou 2021 [53]	Retrospective	China	238	60.0	47.5	29.4	113	125	Imaging follow-up	7
Wu 2021 [54]	Retrospective	China	995	57.9	59.2	47.1	223	772	Pathology	8
Zhao 2021 [55]	Retrospective	China	250	60.9	56.4	44.0	94	156	Pathology	7
Lin 2021 [56]	Retrospective	China	348	55.5	46.8	NA	171	177	Pathology	7
Tang 2021 [57]	Retrospective	China	141	59.8	48.9	31.9	81	60	Pathology	7
Zhuo 2021 [58]	Retrospective	China	313	49.8	65.5	NA	217	96	Pathology	6
Jacob 2022 [59]	Retrospective	Portugal	121	64.7	62.0	63.6	57	64	Histopathology	7
Yi 2022 [60]	Retrospective	China	200	59.0	62.0	NA	95	105	Pathology	6
He 2022 [61]	Retrospective	China	151	56.7	50.3	NA	73	78	Pathology	6
Chen 2023 [62]	Retrospective	China	147	56.6	40.8	18.4	82	65	Pathology	6
Xie 2023 [63]	Retrospective	China	132	63.3	43.2	55.3	30	102	Pathology	7
Jiang 2023 [64]	Retrospective	China	295	61.9	66.1	51.2	125	170	Pathology	6
Zhang 2023 [65]	Retrospective	China	289	60.0	52.9	33.9	142	147	Histopathology	7
Li 2023 [66]	Retrospective	China	112	NA	59.8	48.2	43	69	Histopathology	6
Liu 2024 [67]	Retrospective	China	226	56.0	37.6	19.0	116	110	Pathology	7
He 2024 [68]	Retrospective	China	794	56.4	52.6	38.0	473	321	Imaging follow-up	7
Qu 2024 [69]	Retrospective	China	360	59.9	60.8	NA	147	213	Pathology	7
Wang 2024 [70]	Retrospective	China	187	57.9	0.0	NA	73	114	Pathology	6
Zheng 2024 [71]	Retrospective	China	190	59.4	50.5	NA	69	121	Histopathology	7
Apostolopoulos 2024 [72]	Retrospective	Greece	456	66.0	69.0	NA	222	234	Histopathology	7
Chen 2025 [73]	Retrospective	China	441	56.9	58.0	NA	105	336	Pathology	6
Zhao 2025 [74]	Retrospective	China	333	55.9	55.9	NA	105	228	Pathology	6

### 3.3. Incidence of malignancy in patients with incidental SPNs

**Figure 2.**
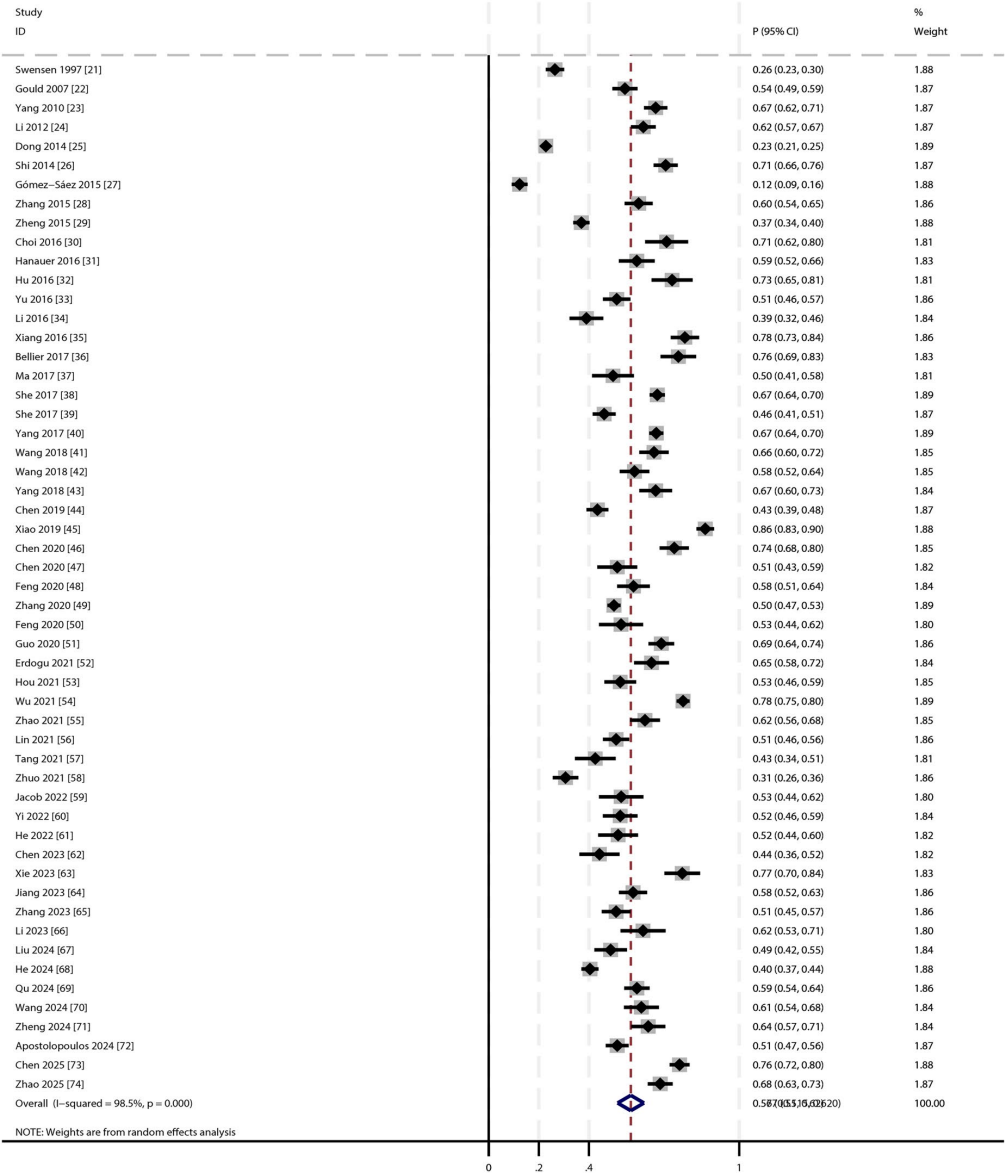
The forest plot for the incidence of malignancy in patients with incidental solitary pulmonary nodules.

**Figure 3.**
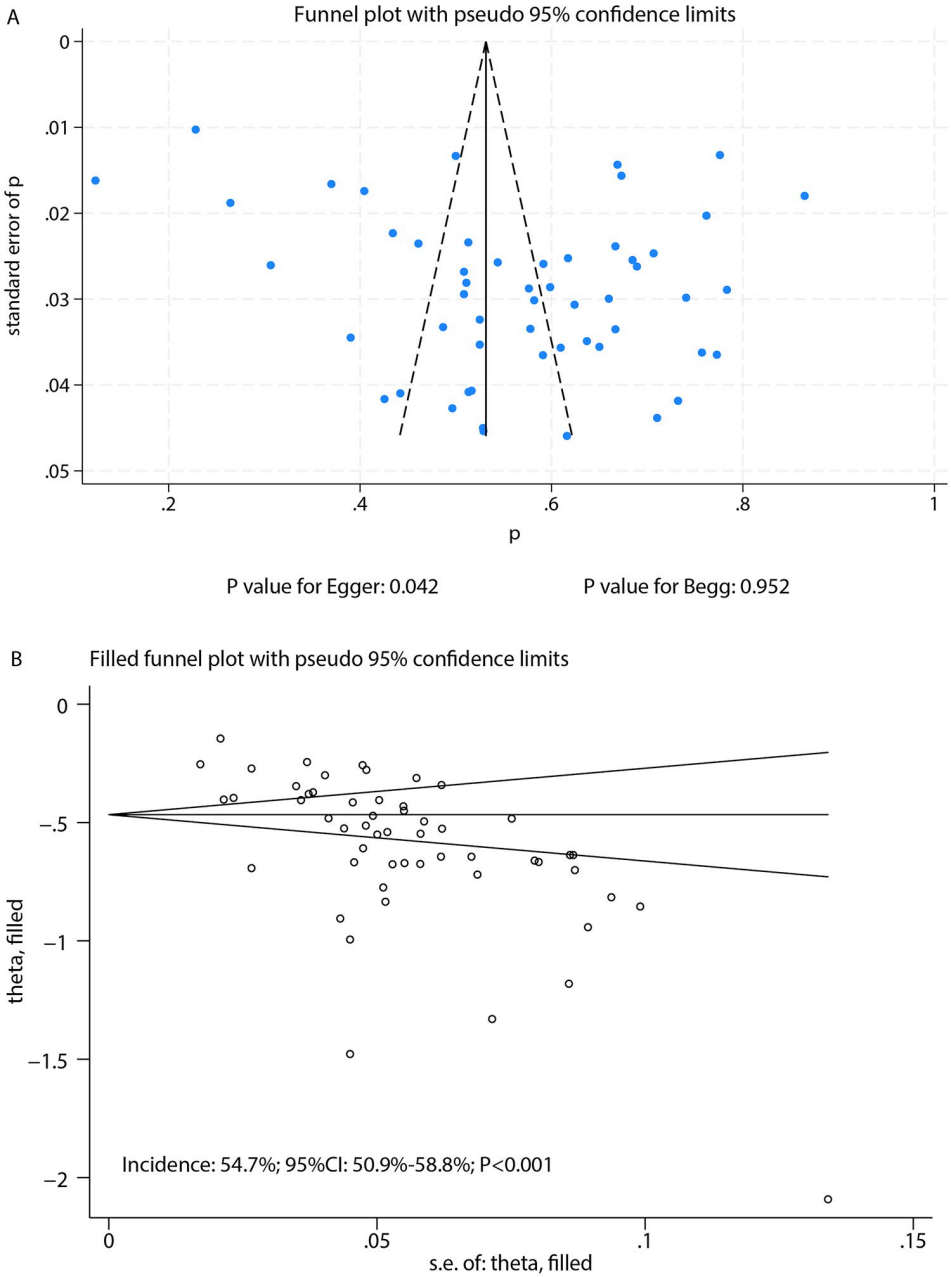
Funnel plot for the assessment of publication bias in the meta-analysis of malignancy incidence. A: Funnel plot of the 54 included studies in the primary analysis; B: Funnel plot following the Trim-and-Fill adjustment. The minimal shift from the original estimate (56.7%) to the adjusted estimate (54.7%) indicates robustness of the finding.

The pooled malignancy rate for incidental SPNs was 56.7% (95% CI: 51.5–62.0; Figure 2). Given the substantial heterogeneity (*I^2^* = 98.5%, *p* < 0.001), the 95% prediction interval, which estimates the range within which the true incidence in a new, similar study would fall, was wide, ranging from 31.8% to 78.9%. Sensitivity analysis demonstrated stable estimates, with malignancy rates ranging from 56.1% to 57.4% upon sequential exclusion of individual studies (Supplementary Figure S1). To directly address the geographical distribution of our sample, a subgroup analysis was performed comparing studies from China with studies from other countries. The pooled malignancy incidence was significantly higher in the Chinese studies (57.7% [95% CI: 52.4–63.0]) compared to the non-Chinese studies (51.9% [95% CI: 36.2–67.5]) (*P* for subgroup difference < 0.001). Additional subgroup analyses revealed the following trends: (1) studies published after 2020 reported slightly higher malignancy risks; (2) retrospective cohort studies showed significantly higher malignancy rates than prospective cohorts; (3) the highest malignancy risk was observed in studies from Korea, while the lowest was reported in studies from Spain; and (4) studies with lower NOS quality scores tended to report higher malignancy risks compared to those with high scores (Supplementary Figures S2–S5). A qualitative assessment of methodological variations across the included studies identified further potential sources of heterogeneity. Although the core definition of an SPN was consistently applied, operational details often differed, including variations in imaging protocols, generations of CT scanners used, and methods for measuring nodule diameter. These inconsistencies in diagnostic criteria and imaging techniques, which were not uniformly reported and thus could not be included in a quantitative analysis, likely contributed substantially to the observed heterogeneity. Publication bias assessment showed a non-significant Begg’s test (*p* = 0.952) but a significant Egger’s test (*p* = 0.042), suggesting potential small-study effects (Figure 3(A)). We applied the non-parametric Trim-and-Fill method to adjust for potential publication bias. The adjusted pooled malignancy rate was 54.7% (95% CI: 50.9–58.8), which represents a minimal change from the original estimate of 56.7% (Figure 3(B)).

### 3.4. Risk factors for malignancy in patients with incidental SPNs

We systematically analysed factors associated with malignancy risk in incidental SPNs (Table 2 and Supplementary Figures S6–S20). Several factors were significantly associated with an increased risk of malignancyk: older age (OR: 1.04; 95% CI: 1.03–1.06; *p* < 0.001), history of cancer (OR: 3.42; 95% CI: 1.61–7.24; *p* = 0.001), cigarette smoker (OR: 2.12; 95% CI: 1.53–2.95; *p* < 0.001), larger nodule diameter (OR: 1.19; 95% CI: 1.13–1.25; *p* < 0.001), spiculation (OR: 3.67; 95% CI: 2.86–4.72; *p* < 0.001), upper lobe location (OR: 1.78; 95% CI: 1.25–2.54; *p* = 0.001), lobulation (OR: 3.84; 95% CI: 2.79–5.29; *p* < 0.001), pleural indentation (OR: 2.84; 95% CI: 2.24–3.60; *p* < 0.001), vascular convergence (OR: 4.32; 95% CI: 3.03–6.16; *p* < 0.001), solid nodule appearance (OR: 4.58; 95% CI: 2.87–7.30; *p* < 0.001), family history of cancer (OR: 3.59; 95% CI: 1.90–6.78; *p* < 0.001), and irregular or ill-defined margins (OR: 2.22; 95% CI: 1.28–3.83; *p* = 0.004). Conversely, male sex (OR: 0.58; 95% CI: 0.38–0.89; *p* = 0.012), presence of calcification (OR: 0.21; 95% CI: 0.10–0.46; *p* < 0.001), and clear borders (OR: 0.19; 95% CI: 0.12–0.29; *p* < 0.001) were significantly associated with a reduced risk of malignancy. Air bronchogram, and satellite lesions showed no significant association with malignancy risk.

**Table 2.** The summary results for the risk factors of malignancy in patients with incidental SPNs.

Factors	No of studies	OR and 95%CI	*P* value	I^2^ (%)	Q statistic
Age	21	1.04 (1.03–1.06)	<0.001	81.2	<0.001
Male vs female	13	0.58 (0.38–0.89)	0.012	76.3	<0.001
History of cancer	6	3.42 (1.61–7.24)	0.001	87.0	<0.001
Cigarette smoker	14	2.12 (1.53–2.95)	<0.001	80.0	<0.001
Diameter of nodules	17	1.19 (1.13–1.25)	<0.001	88.4	<0.001
Spiculation	22	3.67 (2.86–4.72)	<0.001	77.7	<0.001
Upper lobe	8	1.78 (1.25–2.54)	0.001	84.9	<0.001
Presence of calcification	10	0.21 (0.10–0.46)	<0.001	85.8	<0.001
Lobulation	15	3.84 (2.79–5.29)	<0.001	67.4	<0.001
Pleural indentation	13	2.84 (2.24–3.60)	<0.001	17.3	0.270
Clear border	10	0.19 (0.12–0.29)	<0.001	57.8	0.011
Vascular convergence	8	4.32 (3.03–6.16)	<0.001	32.3	0.170
Solid nodules*	5	4.58 (2.87–7.30)	<0.001	0.0	0.423
Air bronchogram	6	2.48 (0.90–6.80)	0.078	83.0	<0.001
Family history of cancer	4	3.59 (1.90–6.78)	<0.001	33.4	0.212
Irregular or ill-defined margins	3	2.22 (1.28–3.83)	0.004	54.9	0.109
Satellite lesions	3	0.27 (0.07–1.09)	0.066	75.8	0.016

* a ‘solid nodule’ was defined as a nodule that completely obscures the underlying lung parenchyma. This was contrasted with ‘part-solid’ (containing both ground-glass and solid components) and ‘pure ground-glass’ nodules, collectively referred to as ‘subsolid’ nodules. It is noted that this classification can be influenced by CT slice thickness, and the included studies primarily utilized modern multi-detector CT with slice thickness ≤1.5 mm.

Significant heterogeneity was observed for several factors, including age (*I^2^* = 81.2%, *p* < 0.001), sex (*I^2^* = 76.3%, *p* < 0.001), history of cancer (*I^2^* = 87.0%, *p* < 0.001), cigarette smoker (*I^2^* = 80.0%, *p* < 0.001), nodule diameter (*I^2^* = 88.4%, *p* < 0.001), spiculation (*I^2^* = 77.7%, *p* < 0.001), upper lobe location (*I^2^* = 84.9%, *p* < 0.001), calcification (*I^2^* = 85.8%, *p* < 0.001), lobulation (*I^2^* = 67.4%, *p* < 0.001), clear border (*I^2^* = 57.8%, *p* = 0.011), air bronchogram (*I^2^* = 83.0%, *p* < 0.001), and satellite lesions (*I^2^* = 75.8%, *p* = 0.016).

Sensitivity analysis indicated that the association for air bronchogram became statistically significant after excluding the study by Wang et al. [41]. For all other identified risk factors, the associations remained robust even in sensitivity analyses (Supplementary Figures S21-S34).

Publication bias assessment for individual risk factors is presented in Supplementary Figures S35–S48. The results indicated potential publication bias for history of cancer, cigarette smoker, nodule diameter, spiculation, upper lobe location, and vascular convergence. However, after adjusting for potential publication bias, the overall conclusions for these factors remained unchanged.

## 4. Discussion

This study, comprising 54 studies with a total of 19,985 patients, represents the first large-scale comprehensive assessment of malignancy incidence and associated risk factors in incidental SPNs. The pooled analysis showed an overall malignancy rate of 56.7%, with several patient characteristics and radiographic features significantly associated with malignancy risk.

Our pooled malignancy rate of 56.7% is notably higher than rates often cited in older literature. While advancements in CT imaging resolution may contribute to the detection of earlier-stage malignancies [75], clinical setting and population-specific risk factors appear to be more influential drivers of this elevated estimate. The observed gradient from screening cohorts (lowest risk) to surgical series (highest risk) demonstrates that clinical context is a paramount determinant of pre-test probability. Furthermore, the significant difference in malignancy incidence between Chinese and non-Chinese studies, coupled with our finding that study-level characteristics significantly predict malignancy rates, strongly implicates geographical and epidemiological factors as primary contributors.

We observed substantial heterogeneity across studies, attributable to several factors. Our subgroup analysis confirmed significantly higher malignancy incidence in Chinese populations compared to non-Chinese populations, underscoring the profound impact of regional factors on SPN malignancy risk. The elevated rate in Chinese studies may reflect a combination of high prevalence of risk factors such as smoking and environmental exposures, alongside genetic predispositions to certain lung cancer subtypes more common in Asian populations. This reinforces that our pooled global estimate should be applied cautiously, with clinicians prioritizing local or regional data when available for pre-test probability assessment. Retrospective cohort studies reported significantly higher malignancy rates compared to prospective studies, likely because retrospective designs typically rely on clinical databases that disproportionately include patients with more suspicious nodule characteristics. These patients are more likely to undergo intensive evaluation, potentially inflating malignancy estimates. In contrast, prospective studies generally encompass a broader spectrum of nodules, including lower-risk cases that may not require invasive evaluation, thus providing estimates more representative of the general population. Additionally, studies with lower methodological quality reported higher malignancy rates, possibly reflecting non-standardized data collection methods and inconsistent diagnostic criteria. Finally, clinical implications of this substantial heterogeneity are significant. The pooled malignancy rate of 56.7% should not be interpreted as a universal probability for every patient with an SPN. Instead, our findings emphasize that pre-test probability of malignancy is highly context-dependent. Therefore, the primary clinical value of this meta-analysis lies not in the aggregate incidence rate, but in the consistent direction and magnitude of associations we identified for specific risk factors. For instance, despite heterogeneity, the presence of spiculation or larger nodule size consistently and strongly predicted malignancy across diverse settings, reinforcing these imaging features as robust biomarkers of risk. Consequently, clinicians should utilize our results as a validated risk stratification framework rather than a precise calculator. The identified risk and protective factors provide a powerful tool to guide shared decision-making, helping triage patients toward appropriate diagnostic pathways or active surveillance, while integrating these factors with patient-specific context and local expertise.

Patient-related factors significantly associated with increased malignancy risk included older age, cigarette smoker, personal cancer history, and family history of cancer. Conversely, male sex appeared to have a protective effect. The underlying mechanisms for these associations may be explained as follows: First, each additional year of age was associated with a 4% increase in malignancy risk, likely due to age-related declines in DNA repair capacity, cumulative genetic mutations, prolonged carcinogen exposure, and weakened immune surveillance in older individuals [76]. Second, smokers had a 2.12-fold higher malignancy risk than non-smokers, as tobacco constituents can directly damage bronchial epithelium, induce oncogenic mutations, and impair immune-mediated clearance of malignant cells [77]. Third, patients with a history of cancer showed a 3.42-fold higher risk, possibly due to persistent pro-oncogenic microenvironments or treatment-induced secondary malignancies. A family history of cancer suggests a genetic predisposition to lung carcinogenesis [78]. Fourth, the observed protective effect in males contrasts with established epidemiological patterns and may reflect specific inclusion criteria. For example, female-predominant, non-smoking-related risk factors such as exposure to cooking oil fumes and EGFR mutations may be overrepresented in the studied population [79].

Radiographic characteristics—including nodule size, density, morphology, location, and calcification patterns—also showed strong associations with malignancy risk. Each 1-mm increase in nodule diameter corresponded to a 19% increase in malignancy risk, reflecting biological progression wherein tumors must reach critical size through sustained growth, with larger nodules more likely representing advanced malignant transformation [80]. Solid nodules showed a 4.58-fold greater malignancy risk compared to subsolid nodules, likely due to higher proliferative activity and greater invasive potential in solid tumor components, whereas ground-glass opacity nodules often represent pre-invasive or early-stage lesions [81]. Spiculation and lobulation emerged as pathognomonic signs of malignancy, explained by tumor cell dissemination along alveolar septa and lymphatic permeation disrupting lung architecture [82]. Nodules located in the upper lobes showed higher malignancy risk, possibly linked to greater ventilation volume and increased carcinogen deposition in upper lung fields [83]. Conversely, calcification and clear borders were characteristic of benign lesions, with calcification typically indicating chronic non-malignant conditions such as tuberculomas or hamartomas, and clear borders suggesting slow-growing, non-invasive lesions [84].

Several important limitations should be considered when interpreting these findings. First, the predominance of retrospective study designs introduces potential selection bias, as these studies typically rely on clinical databases with inherent patient selection processes. Second, we could not account for certain potentially important risk factors due to inconsistent reporting across primary studies, which may affect the precision of our risk estimates. Third, although we conducted extensive subgroup analyses to explore sources of heterogeneity, substantial unexplained variability remains, suggesting additional unmeasured moderating factors. Fourth, our meta-analysis cannot provide pooled baseline malignancy rates for specific size categories such as sub-centimeter nodules—a critical limitation reflecting that primary literature largely fails to report cross-tabulated data for size strata. Fifth, the scope of our risk factor analysis was necessarily constrained by available published literature. As per our pre-defined protocol, factors required data from at least three studies for inclusion in meta-analysis. Consequently, several emerging and clinically relevant parameters—notably nodule growth rate and various tumor biomarkers—could not be quantitatively synthesized. The assessment of growth rate through interval CT scanning represents a cornerstone of managing indeterminate nodules, while biomarkers are increasingly important for risk stratification. Their absence from our analysis reflects inconsistent reporting and lack of standardized data across primary observational studies rather than clinical unimportance. This gap highlights a critical need for future research: prospective SPN studies should systematically collect and report these dynamic and molecular markers to enable more comprehensive, evidence-based risk assessment. Sixth, while we performed analyses excluding all Chinese studies, we acknowledge that the overrepresentation of data from China warrants further exploration. Analyses of sub-geographic factors within China or more granular smoking patterns would be highly insightful; however, such analyses were not feasible as primary studies consistently lacked necessary patient-level or study-level data on these specific environmental and socio-economic variables. Reporting was insufficient to reliably classify studies by these criteria. Therefore, our analysis treats China as a single, high-risk entity, inevitably masking important internal heterogeneity. Investigating these sub-national risk differentials represents a crucial avenue for future primary research and more granular, individual-patient-data meta-analyses. Seventh, our analysis of calcification was limited. While we found that any calcification was a significant protective factor, we could not perform sub-analyses on specific calcification patterns (e.g. benign versus non-benign) as primary studies overwhelmingly reported it as a binary variable without morphological detail. Finally, our findings are limited by the nature of published literature. Selective reporting in original studies, underrepresentation of negative findings, and variability in diagnostic protocols across institutions may have influenced observed effects.

## 5. Conclusion

This meta-analysis confirms the importance of several established predictors for malignancy in incidental SPNs. Its primary novelty lies in providing quantitative, evidence-based refinement of their associations and highlighting critical sources of heterogeneity. First, we demonstrate that baseline risk is not uniform but varies significantly by geography, with notably higher pooled malignancy incidence in studies from China compared to other regions. This underscores the necessity for clinicians to consider epidemiological context when applying global incidence rates. Second, we provide robust, pooled estimates quantifying association strength for a comprehensive set of features. For instance, we not only confirm calcification as a protective feature but quantify its strong effect, enabling direct comparison with high-risk features such as solid nodule appearance or vascular convergence. This facilitates more nuanced, multi-factorial risk assessment than previously possible. Therefore, this work provides a refined, quantitative evidence base that supplements existing guidelines and clinical prediction models by highlighting regional disparities and offering precise estimates for a wide array of predictors.

## Supplementary Material

PRISMA_2020_checklist.docx

Supplementary file 1.docx

CLEAN manuscript.docx

Supplementary figures.docx

## Data Availability

All data generated or analysed during this study are included in this published article and its supplementary information files. The data synthesized and presented in the results section have been well-referenced as an update systematic review article. However, raw data used in the statistical analysis will be made available on request through the corresponding author.

## Systematic review registration

This study has been registered with in the INPLASY platform (number: INPLASY202570077).
