# Supplementary material for: Incidence and risk factors for malignancy in patients with incidental solitary pulmonary nodules: a systematic review and meta-analysis
Source: Ann Med. 2026 Feb 5;58(1):2596547. doi: 10.1080/07853890.2025.2596547 (PMC12879503; doi:10.1080/07853890.2025.2596547)
Supplement: Supplementary file 1.docx [file IANN_A_2596547_SM9105.docx]

**Database：PubMed:**

#1 "Solitary Pulmonary Nodule"[Mesh] OR "Solitary Pulmonary Nodule*"[tiab] OR "SPN"[tiab]

#2 (pulmonary OR lung OR chest OR pleura)[tiab] AND "SPN"[tiab]

#3 ((solitary OR coin OR single OR discrete OR indeterminate) NEAR/3 (lung OR pulmonary OR "chest wall" OR pleura) NEAR/3 (lesion* OR lump* OR nodule* OR lobe OR lobes))[tiab]

#4 #1 OR #2 OR #3

#5 "Lung Neoplasms"[Mesh] OR "lung cancer*"[tiab] OR "lung neoplasms*"[tiab]

#6 (Bronchopulmonary carcino* OR "Cancer of Lung" OR "Cancer of the Lung" OR "Lung adenocarcimoma*" OR "Lung Cancer*" OR "Lung carcinoma*" OR "Lung malignan*" OR "Lung Neoplasm*" OR "Lung Tumo*" OR "Pulmonary adenocarcinoma*" OR "Pulmonary Cancer*" OR "pulmonary carcino*" OR "pulmonary malignan*" OR "Pulmonary Neoplasm*" OR "Pulmonary tumo*")[tiab]

#7 ("Nonsmall Cell Lung Cancer*" OR "Non Small Cell Lung Cancer*" OR "Nonsmall Cell Lung Carcinoma*" OR "Non Small Cell Lung Carcinoma*" OR "NSCLC*")[tiab]

#8 ("Small Cell Lung Carcinoma*" OR "Oat Cell Carcinoma*" OR "Oat Cell Lung Cancer*" OR "SCLC*" OR "Small Cell Lung Cancer*" OR "Small Cell Lung Carcinoma*")[tiab]

#9 ("Pleural Neoplasms"[Mesh] OR "mpm*"[tiab] OR "Pleural cancer*"[tiab] OR "pleural malignan*"[tiab] OR "pleural mesothelioma*"[tiab] OR "Pleural Neoplasm*"[tiab] OR "pleural tumo*"[tiab])

#10 #5 OR #6 OR #7 OR #8 OR #9

#11 #4 OR #10

#12 ("Nodule size*"[tiab] OR diameter*[tiab])

#13 ("Solid*"[tiab] OR "Subsolid*"[tiab] OR "Non-solid*"[tiab] OR "Nonsolid*"[tiab] OR "NSN*"[tiab] OR "Part-solid*"[tiab] OR "Ground-glass*"[tiab] OR "GGO*"[tiab] OR "GGN*"[tiab])

#14 ("Smooth*"[tiab] OR "Lobulat*"[tiab] OR "Spiculat*"[tiab] OR "Border*"[tiab])

#15 ("Calcificat*"[tiab] OR "Pleural indentat*"[tiab] OR "Vessel Convergence*"[tiab] OR "Vascular sign*"[tiab] OR "Air bronchogram*"[tiab] OR "Bronchus sign*"[tiab] OR "Satellite lesion*"[tiab])

#16 ("Nodule locat*"[tiab] OR "Upper lobe*"[tiab] OR "Right lung*"[tiab] OR "Locat*"[tiab])

#17 ("Nodule growth rate*"[tiab] OR "Linear measure*"[tiab] OR "Semiautomated Nodule Volumet*"[tiab] OR "Volume Doubling Time*"[tiab])

#18 ("CT value*"[tiab] OR "Minimum Focal Density*"[tiab])

#19 ("Age"[Mesh] OR "Age factor*"[tiab] OR "Age*"[tiab])

#20 ("Sex"[Mesh] OR "Gender Identity"[Mesh] OR "Sex*"[tiab] OR "Gender*"[tiab] OR "Woman*"[tiab] OR "Women*"[tiab])

#21 ("Race"[Mesh] OR "Race factor*"[tiab] OR "Race*"[tiab] OR "Black man*"[tiab] OR "Black men*"[tiab])

#22 ("Family history*"[tiab] OR "Cancer history*"[tiab] OR "Medical history*"[tiab] OR "Health history*"[tiab])

#23 ("Neoplasm Metastasis"[Mesh] OR "Extrathoracic Malignant Neoplasm*"[tiab] OR "Extrathoracic disease*"[tiab])

#24 ("Pulmonary Disease, Chronic Obstructive"[Mesh] OR "Chronic Obstructive Pulmonary Disease*"[tiab] OR "Chronic Obstructive Lung Disease*"[tiab] OR "COPD*"[tiab] OR "Airflow Obstruction*"[tiab] OR "Emphysema*"[tiab])

#25 ("Idiopathic Pulmonary Fibrosis"[Mesh] OR "Idiopathic Pulmonary Fibros*"[tiab] OR "IPF*"[tiab] OR "Fibro*"[tiab] OR "Interstitial*"[tiab])

#26 ("Asthma"[Mesh] OR "Bronchiectasis"[Mesh] OR "Tuberculosis"[Mesh] OR "Asthma*"[tiab] OR "Bronchiectas*"[tiab] OR "Tuberculos*"[tiab] OR "Koch*"[tiab] OR "Mycobacterium*"[tiab])

#27 ("Smoking"[Mesh] OR "Smok*"[tiab] OR "Onset time*"[tiab] OR "Smoking Frequenc*"[tiab] OR "Cigarette quit*"[tiab] OR "Electronic cigarette*"[tiab] OR "Second-hand smoke*"[tiab] OR "cigarette*"[tiab])

#28 ("Alcohol Drinking"[Mesh] OR "Drinking*"[tiab] OR "Alcohol*"[tiab])

#29 ("Environmental Exposure"[Mesh] OR "Asbestos"[Mesh] OR "Uranium"[Mesh] OR "Radon"[Mesh] OR "Asbestos*"[tiab] OR "Uranium*"[tiab] OR "Radon*"[tiab] OR "Silica*"[tiab] OR "Silicon*"[tiab] OR "Cadmium*"[tiab] OR "Arsenic*"[tiab] OR "Beryllium*"[tiab] OR "Chromium*"[tiab] OR "Vehicle Emissions*"[tiab] OR "Diesel*"[tiab] OR "Nickel*"[tiab] OR "Coal Smke*"[tiab] OR "Soot*"[tiab] OR "Carbon*"[tiab] OR "Exposure*"[tiab])

#30 #12 OR #13 OR #14 OR #15 OR #16 OR #17 OR #18 OR #19 OR #20 OR #21 OR #22 OR #23 OR #24 OR #25 OR #26 OR #27 OR #28 OR #29

#31 "Tomography, X-Ray Computed"[Mesh] OR "Tomography, Spiral Computed"[Mesh] OR "computed tomography"[tiab]

#32 ("CT*"[tiab] OR "computed tomography*"[tiab] OR "computed tomographic scan*"[tiab] OR "computer assisted tomography*"[tiab] OR "computerised axial tomography*"[tiab] OR "computerised tomography*"[tiab] OR "computerized axial tomography*"[tiab] OR "computerized tomography*"[tiab])

#33 #31 OR #32

#34 #11 AND #30 AND #33

#35 "review"[Publication Type] OR "guideline"[Publication Type] OR "letter"[Publication Type] OR "editorial"[Publication Type] OR "comment"[Publication Type] OR "historical article"[Publication Type]

#36 #34 NOT #35

#37 "Animals"[Mesh] NOT "Humans"[Mesh]

#38 #36 NOT #37

#39 "Adult"[Mesh] OR (adult*[tiab] OR young[tiab] OR aged[tiab] OR elder*[tiab] OR older*[tiab] OR "18 years"[tiab] OR "18 yrs"[tiab])

#40 #38 AND #39

**Database：EMBASE**

#1 Solitary Pulmonary Nodule*.mp.

#2 SPN.tw.

#3 (pulmonary or lung or chest or pleura).tw.

#4 2 and 3

#5 ((solitary or coin or single or discrete or indeterminate) adj3 (lung or pulmonary or "chest wall" or pleura) adj3 (lesion* or lump* or nodule* or lobe or lobes)).tw.

#6 1 or 4 or 5

#7 (lung cancer* OR lung neoplasms*).mp.

#8 (Bronchopulmonary carcino* OR Cancer of Lung* OR Cancer of the Lung* OR Lung adenocarcimoma* OR Lung Cancer* OR Lung carcinoma* OR Lung malignan* OR Lung Neoplasm* OR Lung Tumo* OR Pulmonary adenocarcinoma* OR Pulmonary Cancer* OR pulmonary carcino* OR pulmonary malignan* OR Pulmonary Neoplasm* OR Pulmonary tumo*).mp.

#9 (Nonsmall Cell Lung Cancer* OR Non Small Cell Lung Cancer* OR Nonsmall Cell Lung Carcinoma* OR Non Small Cell Lung Carcinoma* OR NSCLC*).mp.

#10 (Small Cell Lung Carcinoma* OR Oat Cell Carcinoma* OR Oat Cell Lung Cancer* OR SCLC* OR Small Cell Lung Cancer* OR Small Cell Lung Carcinoma*).mp.

#11 (Pleural Neoplasms* OR mpm* OR Pleural cancer* OR pleural malignan* OR pleural mesothelioma* OR Pleural Neoplasm* OR pleural tumo*).mp.

#12 7 or 8 or 9 or 10 or 11

#13 6 or 12

#14 (Nodule size* or diameter*).mp.

#15 (Solid* or Subsolid* or Non-solid* or Nonsolid* or NSN* or Part-solid* or Ground-glass* or GGO* or GGN*).mp.

#16 (Smooth* or Lobulat* or Spiculat* or Border*).mp.

#17 (Calcificat* or Pleural indentat* or Vessel Convergence* or Vascular sign* or Air bronchogram* or Bronchus sign* Oor Satellite lesion*).mp.

#18 (Nodule locat* or Upper lobe* or Right lung* or Locat*).mp.

#19 (Nodule growth rate* or Linear measure* or Semiautomated Nodule Volumet* or Volume Doubling Time*).mp.

#20 (CT value* or Minimum Focal Density*).mp.

#21 (Age factor* or Age*).mp.

#22 (Gender Identity* or Sex* or Gender* or Woman* or Women*).mp.

#23 (Race factor* or Race* or Black man* or Black men*).mp.

#24 (Family history* or Cancer history* or Medical history* or Health history*).mp.

#25 (Extrathoracic Malignant Neoplasm* or Extrathoracic disease*).mp.

#26 (Pulmonary Disease, Chronic Obstructive* or Chronic Obstructive Pulmonary Disease* or Chronic Obstructive Lung Disease* or COPD* or Airflow Obstruction* or Emphysema*).mp.

#27 (Idiopathic Pulmonary Fibros* or IPF* or Fibro* or Interstitial*).mp.

#28 (Asthma* or Bronchiectas* or Tuberculos* or Koch* or Mycobacterium*).mp.

#29 (Smok* or Onset time* or Smoking Frequenc* or Cigarette quit* or Electronic cigarette* or Second-hand smoke* or cigarette*).mp.

#30 (Drinking* or Alcohol*).mp.

#31 (Asbestos* or Uranium* or Radon* or Silica* or Silicon* or Cadmium* or Arsenic* or Beryllium* or Chromium* or Vehicle Emissions* or Diesel* or Nickel* or Coal Smke* or Soot* or Carbon* or Exposure*).mp.

#32 #14 or #15 or #16 or #17 or #18 or #19 or #20 or #21 or #22 or #23 or #24 or #25 or #26 or #27 or #28 or #29 or #30 or #31

#33 Tomography, X-Ray Computed/

#34 Tomography, Spiral Computed/

#35 "computed tomography".tw.

#36 (CT* or computed tomography* or computed tomographic scan* or computer assisted tomography* or computerised axial tomography* or computerised tomography* or computerized axial tomography* or computerized tomography*).mp.

#37 #33 or #34 or #35 or #36

#38 #13 and #32 and #37

#39 (review or guideline or letter or editorial or comment or historical article).pt.

#40 #38 not #39

#41 exp animal/

#42 human/

#43 #41 not #42

#44 #40 not #43

#45 exp adult/

#46 (adult? or young or aged or elder* or older* or 18 years or 18 yrs).tw.

#47 #45 or #46

#48 #44 and #47

[mp=ti, ab, hw, tn, ot, dm, mf, dv, kw, fx, dq, nm, kf, px, rx, an, ui, sy]

**Database：Web of Science (All Databases)**

#1 TS= (Pulmonary nodule* OR lung nodule* OR SPN* OR Solitary Pulmonary Nodule*)

#2 TS= (lung cancer* OR lung neoplasms*)

#3 TS= (Bronchopulmonary carcino* OR Cancer of Lung* OR Cancer of the Lung* OR Lung adenocarcimoma* OR Lung Cancer* OR Lung carcinoma* OR Lung malignan* OR Lung Neoplasm* OR Lung Tumo* OR Pulmonary adenocarcinoma* OR Pulmonary Cancer* OR pulmonary carcino* OR pulmonary malignan* OR Pulmonary Neoplasm* OR Pulmonary tumo*)

#4 TS= (Carcinoma, Non‐Small‐Cell Lung* OR Nonsmall Cell Lung Cancer* OR Non Small Cell Lung Cancer* OR Nonsmall Cell Lung Carcinoma* OR Non Small Cell Lung Carcinoma* OR NSCLC*)

#5 TS= (Small Cell Lung Carcinoma* OR Oat Cell Carcinoma* OR Oat Cell Lung Cancer* OR SCLC* OR Small Cell Lung Cancer* OR Small Cell Lung Carcinoma*)

#6 TS= (Pleural Neoplasms* OR mpm* OR Pleural cancer* OR pleural malignan* OR pleural mesothelioma* OR Pleural Neoplasm* OR pleural tumo*)

#7 #1 OR #2 OR #3 OR #4 OR #5 OR #6

#8 TS= (Nodule size* OR diameter*)

#9 TS= (Solid* OR Subsolid* OR Non-solid* OR Nonsolid* OR NSN* OR Part-solid* OR Ground-glass* OR GGO* OR GGN*)

#10 TS= (Smooth* OR Lobulat* OR Spiculat* OR Border*)

#11 TS= (Calcificat* OR Pleural indentat* OR Vessel Convergence* OR Vascular sign* OR Air bronchogram* OR Bronchus sign* OR Satellite lesion*)

#12 TS= (Nodule locat* OR Upper lobe* OR Right lung* OR Locat*)

#13 TS= (Nodule growth rate* OR Linear measure* OR Semiautomated Nodule Volumet* OR Volume Doubling Time*)（

#14 TS= (CT value* OR Minimum Focal Density*)

#15 TS= (Age factors* OR Age*)

#16 TS= (Gender Identity* OR Gender* OR Sex* OR Woman* OR Women*)

#17 TS= (Race factor* OR Race* OR Black man* OR Black men*)

#18 TS= (Family history* OR Cancer history* OR Medical history* OR Health history*)

#19 TS= (Extrathoracic Malignant Neoplasm* OR Extrathoracic disease*)

#20 TS= (Pulmonary Disease, Chronic Obstructive* OR Chronic Obstructive Pulmonary Disease* OR Chronic Obstructive Lung Disease* OR COPD* OR Airflow Obstruction* OR Emphysema*)

#21 TS= (Idiopathic Pulmonary Fibros* OR IPF* OR Fibro* OR Interstitial*)

#22 TS= (Asthma* OR Bronchiectas* OR Tuberculos* OR Koch* OR Mycobacterium*)

#23 TS= (Smok* OR Onset time* OR Smoking Frequenc* OR Cigarette quit* OR Electronic cigarette* OR Second-hand smoke* OR cigarette*)

#24 TS= (Drinking* OR Alcohol*)

#25 TS= (Asbestos* OR Uranium* OR Radon* OR Silica* OR Silicon* OR Cadmium* Arsenic* OR Beryllium* OR Chromium* OR Vehicle Emissions* OR Diesel* OR Nickel* OR Coal Smke* OR Soot* OR Carbon* OR Exposure*)

#26 #8 OR#9 OR #10 OR #11 OR #12 OR #13 OR #14 OR #15 OR #16 OR #17 OR #18 OR #19 OR #20 OR #21 OR #22 OR #23 OR #24 OR #25

#27 TS= Tomography, X-Ray Compute*

#28 TS= (CT OR computed tomography* OR computed tomographic scan* OR computer assisted tomography* OR computerised axial tomography* OR computerised tomography* OR computerized axial tomography* OR computerized tomography*)

#29 #27 OR #28

#30 #7 AND #26 AND #29

#31 AB= (review or guideline or letter or editorial or comment or historical article)

#32 #30 NOT #31

#33 TS= Animal*

#34 TS= (Human* OR Man*)

#35 #33 NOT #34

#36 #32 NOT #35

#37 TS= (adult* OR aged*)

#38 AB= (young* OR middle aged* OR aged* OR elder* OR senior* OR older* OR 18 year* OR 18 yr*)

#39 #37 OR #38

#40 #36 AND #39

**Database：Cochrane Library**

#1 MeSH descriptor: [Solitary Pulmonary Nodule] explode all trees

#2 (Solitary Pulmonary Nodule* OR SPN):ti,ab,kw

#3 (pulmonary OR lung OR chest OR pleura):ti,ab,kw AND SPN:ti,ab,kw

#4 ((solitary OR coin OR single OR discrete OR indeterminate) NEAR/3 (lung OR pulmonary OR "chest wall" OR pleura) NEAR/3 (lesion* OR lump* OR nodule* OR lobe OR lobes)):ti,ab,kw

#5 #1 OR #2 OR #3 OR #4

#6 MeSH descriptor: [Lung Neoplasms] explode all trees

#7 (lung cancer* OR lung neoplasms* OR Bronchopulmonary carcino* OR "Cancer of Lung*" OR "Lung adenocarcimoma*" OR "Lung carcinoma*" OR "Lung malignan*" OR "Pulmonary adenocarcinoma*" OR "Pulmonary carcino*"):ti,ab,kw

#8 ("NSCLC*" OR "Non-small Cell Lung Cancer*" OR "SCLC*" OR "Small Cell Lung Cancer*"):ti,ab,kw

#9 MeSH descriptor: [Pleural Neoplasms] explode all trees

#10 #6 OR #7 OR #8 OR #9

#11 #5 OR #10

#12 ("Nodule size*" OR diameter* OR Solid* OR Subsolid* OR GGO* OR GGN*):ti,ab,kw

#13 (Smooth* OR Lobulat* OR Spiculat* OR Calcificat* OR "Air bronchogram*"):ti,ab,kw

#14 ("Nodule growth rate*" OR "Volume Doubling Time*" OR "CT value*"):ti,ab,kw

#15 MeSH descriptor: [Age Factors] OR (Age* OR elder* OR older*):ti,ab,kw

#16 MeSH descriptor: [Sex] OR (Sex* OR Gender* OR Women*):ti,ab,kw

#17 (Smok* OR "Cigarette*" OR "Alcohol*" OR Asbestos* OR Radon*):ti,ab,kw

#18 MeSH descriptor: [Pulmonary Disease, Chronic Obstructive] OR (COPD OR Emphysema):ti,ab,kw

#19 #12 OR #13 OR #14 OR #15 OR #16 OR #17 OR #18

#20 MeSH descriptor: [Tomography, X-Ray Computed] explode all trees

#21 ("CT" OR "computed tomography" OR "computerized tomography"):ti,ab,kw

#22 #20 OR #21

#23 #11 AND #19 AND #22

#24 NOT (review OR guideline OR letter):pt

#25 #23 NOT #24
