# Supplementary material for: Incidence and risk factors for malignancy in patients with incidental solitary pulmonary nodules: a systematic review and meta-analysis
Source: Ann Med. 2026 Feb 5;58(1):2596547. doi: 10.1080/07853890.2025.2596547 (PMC12879503; doi:10.1080/07853890.2025.2596547)
Supplement: CLEAN manuscript.docx [file IANN_A_2596547_SM9104.docx]

**Incidence and risk factors for malignancy in patients with incidental solitary pulmonary nodules: A systematic review and meta-analysis**

Huiyu Zheng^1*^, Zhipeng Shao^1^, Wensong Shi^1^, He Qian^1^, Yuchen Zhang^1^

^1^Thoracic Surgery Department/Zhengzhou People's Hospital/Zhengzhou, 450000, China.

***Corresponding author:** Huiyu Zheng, Zhengzhou People's Hospital, No. 33 Huanghe Road, Jinshui District, Zhengzhou City, Henan Province, China.

Email: Huiyu88@sina.com.

Tel: +86-13783615093

**Word counts: 2458**

**Abstract**

**Background:** The increasing use of chest imaging has led to a higher detection rate of incidental solitary pulmonary nodules (SPNs), often causing patient anxiety. Determining the malignancy rate and associated risk factors is crucial for developing appropriate follow-up strategies to prevent overdiagnosis, overtreatment, or missed diagnoses. This meta-analysis aims to investigate the malignancy rate and risk factors in patients with incidental SPNs.

**Methods:** A systematic search of PubMed, Embase, Web of Science, and the Cochrane Library was conducted up to June 30, 2025. Data on malignancy rates and potential risk factors were extracted from eligible studies. All pooled analyses were performed using a random-effects model.

**Results:** Fifty-four studies involving 19,985 patients were included. The pooled malignancy rate for incidental SPNs was 56.7% (95% CI: 51.5–62.0), with significant between-study heterogeneity (*I*² = 98.5%, *P* < 0.001). The pooled effect size showed a minimal change after adjustment for potential publication bias using the non-parametric Trim-and-Fill method (54.7%; 95%CI: 50.9–58.8). Risk factor analysis identified that older age, history of cancer, cigarette smoker, larger nodule diameter, spiculation, upper lobe location, lobulation, pleural indentation, vascular convergence, solid nodules, family history of cancer, and irregular or ill-defined margins were significantly associated with an increased risk of malignancy. Conversely, male sex, presence of calcification, and clear borders were significantly associated with a reduced risk of malignancy.

**Conclusion:** This meta-analysis provides a comprehensive assessment of malignancy rates and risk factors in incidental SPNs. The high pooled malignancy rate should be interpreted considering the significant heterogeneity and the inclusion of a high proportion of retrospective studies and populations from high-risk regions. Nonetheless, these findings offer essential evidence for clinical risk stratification, supporting optimized follow-up and informed decision-making.

**Keywords:** incidental solitary pulmonary nodules; malignancy; incidence; risk factors; meta-analysis

1. **Introduction**

Globally, lung cancer emerged as the leading malignancy in 2022, accounting for approximately 2.5 million new cases [1]. Studies reveal a dramatic disparity in outcomes: the 5-year survival rate ranges from 60% to 80% for stage I disease but drops sharply to just 12% for stage III C, underscoring the crucial importance of early diagnosis for patient prognosis [2,3]. With the widespread adoption of chest imaging technologies such as multi-detector computed tomography, the detection rate of incidental solitary pulmonary nodules (SPNs) has increased considerably in recent years [4]. SPNs are defined as incidentally discovered, well-circumscribed, round lesions measuring ≤ 3 cm in diameter on chest imaging, without associated abnormalities such as atelectasis, pneumonia, or lymphadenopathy [5].

This raising detection rate presents a dual clinical challenge. On one hand, some SPNs represent early-stage lung cancer, where delayed diagnosis can compromise treatment efficacy. On the other hand, most SPNs are benign; unnecessary testing or interventions may subject patients to physical and psychological burdens and increase healthcare costs [6]. Therefore, accurately assessing the malignancy risk of SPNs and identifying relevant risk factors is essential for clinical management.

Previous studies have explored various factors associated with SPN malignancy risk, including nodule characteristics (e.g., size, shape, density) and patient clinical features [7]. However, findings across studies often vary due to differences in study populations, sample sizes, and methodologies. For example, the malignancy risk threshold related to nodule size varies considerably, and the influence of smoking history may differ across populations [8]. This heterogeneity persists despite the development of several major clinical practice guidelines for managing incidental pulmonary nodules, such as those from the Fleischner Society, the British Thoracic Society (BTS), and the American College of Chest Physicians (ACCP) [9-11]. While these guidelines provide invaluable frameworks, they also reflect underlying uncertainties and the continuous evolution of evidence, particularly concerning the precise quantification of risk associated with specific factors in diverse populations. This ongoing challenge highlights the need for a synthesized, high-level evidence base.

In this context, a systematic review and meta-analysis of existing research can help overcome the limitations of individual studies by providing a more reliable estimate of SPN malignancy incidence and clarifying the true associations between risk factors and malignancy. This study aims to assess the overall malignancy rate in patients with incidental SPNs and to conduct an in-depth analysis of potential risk factors. The results will provide high-quality, evidence-based guidance to assist clinicians in risk stratification and the development of personalized follow-up and management strategies.

1. **Methods**
   1. **Data Sources, Search Strategy, and Selection Criteria**

This systematic review was conducted in full compliance with the PRISMA (Preferred Reporting Items for Systematic Reviews and Meta-Analyses) guidelines to ensure methodological rigor and transparency [12]. The study protocol was registered on the INPLASY platform (registration number: INPLASY202570077). Our objective was to comprehensively assess the incidence of malignancy and identify related risk factors in patients with incidental SPNs. We performed an extensive search for relevant epidemiological studies without restrictions on language or publication status, including published articles, unpublished data, and conference abstracts.

Four major databases—PubMed, Embase, Web of Science, and the Cochrane Library—were searched using core keywords and their variants, such as "SPNs," "malignancy," and "lung cancer." The search was updated through June 30, 2025, to include the most recent data. The complete search strategies for each database, including specific keyword combinations and filters, are provided in Supplementary File 1. To ensure comprehensive coverage, we also manually screened the reference lists of all identified primary studies and relevant review articles to identify any additional studies that might have been missed in the database searches.

Two reviewers independently conducted the literature screening and study selection using a standardized process. Discrepancies were resolved through discussions until consensus was reached. Studies were included if they met the following criteria: (1) Participants: patients with incidental SPNs (defined as rounded lesions ≤3 cm in diameter, detected via imaging without prior suspicion of malignancy); (2) Exposure: incidental SPNs confirmed as malignant or pathologically diagnosed as lung cancer; (3) Comparison: incidental SPNs confirmed as benign; (4) Outcomes: reported incidence of malignancy in incidental SPNs and associated risk factors. For a risk factor to be included in the meta-analysis, it had to be reported in at least three independent studies to ensure the stability and reliability of pooled estimates. The criteria for verifying benign and malignant nodules were pre-specified: malignancy required histopathological confirmation (via surgery or biopsy), while benign status was defined by either definitive histopathology; resolution or significant regression on follow-up imaging over at least 24 months; or characteristic benign calcification patterns confirmed by an experienced radiologist. Studies that did not apply comparable rigorous verification standards were excluded; (5) Study design: observational studies. Exclusion criteria were: (1) non-incidental SPNs; (2) insufficient confirmation of diagnosis; (3) incomplete data; and (4) significant imaging limitations.

To identify and eliminate duplicate publications or overlapping patient cohorts, we implemented a rigorous deduplication process after merging search results from all databases. For studies from the same institution or research group, we compared authors, patient recruitment periods, study center locations, sample sizes, and baseline cohort characteristics. In cases of suspected overlap, the study with the larger sample size, more comprehensive reporting, or longer follow-up was prioritized. If uncertainty remained, corresponding authors were contacted for clarification. No overlapping cohorts were identified in the final set of included studies.

- 1. **Data Collection and Quality Assessment**

Two reviewers independently performed the following tasks: (1) Data extraction, collecting information on first author, publication year, study design, country, sample size, mean participant age, sex distribution (proportion of males), smoking rate, number of benign and malignant SPNs, and diagnostic methods; (2) Quality assessment using the Newcastle-Ottawa Scale (NOS), which evaluates studies based on selection, comparability, and outcome assessment [13]. All extracted data and quality assessment results were cross-checked. Disagreements were resolved by a third reviewer through verification of the original literature and discussion, ensuring data accuracy and consistency in quality assessment.

- 1. **Statistical Analysis**

We performed a random-effects meta-analysis to estimate the pooled incidence of malignancy in incidental SPNs. To enhance data comparability, all raw data were log-transformed prior to analysis [14]. Restricted maximum likelihood estimation was applied to improve the accuracy of parameter estimates. Effect sizes for factors associated with malignancy were expressed as odds ratios (ORs) with 95% confidence intervals (CIs), pooled using the random-effects model [14].

Between-study heterogeneity was assessed using the *I^2^* statistic and Q-test, with*I^2^* ≥ 50% or a Q-test *P*-value < 0.10 indicating significant heterogeneity [15,16]. Sensitivity analyses were performed by sequentially excluding individual studies to evaluate the robustness of the results [17]. Subgroup analyses were conducted based on publication year, study design, geographic region, and study quality to explore potential sources of heterogeneity. Differences between subgroups were compared using interaction t-tests, assuming a normal distribution of the analytical data [18]. For sources of heterogeneity that could not be quantitatively assessed via meta-regression due to insufficient primary data, a qualitative assessment was performed by examining the methodologies of the included studies. Publication bias was assessed using both qualitative (funnel plot) and quantitative (Egger’s and Begg’s tests) methods [19,20]. All statistical tests were two-sided, with a significance threshold of *P* < 0.05. Data analysis was performed using STATA 18.0 (StataCorp, College Station, TX, USA).

1. **Results**
   1. **Literature Search**

Our systematic search identified 18,934 records from electronic databases. After removing duplicates, 12,567 unique studies underwent title/abstract screening, which led to the exclusion of 12,236 irrelevant publications. A full-text review of the remaining 331 potentially eligible studies resulted in the exclusion of 277 that did not meet the inclusion criteria. The final meta-analysis included 54 studies [21-74], with no additional eligible studies identified through manual screening of reference lists. The complete study selection process is illustrated in Figure 1.

- 1. **Study Characteristics**
     The baseline characteristics of the 54 included studies, encompassing 19,985 patients, are summarized in Table 1. Sample sizes ranged from 107 to 1,679 participants. The majority of studies were retrospective cohort designs (n=52), while two were prospective cohort studies. Geographically, 45 studies were conducted in China, with the remaining nine originating from seven other countries: USA, Switzerland, Spain, Korea, Turkey, Portugal, and Greece. Quality assessment using the NOS yielded scores of 8 points (n=5), 7 points (n=29), and 6 points (n=20) points.
  2. **Incidence of Malignancy in Patients with Incidental SPNs**
     The pooled malignancy rate for incidental SPNs was 56.7% (95% CI: 51.5–62.0; Figure 2). Given the substantial heterogeneity (*I^2^* = 98.5%, *P* < 0.001), the 95% prediction interval, which estimates the range within which the true incidence in a new, similar study would fall, was wide, ranging from 31.8% to 78.9%. Sensitivity analysis demonstrated stable estimates, with malignancy rates ranging from 56.1% to 57.4% upon sequential exclusion of individual studies (Supplementary Figure S1). To directly address the geographical distribution of our sample, a subgroup analysis was performed comparing studies from China with studies from other countries. The pooled malignancy incidence was significantly higher in the Chinese studies (57.7% [95% CI: 52.4–63.0]) compared to the non-Chinese studies (51.9% [95% CI: 36.2–67.5]) (*P* for subgroup difference < 0.001). Additional subgroup analyses revealed the following trends: (1) studies published after 2020 reported slightly higher malignancy risks; (2) retrospective cohort studies showed significantly higher malignancy rates than prospective cohorts; (3) the highest malignancy risk was observed in studies from Korea, while the lowest was reported in studies from Spain; and (4) studies with lower NOS quality scores tended to report higher malignancy risks compared to those with high scores (Supplementary Figures S2-S5). A qualitative assessment of methodological variations across the included studies identified further potential sources of heterogeneity. Although the core definition of an SPN was consistently applied, operational details often differed, including variations in imaging protocols, generations of CT scanners used, and methods for measuring nodule diameter. These inconsistencies in diagnostic criteria and imaging techniques, which were not uniformly reported and thus could not be included in a quantitative analysis, likely contributed substantially to the observed heterogeneity. Publication bias assessment showed a non-significant Begg's test (*P*=0.952) but a significant Egger's test (*P*=0.042), suggesting potential small-study effects (Figure 3A). We applied the non-parametric Trim-and-Fill method to adjust for potential publication bias. The adjusted pooled malignancy rate was 54.7% (95% CI: 50.9–58.8), which represents a minimal change from the original estimate of 56.7% (Figure 3B).
  3. **Risk Factors for Malignancy in Patients with Incidental SPNs**
     We systematically analyzed factors associated with malignancy risk in incidental SPNs (Table 2 and Supplementary Figures S6-S20). Several factors were significantly associated with an increased risk of malignancyk: older age (OR: 1.04; 95% CI: 1.03–1.06; *P* < 0.001), history of cancer (OR: 3.42; 95% CI: 1.61–7.24; *P* = 0.001), cigarette smoker (OR: 2.12; 95% CI: 1.53–2.95; *P* < 0.001), larger nodule diameter (OR: 1.19; 95% CI: 1.13–1.25; *P* < 0.001), spiculation (OR: 3.67; 95% CI: 2.86–4.72; *P* < 0.001), upper lobe location (OR: 1.78; 95% CI: 1.25–2.54; *P* = 0.001), lobulation (OR: 3.84; 95% CI: 2.79–5.29; *P* < 0.001), pleural indentation (OR: 2.84; 95% CI: 2.24–3.60; *P* < 0.001), vascular convergence (OR: 4.32; 95% CI: 3.03–6.16; *P* < 0.001), solid nodule appearance (OR: 4.58; 95% CI: 2.87–7.30; *P* < 0.001), family history of cancer (OR: 3.59; 95% CI: 1.90–6.78; *P* < 0.001), and irregular or ill-defined margins (OR: 2.22; 95% CI: 1.28–3.83; *P* = 0.004). Conversely, male sex (OR: 0.58; 95% CI: 0.38–0.89; *P* = 0.012), presence of calcification (OR: 0.21; 95% CI: 0.10–0.46; *P* < 0.001), and clear borders (OR: 0.19; 95% CI: 0.12–0.29; *P* < 0.001) were significantly associated with a reduced risk of malignancy. Air bronchogram, and satellite lesions showed no significant association with malignancy risk.

Significant heterogeneity was observed for several factors, including age (*I^2^* = 81.2%, *P* < 0.001), sex (*I^2^* = 76.3%, *P* < 0.001), history of cancer (*I^2^* = 87.0%, *P* < 0.001), cigarette smoker (*I^2^* = 80.0%, *P* < 0.001), nodule diameter (*I^2^* = 88.4%, *P* < 0.001), spiculation (*I^2^* = 77.7%, *P* < 0.001), upper lobe location (*I^2^* = 84.9%, *P* < 0.001), calcification (*I^2^* = 85.8%, *P* < 0.001), lobulation (*I^2^* = 67.4%, *P* < 0.001), clear border (*I^2^* = 57.8%, *P* = 0.011), air bronchogram (*I^2^* = 83.0%, *P* < 0.001), and satellite lesions (*I^2^* = 75.8%, *P* = 0.016).

Sensitivity analysis indicated that the association for air bronchogram became statistically significant after excluding the study by Wang et al. [41]. For all other identified risk factors, the associations remained robust even in sensitivity analyses (Supplementary Figures S21-S34).

Publication bias assessment for individual risk factors is presented in Supplementary Figures S35-S48. The results indicated potential publication bias for history of cancer, cigarette smoker, nodule diameter, spiculation, upper lobe location, and vascular convergence. However, after adjusting for potential publication bias, the overall conclusions for these factors remained unchanged.

1. **Discussion**

This study, comprising 54 studies with a total of 19,985 patients, represents the first large-scale comprehensive assessment of malignancy incidence and associated risk factors in incidental SPNs. The pooled analysis showed an overall malignancy rate of 56.7%, with several patient characteristics and radiographic features significantly associated with malignancy risk.

Our pooled malignancy rate of 56.7% is notably higher than rates often cited in older literature. While advancements in CT imaging resolution may contribute to the detection of earlier-stage malignancies [75], clinical setting and population-specific risk factors appear to be more influential drivers of this elevated estimate. The observed gradient from screening cohorts (lowest risk) to surgical series (highest risk) demonstrates that clinical context is a paramount determinant of pre-test probability. Furthermore, the significant difference in malignancy incidence between Chinese and non-Chinese studies, coupled with our finding that study-level characteristics significantly predict malignancy rates, strongly implicates geographical and epidemiological factors as primary contributors.

We observed substantial heterogeneity across studies, attributable to several factors. Our subgroup analysis confirmed significantly higher malignancy incidence in Chinese populations compared to non-Chinese populations, underscoring the profound impact of regional factors on SPN malignancy risk. The elevated rate in Chinese studies may reflect a combination of high prevalence of risk factors such as smoking and environmental exposures, alongside genetic predispositions to certain lung cancer subtypes more common in Asian populations. This reinforces that our pooled global estimate should be applied cautiously, with clinicians prioritizing local or regional data when available for pre-test probability assessment. Retrospective cohort studies reported significantly higher malignancy rates compared to prospective studies, likely because retrospective designs typically rely on clinical databases that disproportionately include patients with more suspicious nodule characteristics. These patients are more likely to undergo intensive evaluation, potentially inflating malignancy estimates. In contrast, prospective studies generally encompass a broader spectrum of nodules, including lower-risk cases that may not require invasive evaluation, thus providing estimates more representative of the general population. Additionally, studies with lower methodological quality reported higher malignancy rates, possibly reflecting non-standardized data collection methods and inconsistent diagnostic criteria. Finally, clinical implications of this substantial heterogeneity are significant. The pooled malignancy rate of 56.7% should not be interpreted as a universal probability for every patient with an SPN. Instead, our findings emphasize that pre-test probability of malignancy is highly context-dependent. Therefore, the primary clinical value of this meta-analysis lies not in the aggregate incidence rate, but in the consistent direction and magnitude of associations we identified for specific risk factors. For instance, despite heterogeneity, the presence of spiculation or larger nodule size consistently and strongly predicted malignancy across diverse settings, reinforcing these imaging features as robust biomarkers of risk. Consequently, clinicians should utilize our results as a validated risk stratification framework rather than a precise calculator. The identified risk and protective factors provide a powerful tool to guide shared decision-making, helping triage patients toward appropriate diagnostic pathways or active surveillance, while integrating these factors with patient-specific context and local expertise.

Patient-related factors significantly associated with increased malignancy risk included older age, cigarette smoker, personal cancer history, and family history of cancer. Conversely, male sex appeared to have a protective effect. The underlying mechanisms for these associations may be explained as follows: First, each additional year of age was associated with a 4% increase in malignancy risk, likely due to age-related declines in DNA repair capacity, cumulative genetic mutations, prolonged carcinogen exposure, and weakened immune surveillance in older individuals [76]. Second, smokers had a 2.12-fold higher malignancy risk than non-smokers, as tobacco constituents can directly damage bronchial epithelium, induce oncogenic mutations, and impair immune-mediated clearance of malignant cells [77]. Third, patients with a history of cancer showed a 3.42-fold higher risk, possibly due to persistent pro-oncogenic microenvironments or treatment-induced secondary malignancies. A family history of cancer suggests a genetic predisposition to lung carcinogenesis [78]. Fourth, the observed protective effect in males contrasts with established epidemiological patterns and may reflect specific inclusion criteria. For example, female-predominant, non-smoking-related risk factors such as exposure to cooking oil fumes and EGFR mutations may be overrepresented in the studied population [79].

Radiographic characteristics—including nodule size, density, morphology, location, and calcification patterns—also showed strong associations with malignancy risk. Each 1-mm increase in nodule diameter corresponded to a 19% increase in malignancy risk, reflecting biological progression wherein tumors must reach critical size through sustained growth, with larger nodules more likely representing advanced malignant transformation [80]. Solid nodules showed a 4.58-fold greater malignancy risk compared to subsolid nodules, likely due to higher proliferative activity and greater invasive potential in solid tumor components, whereas ground-glass opacity nodules often represent pre-invasive or early-stage lesions [81]. Spiculation and lobulation emerged as pathognomonic signs of malignancy, explained by tumor cell dissemination along alveolar septa and lymphatic permeation disrupting lung architecture [82]. Nodules located in the upper lobes showed higher malignancy risk, possibly linked to greater ventilation volume and increased carcinogen deposition in upper lung fields [83]. Conversely, calcification and clear borders were characteristic of benign lesions, with calcification typically indicating chronic non-malignant conditions such as tuberculomas or hamartomas, and clear borders suggesting slow-growing, non-invasive lesions [84].

Several important limitations should be considered when interpreting these findings. First, the predominance of retrospective study designs introduces potential selection bias, as these studies typically rely on clinical databases with inherent patient selection processes. Second, we could not account for certain potentially important risk factors due to inconsistent reporting across primary studies, which may affect the precision of our risk estimates. Third, although we conducted extensive subgroup analyses to explore sources of heterogeneity, substantial unexplained variability remains, suggesting additional unmeasured moderating factors. Fourth, our meta-analysis cannot provide pooled baseline malignancy rates for specific size categories such as sub-centimeter nodules—a critical limitation reflecting that primary literature largely fails to report cross-tabulated data for size strata. Fifth, the scope of our risk factor analysis was necessarily constrained by available published literature. As per our pre-defined protocol, factors required data from at least three studies for inclusion in meta-analysis. Consequently, several emerging and clinically relevant parameters—notably nodule growth rate and various tumor biomarkers—could not be quantitatively synthesized. The assessment of growth rate through interval CT scanning represents a cornerstone of managing indeterminate nodules, while biomarkers are increasingly important for risk stratification. Their absence from our analysis reflects inconsistent reporting and lack of standardized data across primary observational studies rather than clinical unimportance. This gap highlights a critical need for future research: prospective SPN studies should systematically collect and report these dynamic and molecular markers to enable more comprehensive, evidence-based risk assessment. Sixth, while we performed analyses excluding all Chinese studies, we acknowledge that the overrepresentation of data from China warrants further exploration. Analyses of sub-geographic factors within China or more granular smoking patterns would be highly insightful; however, such analyses were not feasible as primary studies consistently lacked necessary patient-level or study-level data on these specific environmental and socio-economic variables. Reporting was insufficient to reliably classify studies by these criteria. Therefore, our analysis treats China as a single, high-risk entity, inevitably masking important internal heterogeneity. Investigating these sub-national risk differentials represents a crucial avenue for future primary research and more granular, individual-patient-data meta-analyses. Seventh, our analysis of calcification was limited. While we found that any calcification was a significant protective factor, we could not perform sub-analyses on specific calcification patterns (e.g., benign versus non-benign) as primary studies overwhelmingly reported it as a binary variable without morphological detail. Finally, our findings are limited by the nature of published literature. Selective reporting in original studies, underrepresentation of negative findings, and variability in diagnostic protocols across institutions may have influenced observed effects.

1. **Conclusion**

This meta-analysis confirms the importance of several established predictors for malignancy in incidental SPNs. Its primary novelty lies in providing quantitative, evidence-based refinement of their associations and highlighting critical sources of heterogeneity. First, we demonstrate that baseline risk is not uniform but varies significantly by geography, with notably higher pooled malignancy incidence in studies from China compared to other regions. This underscores the necessity for clinicians to consider epidemiological context when applying global incidence rates. Second, we provide robust, pooled estimates quantifying association strength for a comprehensive set of features. For instance, we not only confirm calcification as a protective feature but quantify its strong effect, enabling direct comparison with high-risk features such as solid nodule appearance or vascular convergence. This facilitates more nuanced, multi-factorial risk assessment than previously possible. Therefore, this work provides a refined, quantitative evidence base that supplements existing guidelines and clinical prediction models by highlighting regional disparities and offering precise estimates for a wide array of predictors.

**Acknowledgements:** Not applicable.

**Author’s contributions:** Huiyu Zheng: Conceptualization, methodology, software, validation, formal analysis, investigation, data curation, writing–original draft preparation, writing–review and editing, visualization, supervision, project administration. Zhipeng Shao: Methodology, software, investigation, data curation, writing–review and editing, visualization. Wensong Shi: Software, formal analysis, writing–review and editing. He Qian: Software, formal analysis, writing–review and editing. Yuchen Zhang: Validation, writing–review and editing. All authors read and approval the final version of the manuscript.

**Funding Statement:** This research was not sponsored or funded.

**Systematic review registration:** This study has been registered with in the INPLASY platform (number: INPLASY202570077).

**Disclosure statement:** No potential conflict of interest was reported by the author(s).

**Ethics approval and consent to participate:** Not applicable.

**Consent for publication:** Not applicable.

**Clinical trial number:** Not applicable.

**Data availability statement:** All data generated or analysed during this study are included in this published article and its supplementary information files. The data synthesized and presented in the results section have been well-referenced as an update systematic review article. However, raw data used in the statistical analysis will be made available on request through the corresponding author.

**Reference**

1. Bray F, Laversanne M, Sung H, Ferlay J, Siegel RL, Soerjomataram I, *et al*. Global cancer statistics 2022: GLOBOCAN estimates of incidence and mortality worldwide for 36 cancers in 185 countries. *CA Cancer J Clin.* 2024;74(3):229-263. doi: 10.3322/ caac.21834.
2. Goldstraw P, Chansky K, Crowley J, Rami-Porta R, Asamura H, Eberhardt WE, *et al*. The IASLC Lung Cancer Staging Project: Proposals for Revision of the TNM Stage Groupings in the Forthcoming (Eighth) Edition of the TNM Classification for Lung Cancer. *J Thorac Oncol.* 2016;11(1):39-51. doi: 10.1016/j.jtho.2015.09.009.
3. Bray F, Ferlay J, Soerjomataram I, Siegel RL, Torre LA, Jemal A. Global cancer statistics 2018: GLOBOCAN estimates of incidence and mortality worldwide for 36 cancers in 185 countries. *CA Cancer J Clin.* 2018;68(6):394-424. doi: 10.3322/caac. 21492.
4. Toghiani A, Adibi A, Taghavi A. Significance of pulmonary nodules in multi-detector computed tomography scan of noncancerous patients. *J Res Med Sci.* 2015;20(5):460-4. doi: 10.4103/1735-1995.163967.
5. Nead MA, Dony C, Rivera MP. Diagnosis of the Solitary Pulmonary Nodule. *Clin Chest Med.* 2025;46(2):271-288. doi: 10.1016/j.ccm.2025.02.006.
6. Wyker A, Sharma S, Henderson WW. Solitary Pulmonary Nodule. 2024 Aug 12. In: StatPearls [Internet]. Treasure Island (FL): StatPearls Publishing; 2025 Jan–.
7. Yang Y, Li X, Duan Y, Zhao J, Huang Q, Zhou C, *et al*. Risk factors for malignant solid pulmonary nodules: a meta-analysis. *BMC Cancer.* 2025;25(1):312. doi: 10.1186/s12885-025-13702-2.
8. Chen B, Li Q, Hao Q, Tan J, Yan L, Zhu Y, *et al*. Malignancy risk stratification for solitary pulmonary nodule: A clinical practice guideline. *J Evid Based Med.* 2022; 15(2):142-151. doi: 10.1111/jebm.12476.
9. MacMahon H, Naidich DP, Goo JM, Lee KS, Leung ANC, Mayo JR, *et al*. Guidelines for Management of Incidental Pulmonary Nodules Detected on CT Images: From the Fleischner Society 2017. *Radiology.* 2017;284(1):228-243. doi: 10.1148/ radiol.2017161659.
10. Neumann K, Berg J, Ashraf H, Isaksson J, Aija Knuuttila, Borg MH, *et al*. Adherence to guidelines for incidental pulmonary nodules: insights from a Nordic survey. *Acta Oncol*. 2025;64:22-26. doi: 10.2340/1651-226X.2025.42461.
11. Expert Panel on Thoracic Imaging; Martin MD, Henry TS, Berry MF, Johnson GB, Kelly AM, Ko JP, *et al*. ACR Appropriateness Criteria® Incidentally Detected Indeterminate Pulmonary Nodule. *J Am Coll Radiol.* 2023;20(11S):S455-S470. doi: 10.1016/j.jacr.2023.08.024.
12. Page MJ, McKenzie JE, Bossuyt PM, Boutron I, Hoffmann TC, Mulrow CD, *et al*. The PRISMA 2020 statement: an updated guideline for reporting systematic reviews. *BMJ.* 2021;372:n71. doi: 10.1136/bmj.n71.
13. Wells G, Shea B, O’Connell D. The Newcastle-Ottawa Scale (NOS) for assessing the quality of nonrandomised studies in meta-analyses. *Ottawa (ON): Ottawa Hospital Research Institute* 2009.Available:[http://www.ohri.ca/programs/clinical_ epidemiology/oxford.htm](http://www.ohri.ca/programs/clinical_epidemiology/oxford.htm).
14. DerSimonian R, Laird N. Meta-analysis in clinical trials. *Control Clin Trials.* 1986;7(3):177-88. doi: 10.1016/0197-2456(86)90046-2.
15. Deeks JJ, Higgins JPT, Altman DG. Analyzing data and undertaking meta-analyses. In: Higgins J, Green S, eds. Cochrane Handbook for Systematic Reviews of Interventions 5.0.1. Oxford, UK: The Cochrane Collaboration: 2008; chap 9.
16. Higgins JP, Thompson SG, Deeks JJ, Altman DG. Measuring inconsistency in meta-analyses. *BMJ.* 2003;327(7414):557-60. doi: 10.1136/bmj.327.7414.557.
17. Tobias A. Assessing the influence of a single study in meta-analysis. *Stata Tech Bull.* 1999; 47: 15-17.
18. Altman DG, Bland JM. Interaction revisited: the difference between two estimates. *BMJ*. 2003;326(7382):219. doi: 10.1136/bmj.326.7382.219.
19. Egger M, Davey Smith G, Schneider M, Minder C. Bias in meta-analysis detected by a simple, graphical test. *BMJ*. 1997;315(7109):629-34. doi: 10.1136/bmj. 315.7109.629.
20. Begg CB, Mazumdar M. Operating characteristics of a rank correlation test for publication bias. *Biometrics*. 1994;50(4):1088-101.
21. Swensen SJ, Silverstein MD, Ilstrup DM, Schleck CD, Edell ES. The probability of malignancy in solitary pulmonary nodules. Application to small radiologically indeterminate nodules. *Arch Intern Med.* 1997;157(8):849-55. doi: 10.1001/archinte. 1997.00440290031002.
22. Gould MK, Ananth L, Barnett PG; Veterans Affairs SNAP Cooperative Study Group. A clinical model to estimate the pretest probability of lung cancer in patients with solitary pulmonary nodules. *Chest.* 2007;131(2):383-8. doi: 10.1378/chest. 06-1261.
23. Yang D, Li Y, Liu J, Jiang G, Li J, Zhao H, *et al*. [Study on solitary pulmonary nodules: correlation between diameter and clinical manifestation and pathological features]. *Zhongguo Fei Ai Za Zhi.* 2010;13(6):607-11. Chinese. doi: 10.3779/j.issn. 1009-3419.2010.06.008.
24. Li Y, Wang J. A mathematical model for predicting malignancy of solitary pulmonary nodules. *World J Surg.* 2012;36(4):830-5. doi: 10.1007/s00268-012-1449 -8.
25. Dong J, Sun N, Li J, Liu Z, Zhang B, Chen Z, *et al*. Development and validation of clinical diagnostic models for the probability of malignancy in solitary pulmonary nodules. *Thorac Cancer.* 2014;5(2):162-8. doi: 10.1111/1759-7714.12077.
26. Shi CZ, Zhao Q, Luo LP, He JX. Size of solitary pulmonary nodule was the risk factor of malignancy. *J Thorac Dis.* 2014;6(6):668-76. doi: 10.3978/j.issn.2072-1439. 2014.06.22.
27. Gómez-Sáez N, Hernández-Aguado I, Vilar J, González-Alvarez I, Lorente MF, Domingo ML, *et al*. Lung cancer risk and cancer-specific mortality in subjects undergoing routine imaging test when stratified with and without identified lung nodule on imaging study. *Eur Radiol.* 2015;25(12):3518-27. doi: 10.1007/s00330- 015-3775-3.
28. Zhang M, Zhuo N, Guo Z, Zhang X, Liang W, Zhao S, *et al*. Establishment of a mathematic model for predicting malignancy in solitary pulmonary nodules. *J Thorac Dis.* 2015;7(10):1833-41. doi: 10.3978/j.issn.2072-1439.2015.10.56.
29. Zheng B, Zhou X, Chen J, Zheng W, Duan Q, Chen C. A Modified Model for Preoperatively Predicting Malignancy of Solitary Pulmonary Nodules: An Asia Cohort Study. *Ann Thorac Surg.* 2015;100(1):288-94. doi: 10.1016/j.athoracsur.2015. 03.071.
30. Choi SM, Heo EY, Lee J, Park YS, Lee CH, Park CM, *et al*. Characteristics of benign solitary pulmonary nodules confirmed by diagnostic video-assisted thoracoscopic surgery. *Clin Respir J.* 2016;10(2):181-8. doi: 10.1111/crj.12200.
31. Hanauer M, Perentes JY, Krueger T, Ris HB, Bize P, Schmidt S, *et al*. Pre-operative localization of solitary pulmonary nodules with computed tomography-guided hook wire: report of 181 patients. *J Cardiothorac Surg.* 2016;11:5. doi: 10.1186/s13019-016-0404-4.
32. Hu H, Wang Q, Tang H, Xiong L, Lin Q. Multi-slice computed tomography characteristics of solitary pulmonary ground-glass nodules: Differences between malignant and benign. *Thorac Cancer.* 2016;7(1):80-7. doi: 10.1111/1759-7714. 12280.
33. Yu W, Ye B, Xu L, Wang Z, Le H, Wang S, *et al*. [Establishment of A Clinical Prediction Model of Solid Solitary Pulmonary Nodules]. *Zhongguo Fei Ai Za Zhi.* 2016;19(10):705-710. Chinese. doi: 10.3779/j.issn.1009-3419.2016.10.12.
34. Li M, Han R, Song W, Wang X, Guo F, Su D, *et al*. [Three Dimensional Volumetric Analysis of Solid Pulmonary Nodules on Chest CT: Cancer Risk Assessment]. *Zhongguo Fei Ai Za Zhi.* 2016;19(5):279-85. Chinese. doi: 10.3779/j. issn.1009-3419.2016.05.05.
35. Xiang Y, Sun Y, Gao W, Han B, Chen Q, Ye X, *et al*. [Establishment of a predicting model to evaluate the probability of malignancy or benign in patients with solid solitary pulmonary nodules]. *Zhonghua Yi Xue Za Zhi.* 2016;96(17):1354-8. Chinese. doi: 10.3760/cma.j.issn.0376-2491.2016.17.011.
36. Bellier J, Perentes JY, Abdelnour-Berchtold E, Lopez B, Krueger T, Beigelman-Aubry C, *et al*. A plea for thoracoscopic resection of solitary pulmonary nodule in cancer patients. *Surg Endosc.* 2017;31(11):4705-4710. doi: 10.1007/ s00464-017-5543-8.
37. Ma J, Guarnera MA, Zhou W, Fang H, Jiang F. A Prediction Model Based on Biomarkers and Clinical Characteristics for Detection of Lung Cancer in Pulmonary Nodules. *Transl Oncol.* 2017;10(1):40-45. doi: 10.1016/j.tranon.2016.11.001.
38. She Y, Zhao L, Dai C, Ren Y, Jiang G, Xie H, *et al*. Development and validation of a nomogram to estimate the pretest probability of cancer in Chinese patients with solid solitary pulmonary nodules: A multi-institutional study. *J Surg Oncol.* 2017; 116(6):756-762. doi: 10.1002/jso.24704.
39. She Y, Zhao L, Dai C, Ren Y, Zha J, Xie H, *et al*. Preoperative nomogram for identifying invasive pulmonary adenocarcinoma in patients with pure ground-glass nodule: A multi-institutional study. *Oncotarget.* 2017;8(10):17229-17238. doi: 10. 18632/oncotarget.11236.
40. Yang L, Zhang Q, Bai L, Li TY, He C, Ma QL, *et al*. Assessment of the cancer risk factors of solitary pulmonary nodules. *Oncotarget.* 2017;8(17):29318-29327. doi: 10.18632/oncotarget.16426.
41. Wang L, Shen W, Xi Y, Liu S, Zheng D, Jin C. Nomogram for Predicting the Risk of Invasive Pulmonary Adenocarcinoma for Pure Ground-Glass Nodules. *Ann Thorac Surg.* 2018;105(4):1058-1064. doi: 10.1016/j.athoracsur.2017.11.012.
42. Wang X, Xu YH, Du ZY, Qian YJ, Xu ZH, Chen R, *et al*. [Risk factor analysis of the patients with solitary pulmonary nodules and establishment of a prediction model for the probability of malignancy]. *Zhonghua Zhong Liu Za Zhi.* 2018;40(2):115-120. Chinese. doi: 10.3760/cma.j.issn.0253-3766.2018.02.007.
43. Yang W, Sun Y, Fang W, Qian F, Ye J, Chen Q, *et al*. High-resolution Computed Tomography Features Distinguishing Benign and Malignant Lesions Manifesting as Persistent Solitary Subsolid Nodules. *Clin Lung Cancer.* 2018;19(1):e75-e83. doi: 10.1016/j.cllc.2017.05.023.
44. Chen XB, Yan RY, Zhao K, Zhang DF, Li YJ, Wu L, *et al*. Nomogram For The Prediction Of Malignancy In Small (8-20 mm) Indeterminate Solid Solitary Pulmonary Nodules In Chinese Populations. *Cancer Manag Res.* 2019;11:9439-9448. doi: 10.2147/CMAR.S225739.
45. Xiao F, Yu Q, Zhang Z, Liu D, Liang C. [Establishment and Verification of A Novel Predictive Model of Malignancy for Non-solid Pulmonary Nodules]. *Zhongguo Fei Ai Za Zhi.* 2019;22(1):26-33. Chinese. doi: 10.3779/j.issn.1009-3419. 2019.01.06.
46. Chen W, Zhu D, Chen H, Luo J, Fu H. Predictive model for the diagnosis of benign/malignant small pulmonary nodules. *Medicine (Baltimore).* 2020;99(15): e19452. doi: 10.1097/MD.0000000000019452.
47. Chen X, Feng B, Chen Y, Liu K, Li K, Duan X, *et al*. A CT-based radiomics nomogram for prediction of lung adenocarcinomas and granulomatous lesions in patient with solitary sub-centimeter solid nodules. *Cancer Imaging.* 2020;20(1):45. doi: 10.1186/s40644-020-00320-3.
48. Feng B, Chen X, Chen Y, Lu S, Liu K, Li K, *et al*. Solitary solid pulmonary nodules: a CT-based deep learning nomogram helps differentiate tuberculosis granulomas from lung adenocarcinomas. *Eur Radiol.* 2020;30(12):6497-6507. doi: 10.1007/s00330-020-07024-z.
49. Zhang R, Tian P, Chen B, Zhou Y, Li W. Predicting Lung Cancer Risk of Incidental Solid and Subsolid Pulmonary Nodules in Different Sizes. *Cancer Manag Res.* 2020;12:8057-8066. doi: 10.2147/CMAR.S256719.
50. Feng B, Chen X, Chen Y, Liu K, Li K, Liu X, *et al*. Radiomics nomogram for preoperative differentiation of lung tuberculoma from adenocarcinoma in solitary pulmonary solid nodule. *Eur J Radiol.* 2020;128:109022. doi: 10.1016/j.ejrad.2020. 109022.
51. Guo HY, Lin JT, Huang HH, Gao Y, Yan MR, Sun M, *et al*. Development and Validation of a 18F-FDG PET/CT-Based Clinical Prediction Model for Estimating Malignancy in Solid Pulmonary Nodules Based on a Population With High Prevalence of Malignancy. *Clin Lung Cancer.* 2020;21(1):47-55. doi: 10.1016/j.cllc. 2019.07.014.
52. Erdoğu V, Çitak N, Yerlioğlu A, Aksoy Y, Emetli Y, Pekçolaklar A, *et al*. Is the Yedikule-solitary pulmonary nodule malignancy risk score sufficient to predict malignancy? An internal validation study. *Interact Cardiovasc Thorac Surg.* 2021; 33(2):258-265. doi: 10.1093/icvts/ivab083.
53. Hou H, Yu S, Xu Z, Zhang H, Liu J, Zhang W. Prediction of malignancy for solitary pulmonary nodules based on imaging, clinical characteristics and tumor marker levels. *Eur J Cancer Prev.* 2021;30(5):382-388. doi: 10.1097/CEJ. 0000000000000637.
54. Wu Z, Huang T, Zhang S, Cheng D, Li W, Chen B. A prediction model to evaluate the pretest risk of malignancy in solitary pulmonary nodules: evidence from a large Chinese southwestern population. *J Cancer Res Clin Oncol.* 2021;147(1): 275-285. doi: 10.1007/s00432-020-03408-2.
55. Zhao HC, Xu QS, Shi YB, Ma XJ. Clinical-radiological predictive model in differential diagnosis of small (≤ 20 mm) solitary pulmonary nodules. *BMC Pulm Med.* 2021;21(1):281. doi: 10.1186/s12890-021-01651-y.
56. Lin RY, Lv FJ, Fu BJ, Li WJ, Liang ZR, Chu ZG. Features for Predicting Absorbable Pulmonary Solid Nodules as Depicted on Thin-Section Computed Tomography. *J Inflamm Res.* 2021;14:2933-2939. doi: 10.2147/JIR.S318125.
57. Tang J, Liu C, Wang P, Cui Y. [Multivariate Analysis of Solid Pulmonary Nodules Smaller than 1 cm in Distinguishing Lung Cancer from Intrapulmonary Lymph Nodes]. *Zhongguo Fei Ai Za Zhi.* 2021;24(2):94-98. Chinese. doi: 10.3779/j. issn.1009-3419.2021.102.05.
58. Zhuo Y, Zhan Y, Zhang Z, Shan F, Shen J, Wang D, *et al*. Clinical and CT radiomics nomogram for preoperative differentiation of pulmonary adenocarcinoma from tuberculoma in solitary solid nodule. *Front Oncol.* 2021;11: 701598. doi: 10.3389/fonc.2021.701598.
59. Jacob M, Romano J, Ara Jo D, Pereira JM, Ramos I, Hespanhol V. Predicting lung nodules malignancy. *Pulmonology.* 2022;28(6):454-460. doi: 10.1016/j.pulmoe. 2020.06.011.
60. Yi L, Peng Z, Chen Z, Tao Y, Lin Z, He A, *et al*. Identification of pulmonary adenocarcinoma and benign lesions in isolated solid lung nodules based on a nomogram of intranodal and perinodal CT radiomic features. *Front Oncol.* 2022;12: 924055. doi: 10.3389/fonc.2022.924055.
61. He C, Liu J, Li Y, Lin L, Qing H, Guo L, *et al*. Quantitative parameters of enhanced dual-energy computed tomography for differentiating lung cancers from benign lesions in solid pulmonary nodules. *Front Oncol.* 2022;12:1027985. doi: 10. 3389/fonc.2022.1027985.
62. Chen C, Geng Q, Song G, Zhang Q, Wang Y, Sun D, *et al*. A comprehensive nomogram combining CT-based radiomics with clinical features for differentiation of benign and malignant lung subcentimeter solid nodules. *Front Oncol.* 2023;13: 1066360. doi: 10.3389/fonc.2023.1066360.
63. Xie X, Liu K, Luo K, Xu Y, Zhang L, Wang M, *et al*. Value of dual-layer spectral detector computed tomography in the diagnosis of benign/malignant solid solitary pulmonary nodules and establishment of a prediction model. *Front Oncol.* 2023;13:1147479. doi: 10.3389/fonc.2023.1147479.
64. Jiang J, Lv FJ, Tao Y, Fu BJ, Li WJ, Lin RY, *et al*. Differentiation of pulmonary solid nodules attached to the pleura detected by thin-section CT. *Insights Imaging.* 2023;14(1):146. doi: 10.1186/s13244-023-01504-8.
65. Zhang CR, Wang Q, Feng H, Cui YZ, Yu XB, Shi GF. Computed- tomography-based radiomic nomogram for predicting the risk of indeterminate small (5-20 mm) solid pulmonary nodules. *Diagn Interv Radiol.* 2023; 29(2):283-290. doi: 10.4274/dir.2022.22395.
66. Li X, Jin F, Zhou T, Ru Y, Gu F, Wang L, *et al*. Diagnostic Accuracy of CT-Guided Percutaneous Pulmonary Biopsy for Distinguishing Benign and Malignant Solitary Pulmonary Nodules. *Altern Ther Health Med.* 2023;29(8):918-923.
67. Liu J, Qi L, Wang Y, Li F, Chen J, Cui S, *et al*. Development of a combined radiomics and CT feature-based model for differentiating malignant from benign subcentimeter solid pulmonary nodules. *Eur Radiol Exp.* 2024;8(1):8. doi: 10.1186/ s41747-023-00400-6.
68. He XQ, Huang XT, Luo TY, Liu X, Li Q. The differential computed tomography features between small benign and malignant solid solitary pulmonary nodules with different sizes. *Quant Imaging Med Surg.* 2024;14(2):1348-1358. doi: 10.21037/ qims-23-995.
69. Qu BQ, Wang Y, Pan YP, Cao PW, Deng XY. The scoring system combined with radiomics and imaging features in predicting the malignant potential of incidental indeterminate small (<20 mm) solid pulmonary nodules. *BMC Med Imaging.* 2024;24(1):234. doi: 10.1186/s12880-024-01413-2.
70. Wang W, Li M, Zhang Q. Decreased levels of sex hormones in females with solitary pulmonary nodules were risk factors for malignancy. *J Cardiothorac Surg.* 2024;19(1):119. doi: 10.1186/s13019-024-02609-x.
71. Zheng J, Hao Y, Guo Y, Du M, Wang P, Xin J. An 18F-FDG-PET/CT-based radiomics signature for estimating malignance probability of solitary pulmonary nodule. *Clin Respir J.* 2024;18(5):e13751. doi: 10.1111/crj.13751.
72. Apostolopoulos ID, Papathanasiou ND, Apostolopoulos DJ, Papandrianos N, Papageorgiou EI. Integrating Machine Learning in Clinical Practice for Characterizing the Malignancy of Solitary Pulmonary Nodules in PET/CT Screening. *Diseases.* 2024;12(6):115. doi: 10.3390/diseases12060115.
73. Chen H, Wen Y, Wu W, Zhang Y, Pan X, Guan Y, *et al*. Prediction of Malignancy and Pathological Types of Solid Lung Nodules on CT Scans Using a Volumetric SWIN Transformer. *J Imaging Inform Med.* 2025;38(3):1509-1517. doi: 10.1007/s10278-024-01090-1.
74. Zhao T, Yue Y, Sun H, Li J, Wen Y, Yao Y, *et al*. MAEMC-NET: a hybrid self-supervised learning method for predicting the malignancy of solitary pulmonary nodules from CT images. *Front Med (Lausanne).* 2025;12:1507258. doi: 10.3389/ fmed.2025.1507258.
75. Baum P, Schlamp K, Klotz LV, Eichhorn ME, Herth F, Winter H. Incidental Pulmonary Nodules: Differential Diagnosis and Clinical Management. *Dtsch Arztebl Int.* 2024;121(25):853-860. doi: 10.3238/arztebl.m2024.0177.
76. Furman AM, Dit Yafawi JZ, Soubani AO. An update on the evaluation and management of small pulmonary nodules. *Future Oncol.* 2013;9(6):855-65. doi: 10. 2217/fon.13.17.
77. Kowalewski J. Guzek subcentymetrowy płuca--problemy diagnostyczne i lecznicze [Subcentimeter pulmonary nodule: diagnostic and therapeutic problems]. *Pol Merkur Lekarski.* 2008;25(148):368-73. Polish.
78. Mery CM, Pappas AN, Bueno R, Mentzer SJ, Lukanich JM, Sugarbaker DJ, *et al*. Relationship between a history of antecedent cancer and the probability of malignancy for a solitary pulmonary nodule. *Chest.* 2004;125(6):2175-81. doi: 10. 1378/chest.125.6.2175.
79. Mets OM, de Jong PA, Scholten ET, Chung K, van Ginneken B, Schaefer-Prokop CM. Subsolid pulmonary nodule morphology and associated patient characteristics in a routine clinical population. *Eur Radiol.* 2017;27(2):689-696. doi: 10.1007/ s00330-016-4429-9.
80. Zhu X, Shen C, Dong J. A clinically applicable model more suitable for predicting malignancy or benignity of pulmonary ground glass nodules in women patients. *BMC Cancer.* 2024;24(1):1225. doi: 10.1186/s12885-024-13004-z.
81. Tang S, Bao Q, Ji Q, Wang T, Wang N, Yang M, *et al*. Improvement of RT-DETR model for ground glass pulmonary nodule detection. *PLoS One.* 2025; 20(3):e0317114. doi: 10.1371/journal.pone.0317114.
82. Wang R, Qi T. Creation of nomograms that combine clinical, CT, and radiographic features to separate benign from malignant diseases using spiculation or (and) lobulation signs. *Curr Probl Diagn Radiol.* 2025;54(4):443-448. doi: 10.1067/j. cpradiol.2024.12.014.
83. Ahn S, Moon Y. Uniportal video-assisted thoracoscopic fissureless right upper lobe anterior segmentectomy for inflammatory myofibroblastic tumor: A case report. *World J Clin Cases.* 2024;12(2):425-430. doi: 10.12998/wjcc.v12.i2.425.
84. Lin H, Huang C, Wang W, Luo J, Yang X, Liu Y. Measuring Interobserver Disagreement in Rating Diagnostic Characteristics of Pulmonary Nodule Using the Lung Imaging Database Consortium and Image Database Resource Initiative. *Acad Radiol.* 2017;24(4):401-410. doi: 10.1016/j.acra.2016.11.022.

**Figure legends:**

Figure 1. The PRISMA flowchart regarding the literature search and study selection.

Figure 2. The forest plot for the incidence of malignancy in patients with incidental solitary pulmonary nodules.

Figure 3. Funnel plot for the assessment of publication bias in the meta-analysis of malignancy incidence. A: Funnel plot of the 54 included studies in the primary analysis; B: Funnel plot following the Trim-and-Fill adjustment. The minimal shift from the original estimate (56.7%) to the adjusted estimate (54.7%) indicates robustness of the finding.
