# Supplementary material for: Incidence and risk factors for malignancy in patients with incidental solitary pulmonary nodules: a systematic review and meta-analysis
Source: Ann Med. 2026 Feb 5;58(1):2596547. doi: 10.1080/07853890.2025.2596547 (PMC12879503; doi:10.1080/07853890.2025.2596547)
Supplement: Supplementary figures.docx [file IANN_A_2596547_SM9103.docx]

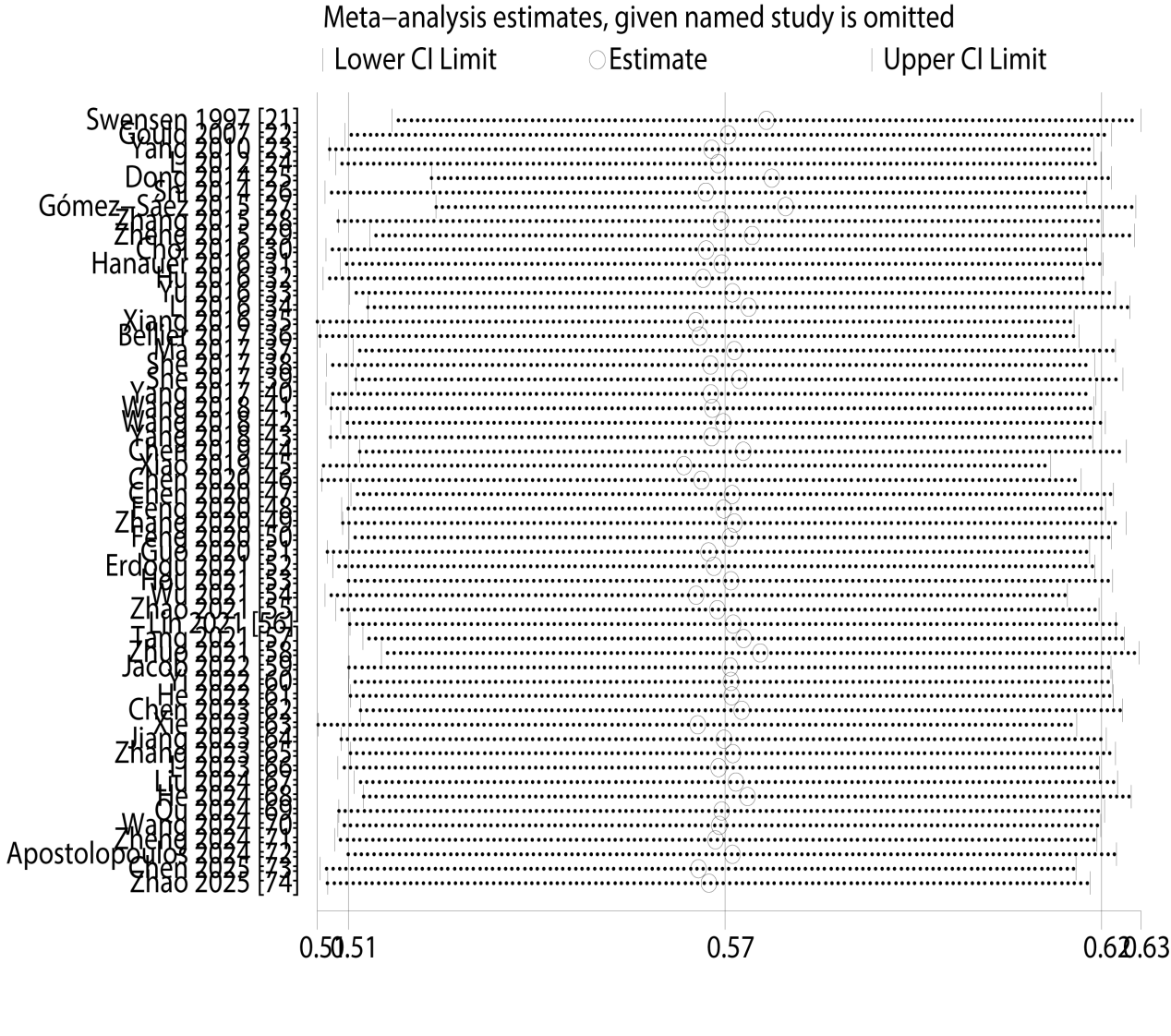


Figure S1. Sensitivity analysis for the incidence of malignancy in patients with incidental SPNs


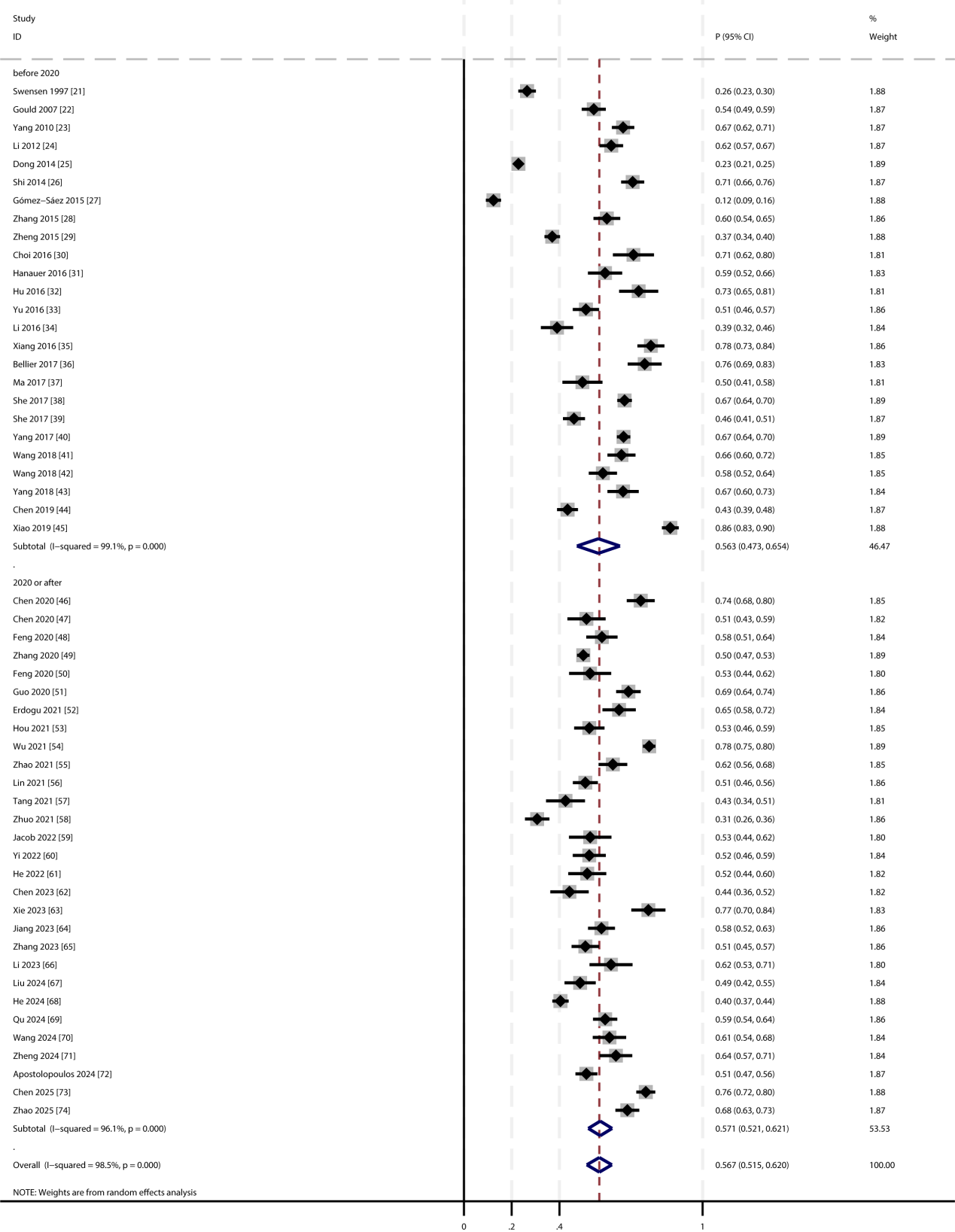


Figure S2. Subgroup analyses for the incidence of malignancy in patients with incidental SPNs based on publication year


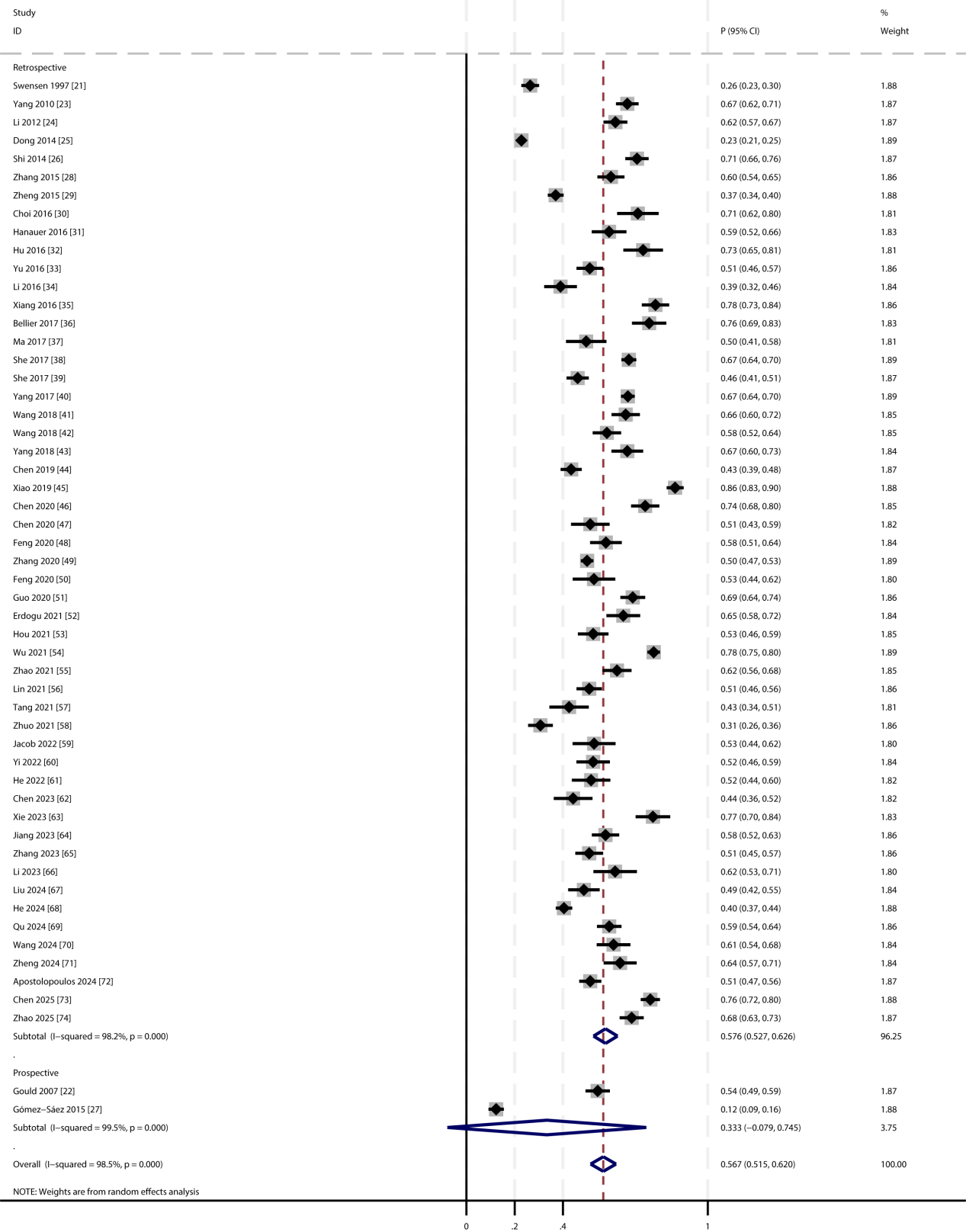


Figure S3. Subgroup analyses for the incidence of malignancy in patients with incidental SPNs based on study design


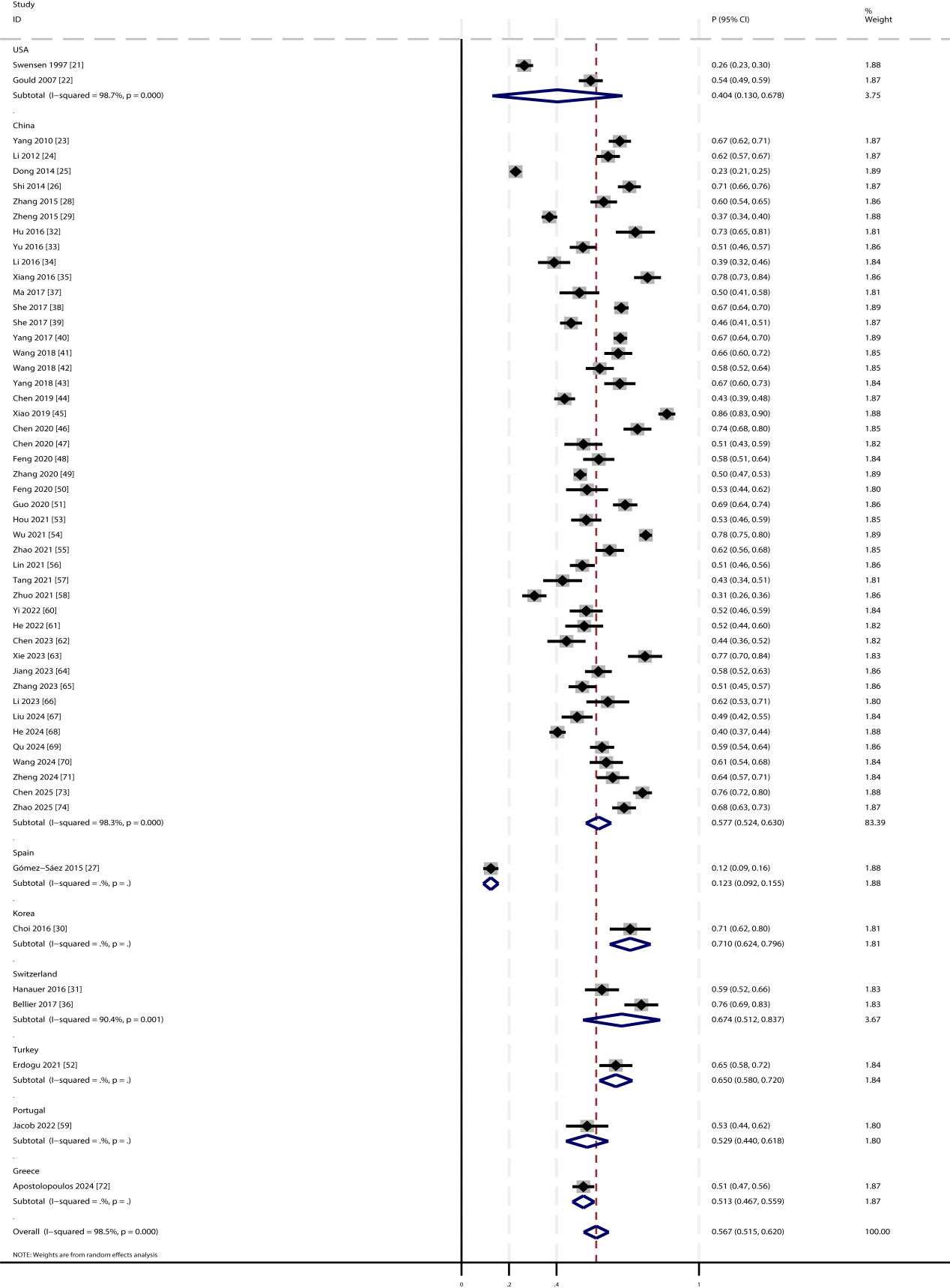


Figure S4. Subgroup analyses for the incidence of malignancy in patients with incidental SPNs based on country


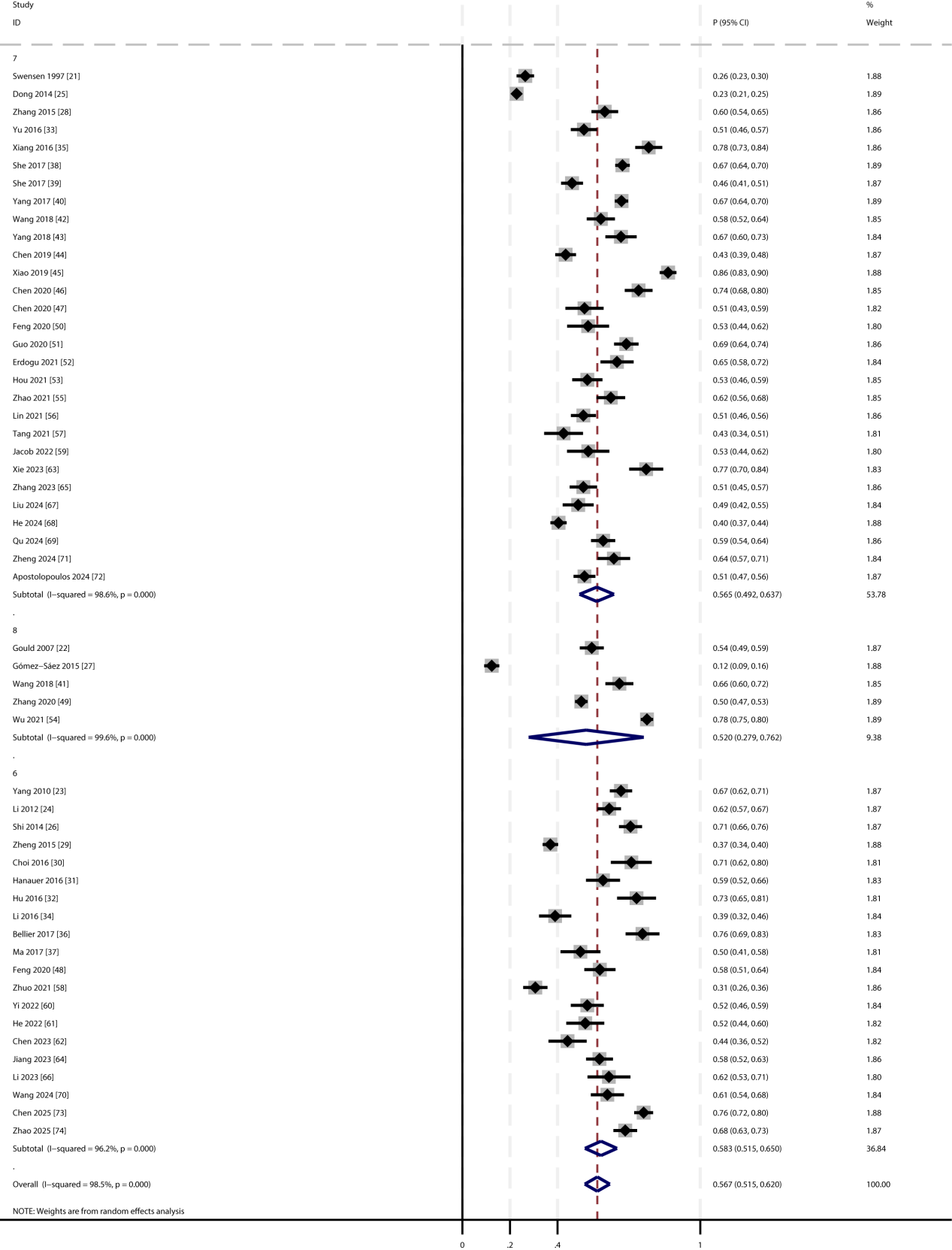


Figure S5. Subgroup analyses for the incidence of malignancy in patients with incidental SPNs based on study quality


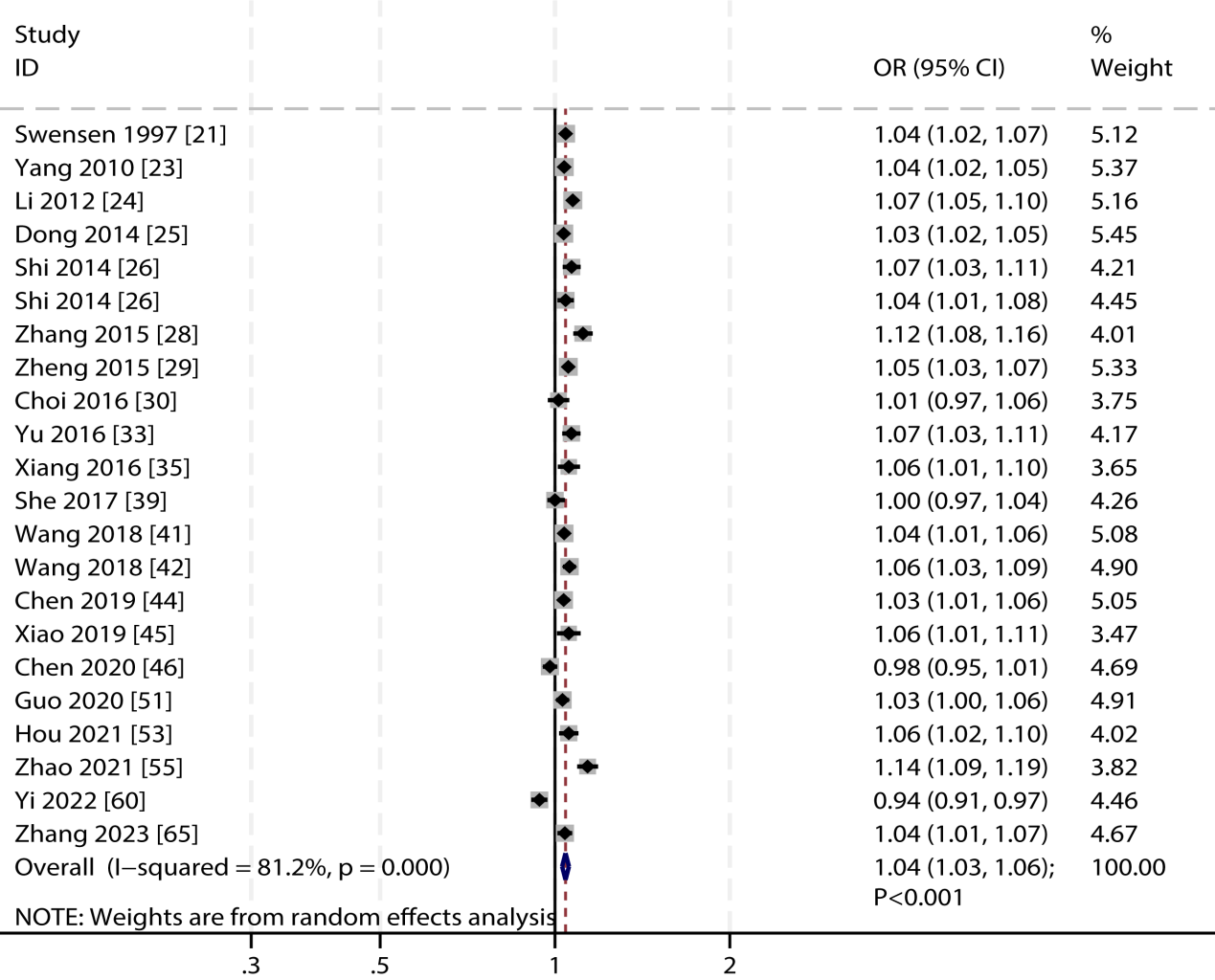


Figure S6. Association of age with the risk of malignancy in patients with incidental SPNs


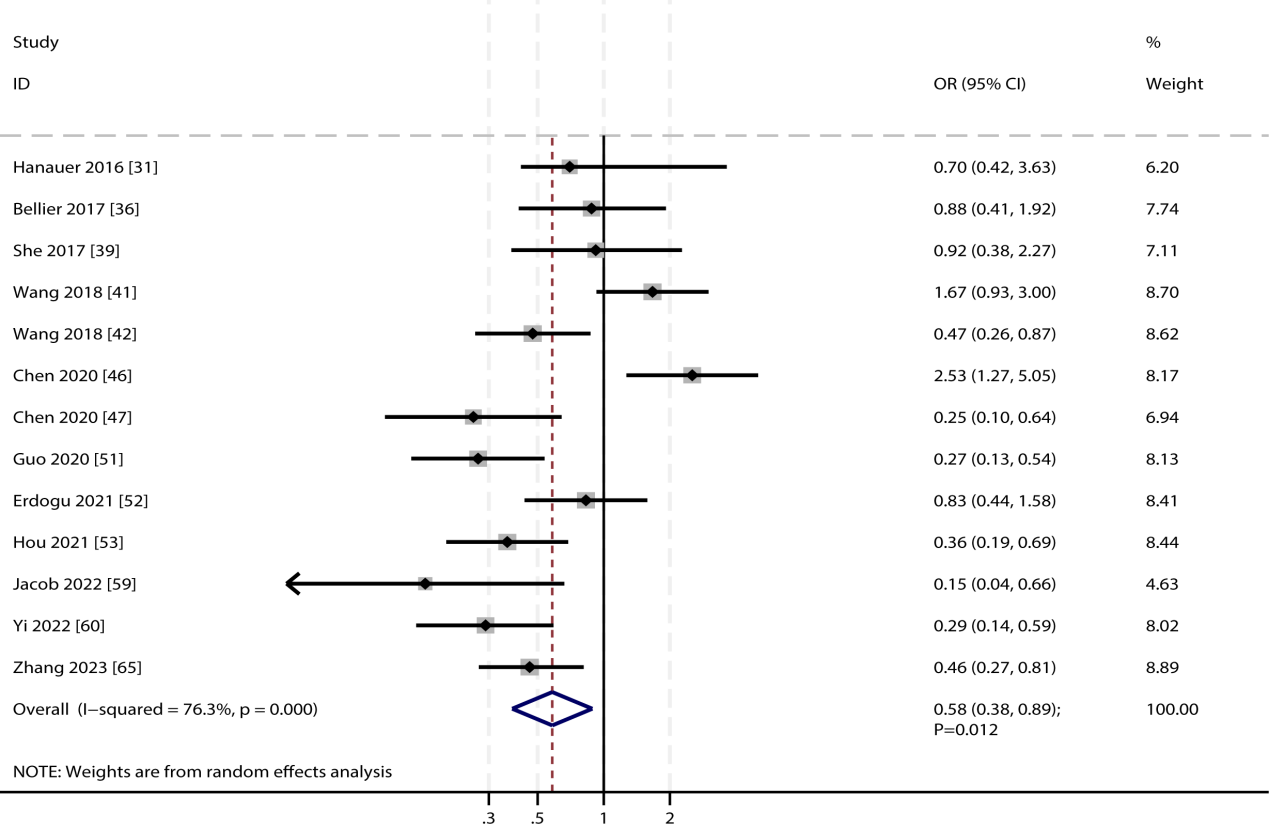


Figure S7. Association of male vs female with the risk of malignancy in patients with incidental SPNs


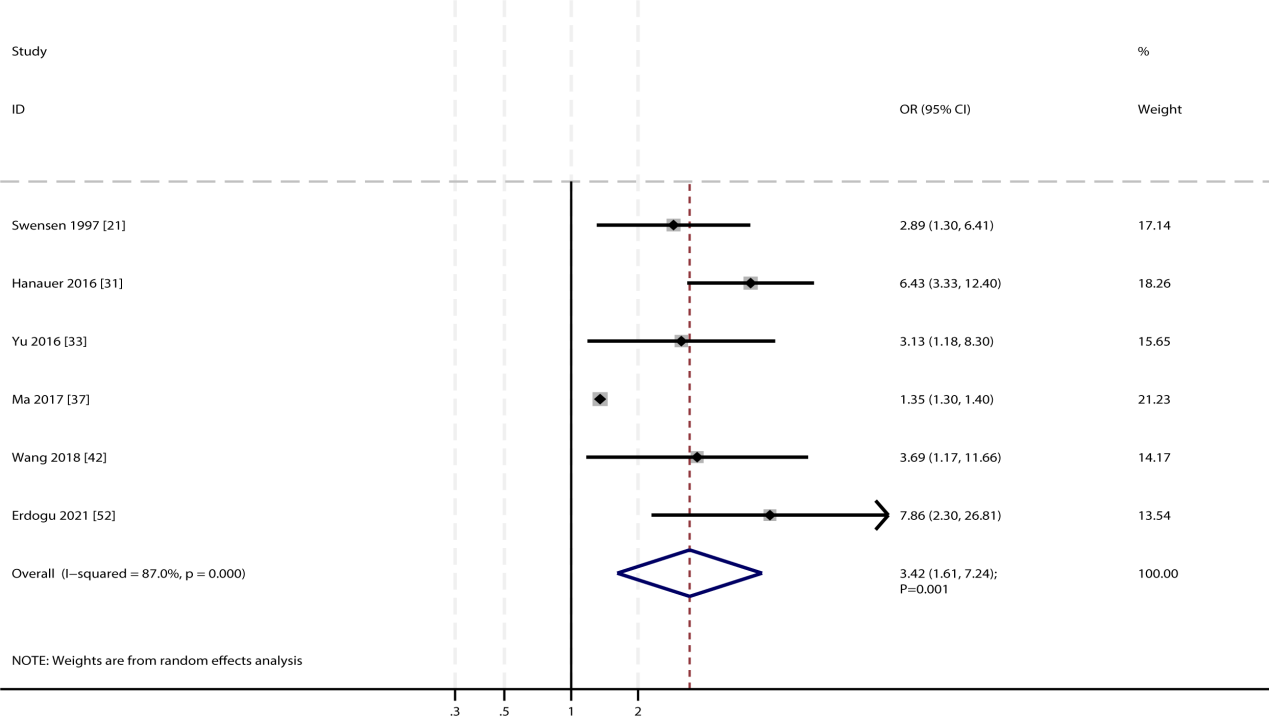


Figure S8. Association of cancer history with the risk of malignancy in patients with incidental SPNs


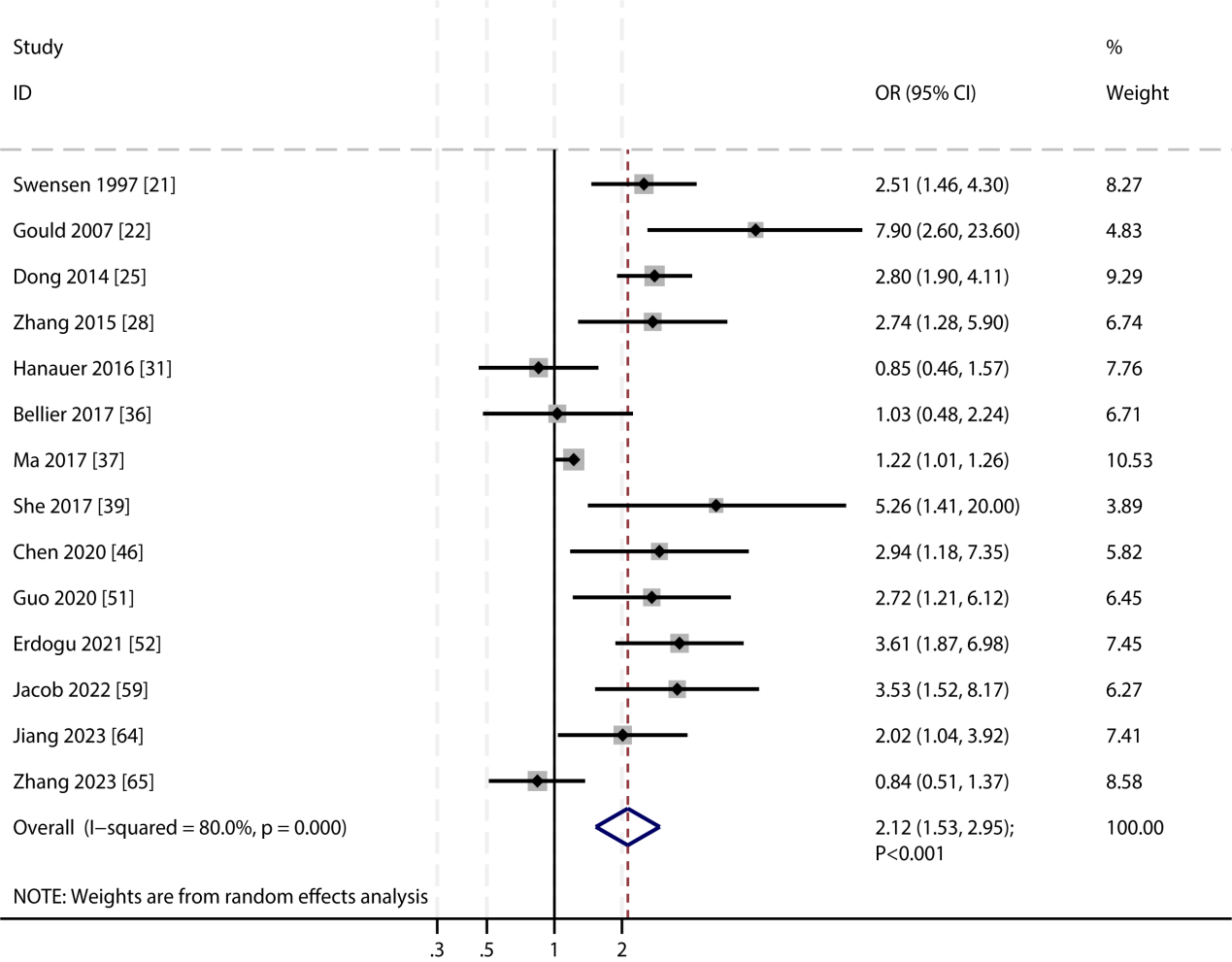


Figure S9. Association of cigarette smoker with the risk of malignancy in patients with incidental SPNs


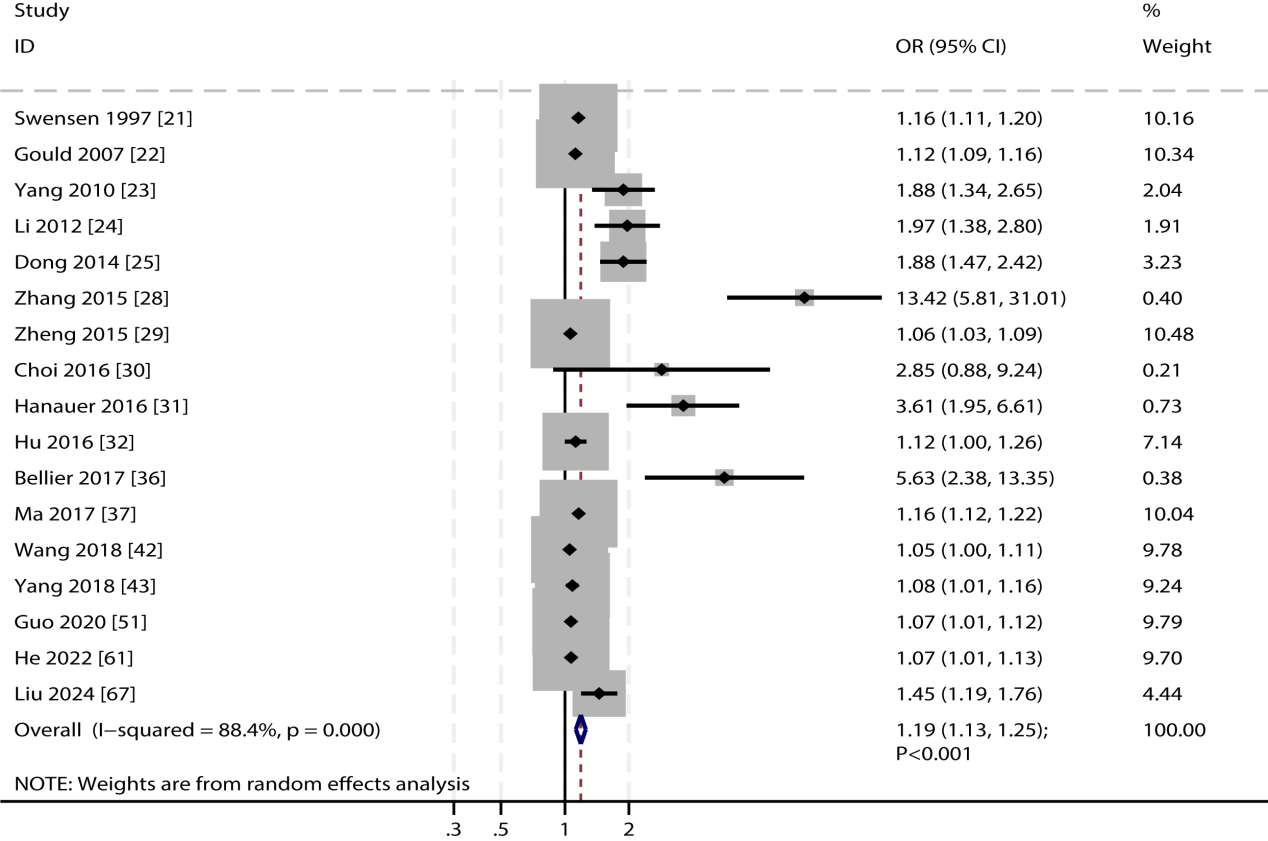


Figure S10. Association of diameter of nodules with the risk of malignancy in patients with incidental SPNs. The visual appearance of this forest plot, particularly the variation in the size of data markers and the width of confidence intervals across studies, is a result of substantial differences in the statistical weight assigned to each study during the meta-analysis. This accurately reflects the underlying data and heterogeneity in study precision, and the formatting is consistent with all other forest plots generated by our statistical software.


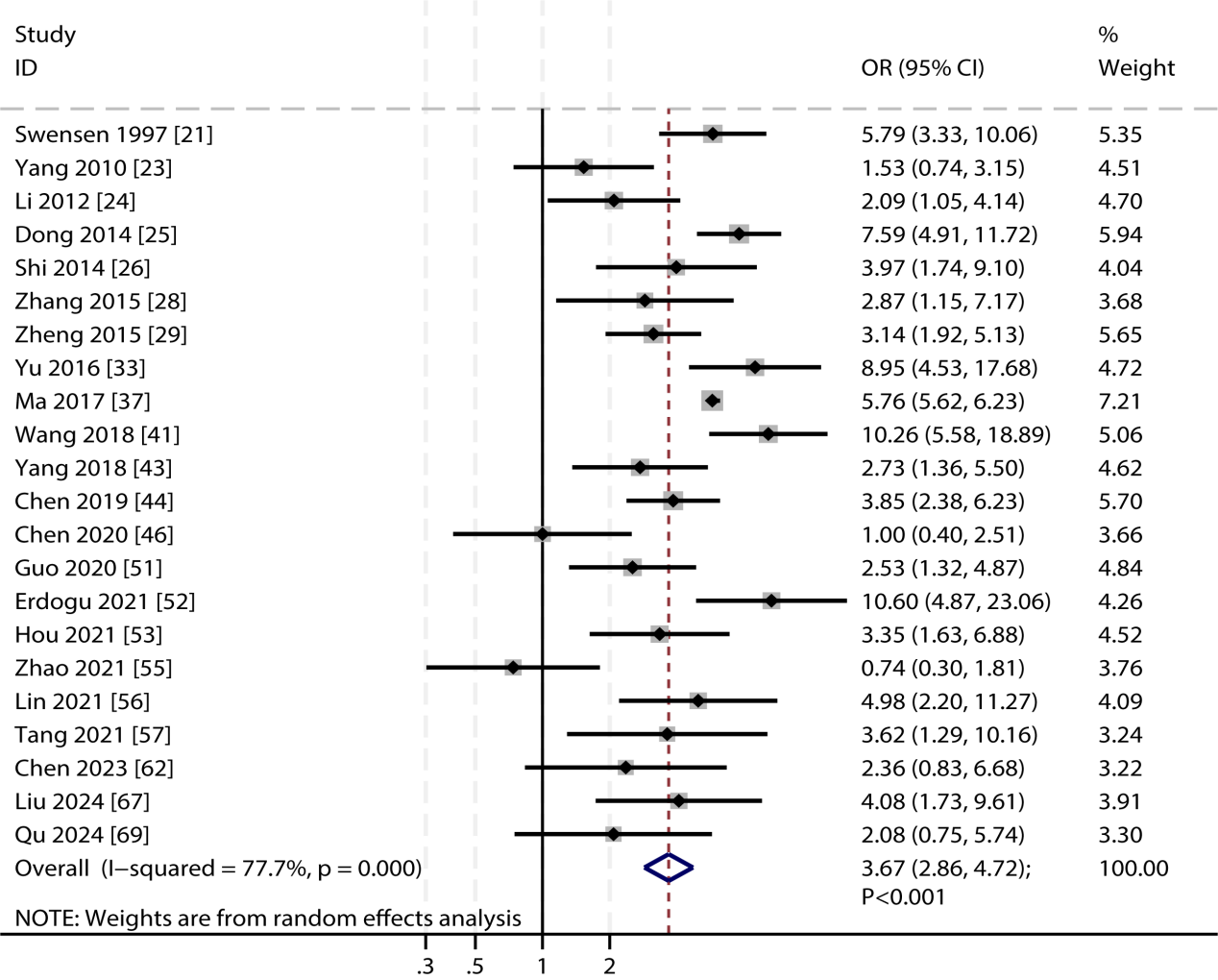


Figure S11. Association of spiculation with the risk of malignancy in patients with incidental SPNs


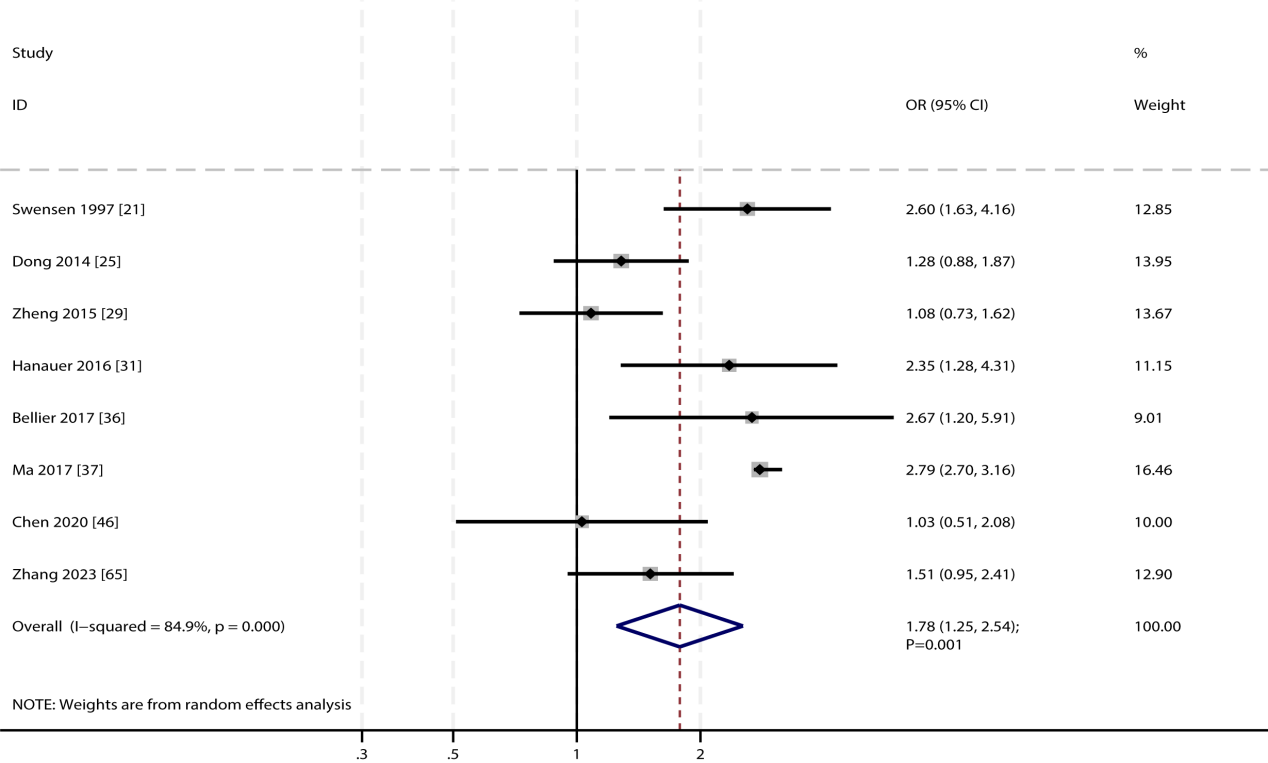


Figure S12. Association of upper lobe with the risk of malignancy in patients with incidental SPNs


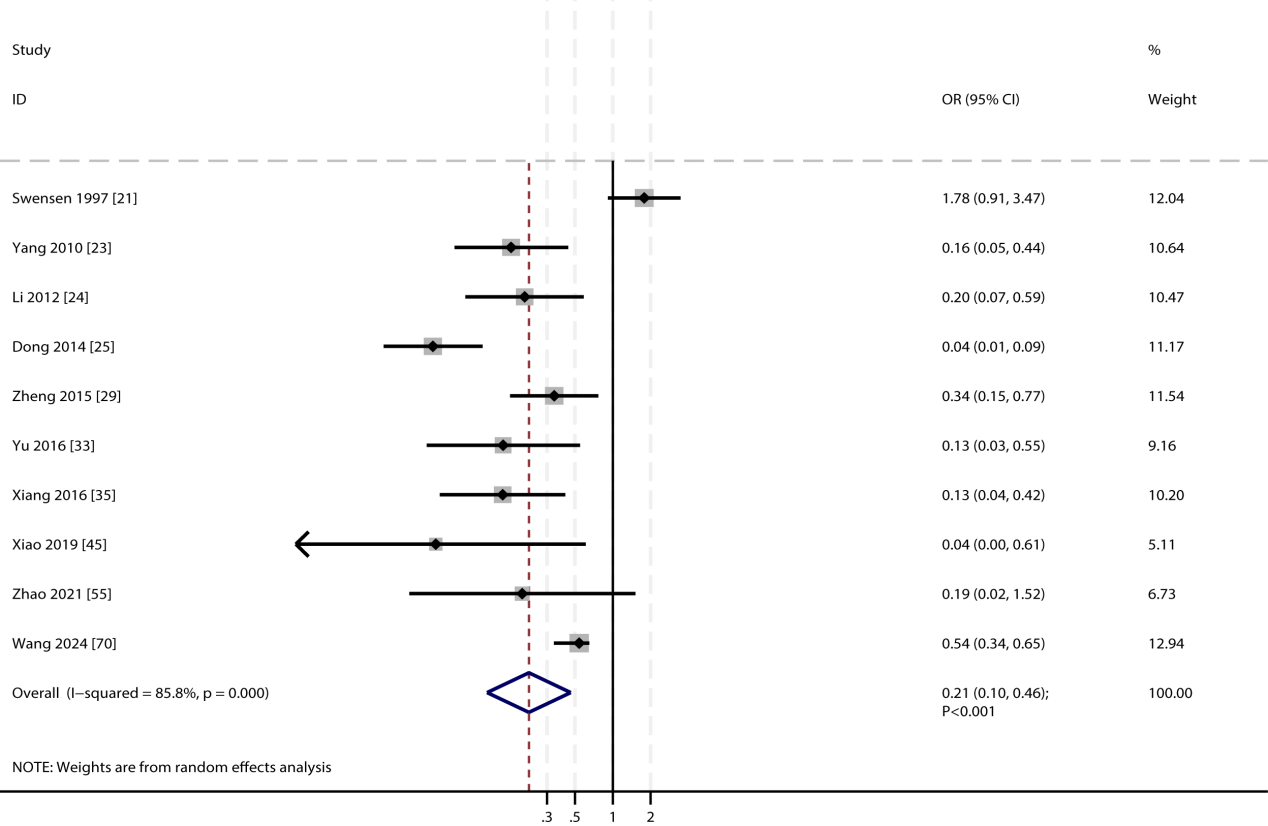


Figure S13. Association of calcification with the risk of malignancy in patients with incidental SPNs


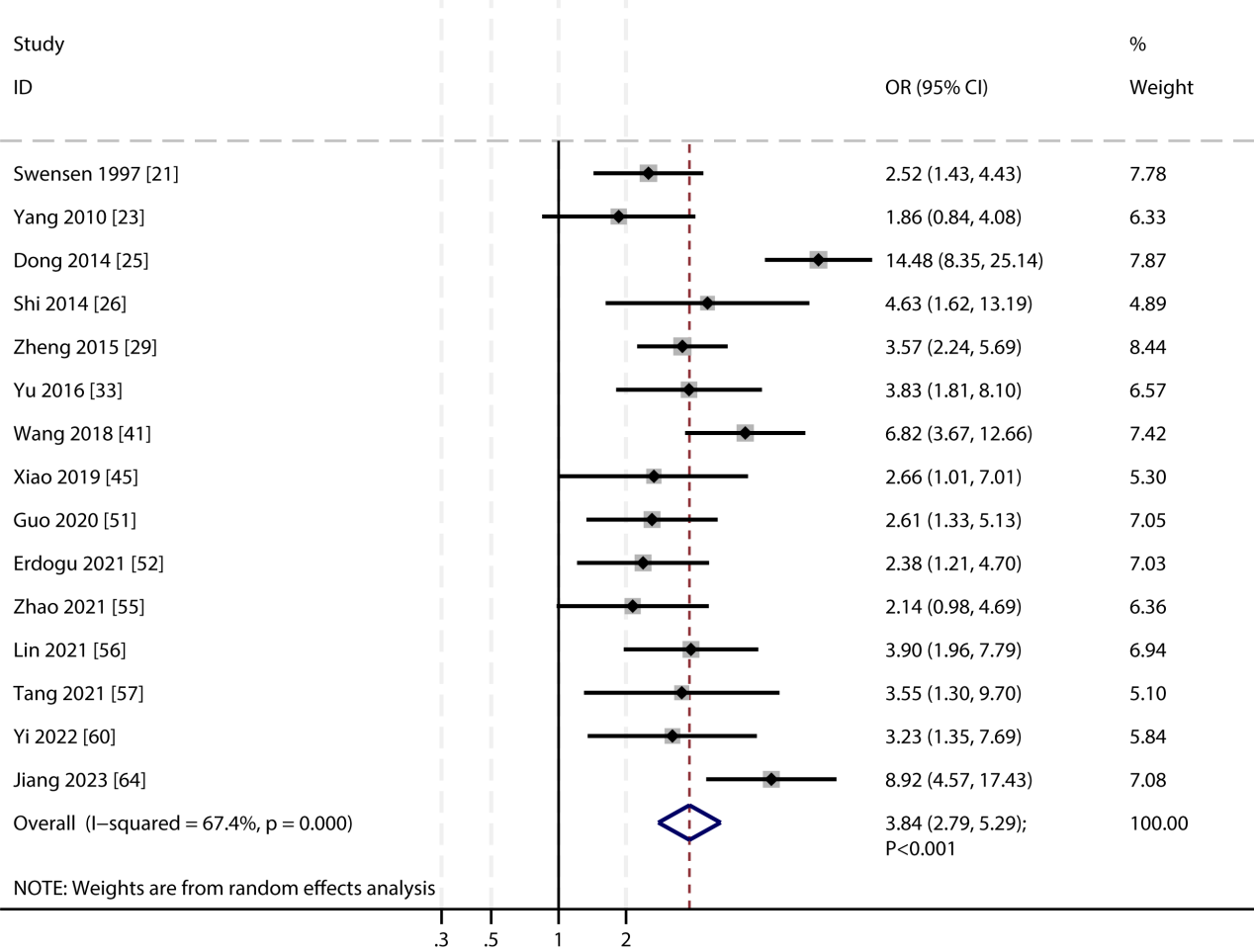


Figure S14. Association of lobulation with the risk of malignancy in patients with incidental SPNs


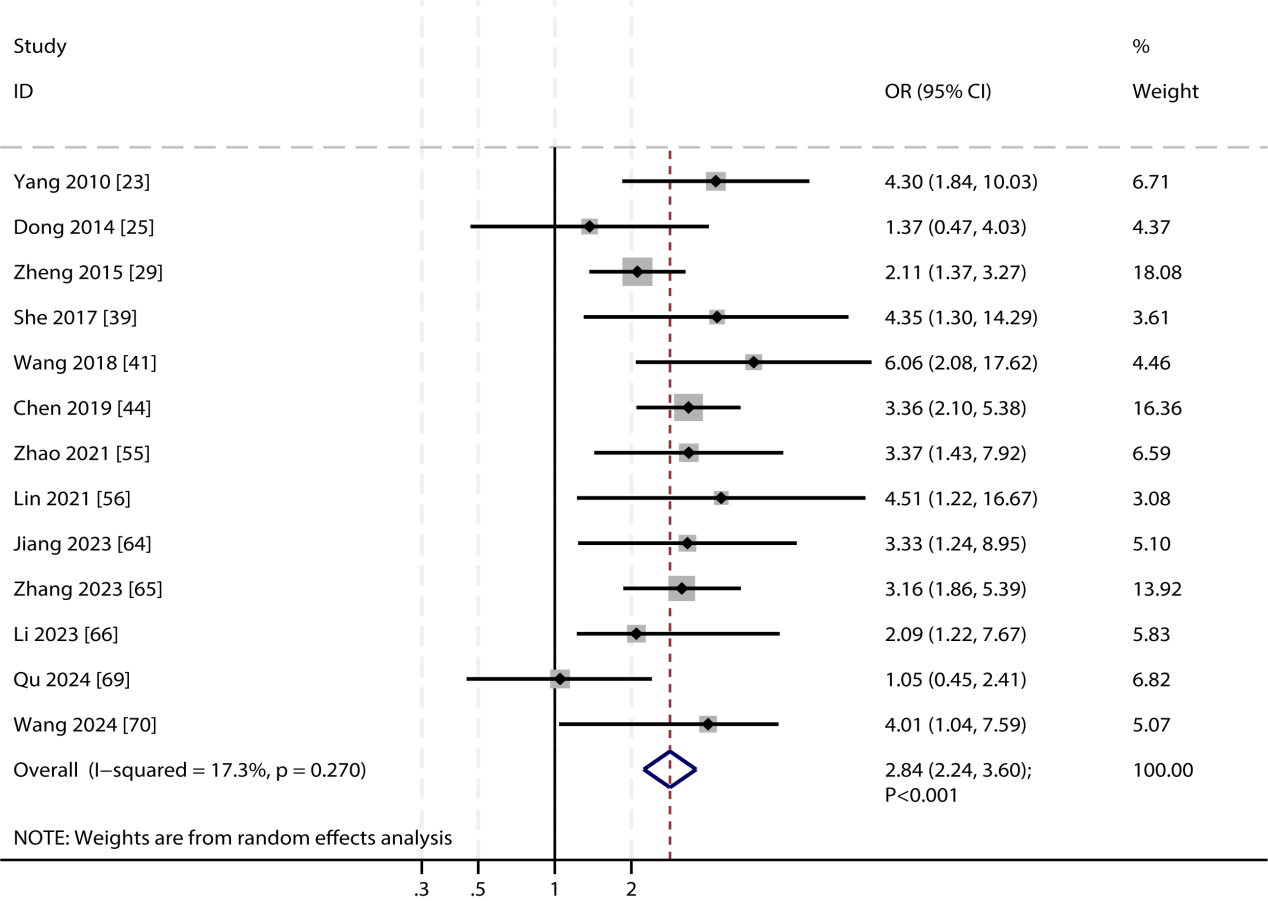


Figure S15. Association of pleural indentation with the risk of malignancy in patients with incidental SPNs


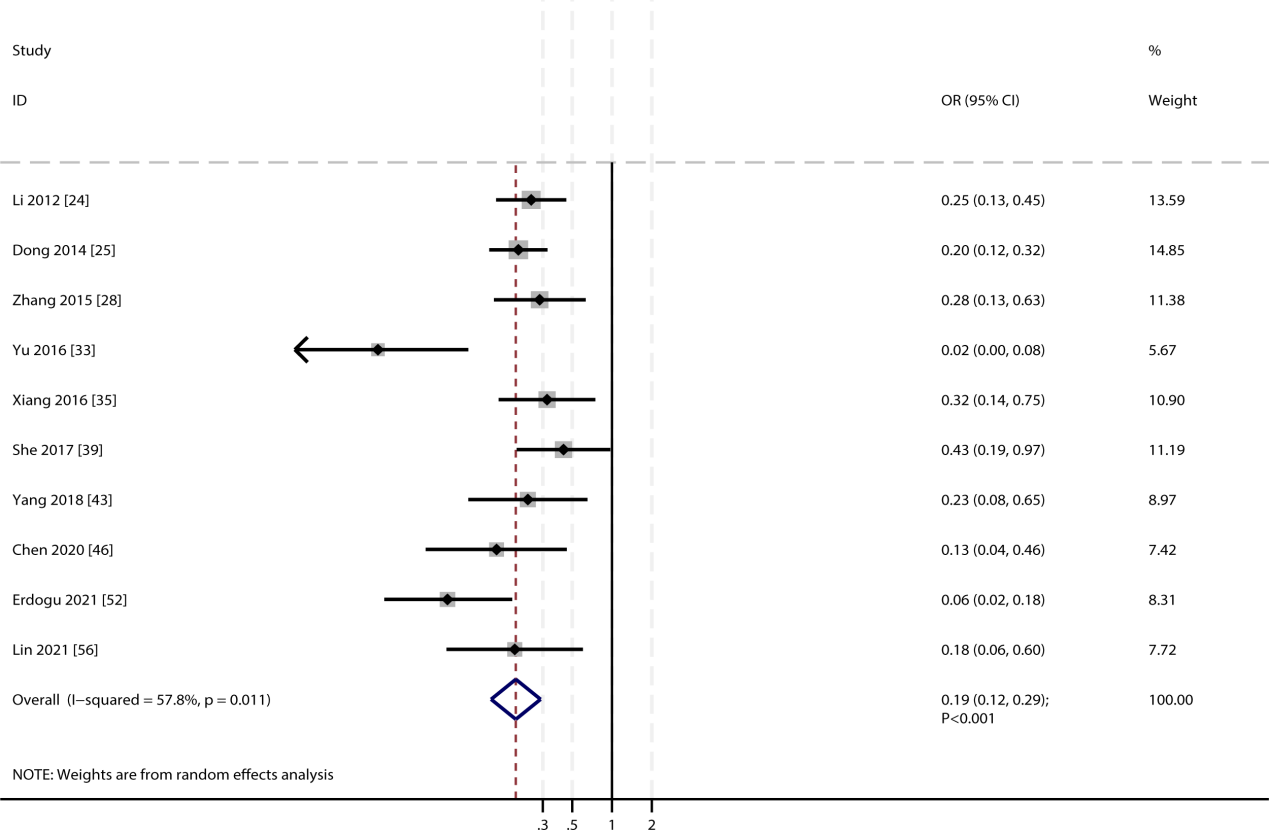


Figure S16. Association of clear border with the risk of malignancy in patients with incidental SPNs


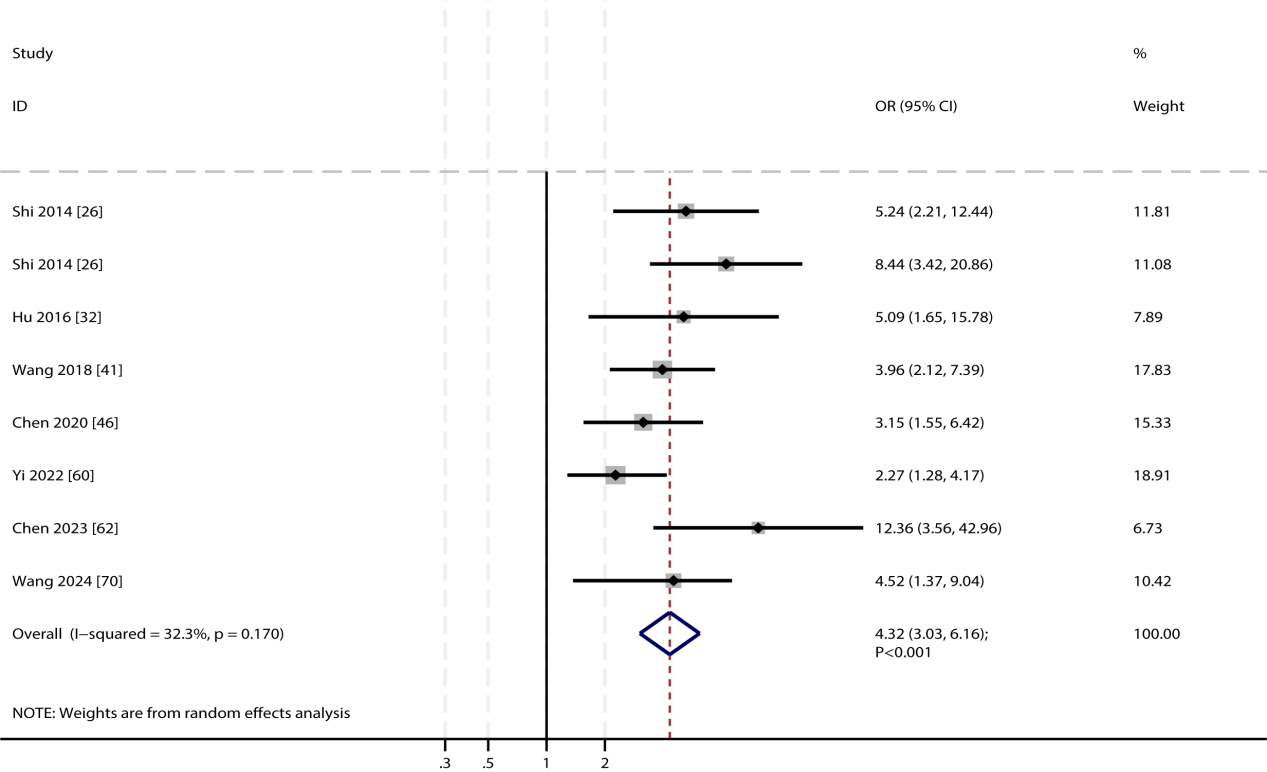


Figure S17. Association of vascular convergence with the risk of malignancy in patients with incidental SPNs


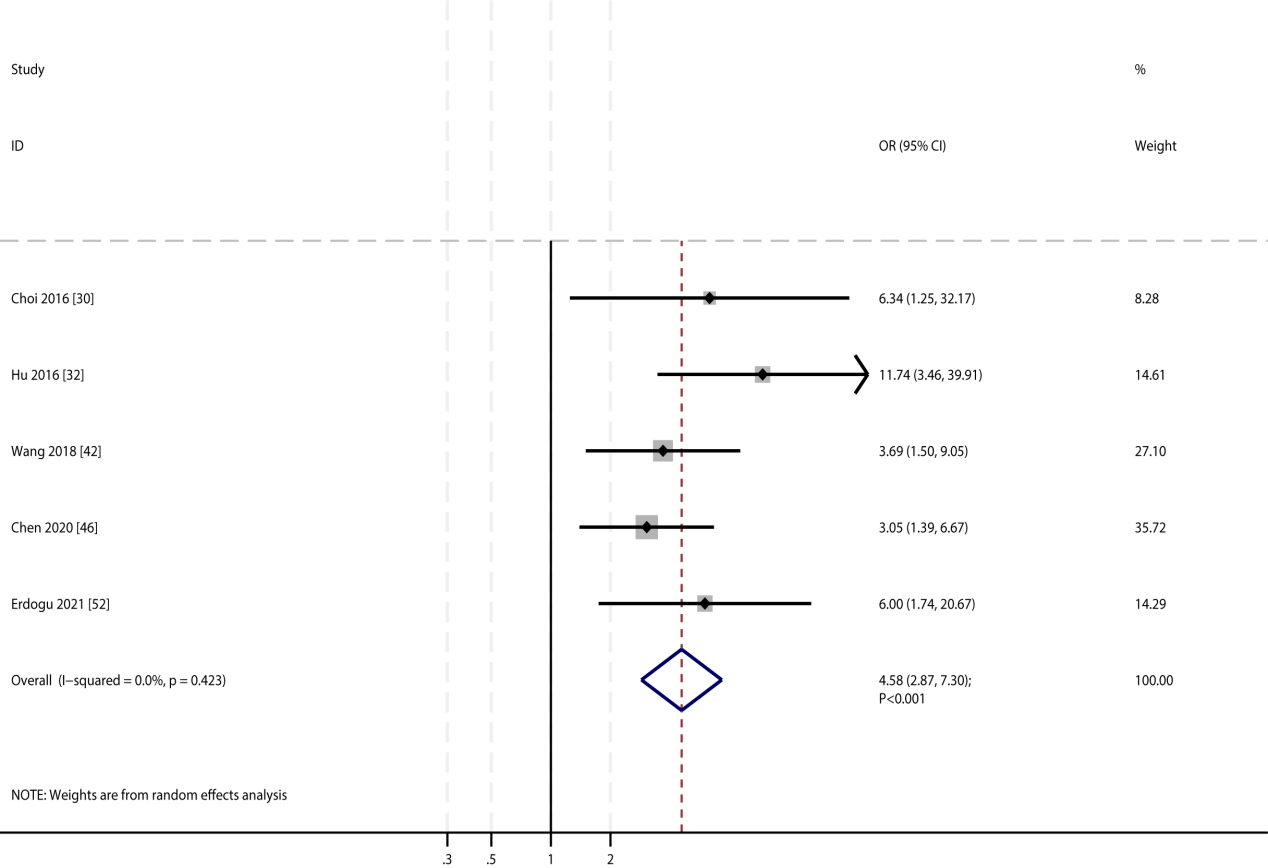


Figure S18. Association of solid nodules with the risk of malignancy in patients with incidental SPNs


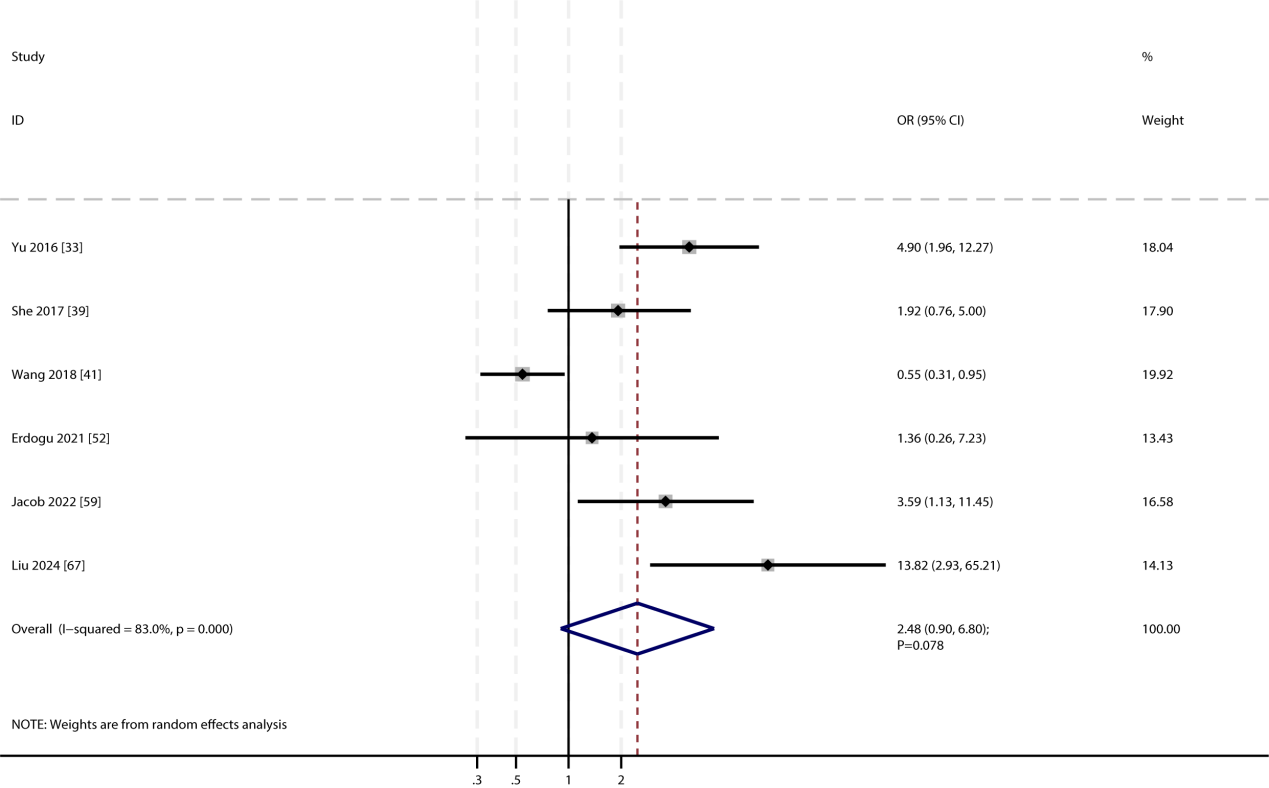


Figure S19. Association of air bronchogram with the risk of malignancy in patients with incidental SPNs


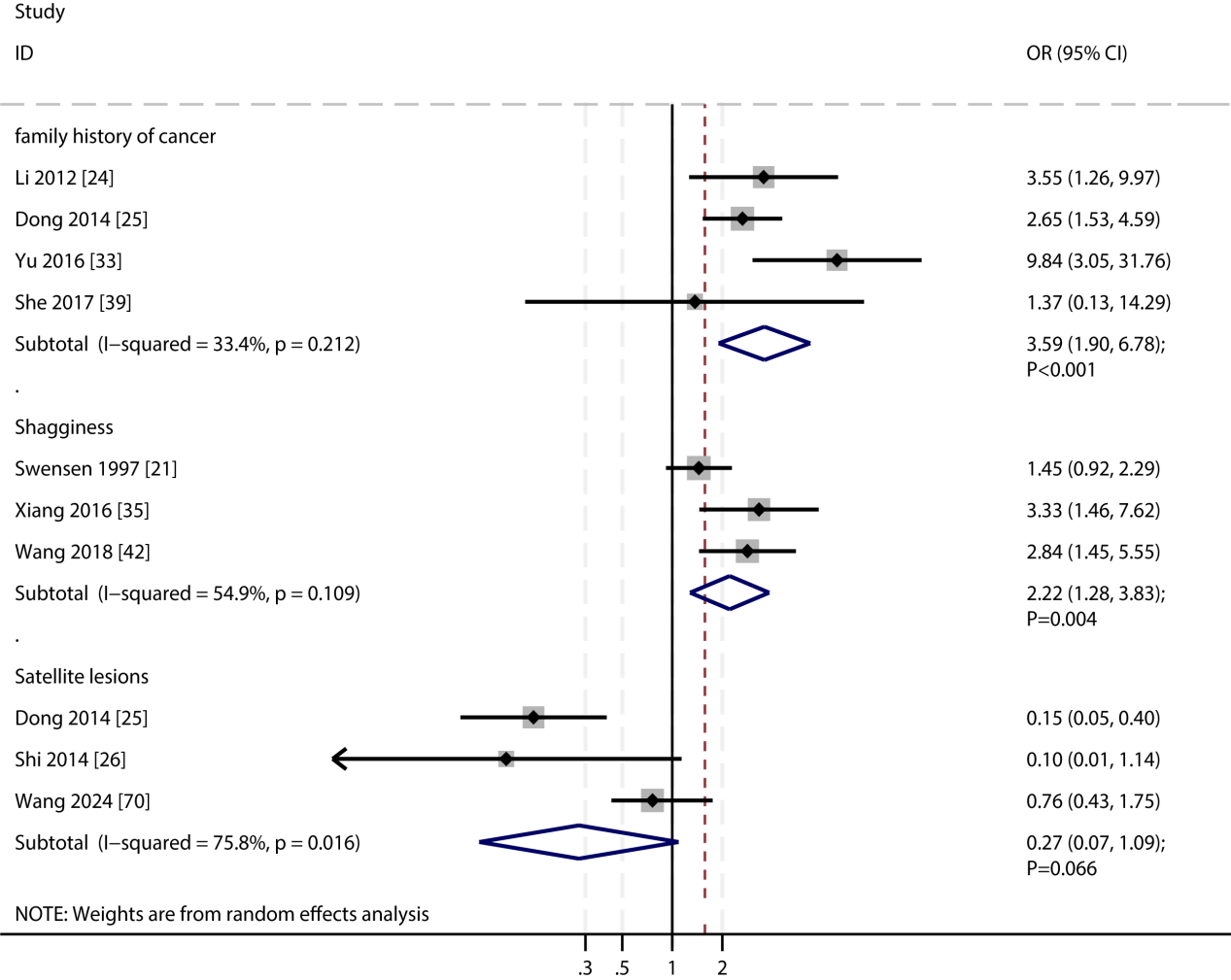


Figure S20. Association of family history of cancer, irregular or ill-defined margins, and satellite lesions with the risk of malignancy in patients with incidental SPNs


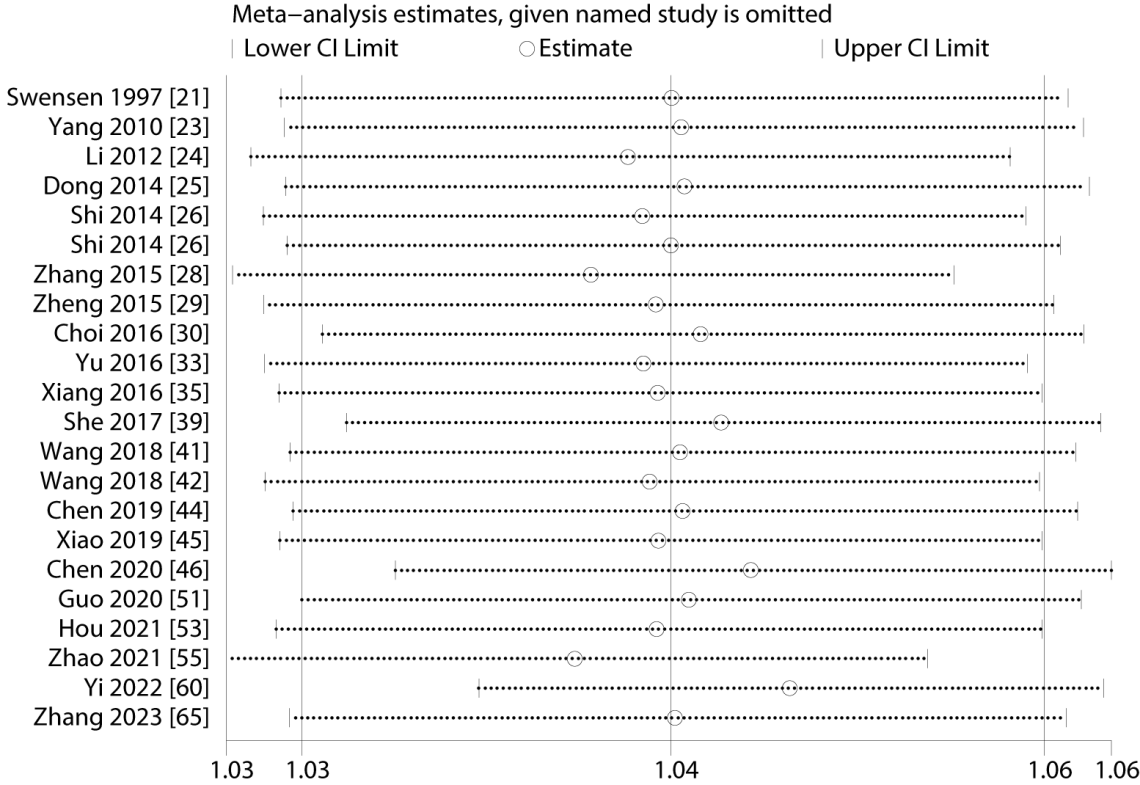


Figure S21. Sensitivity analysis for the association of age with the risk of malignancy in patients with incidental SPNs


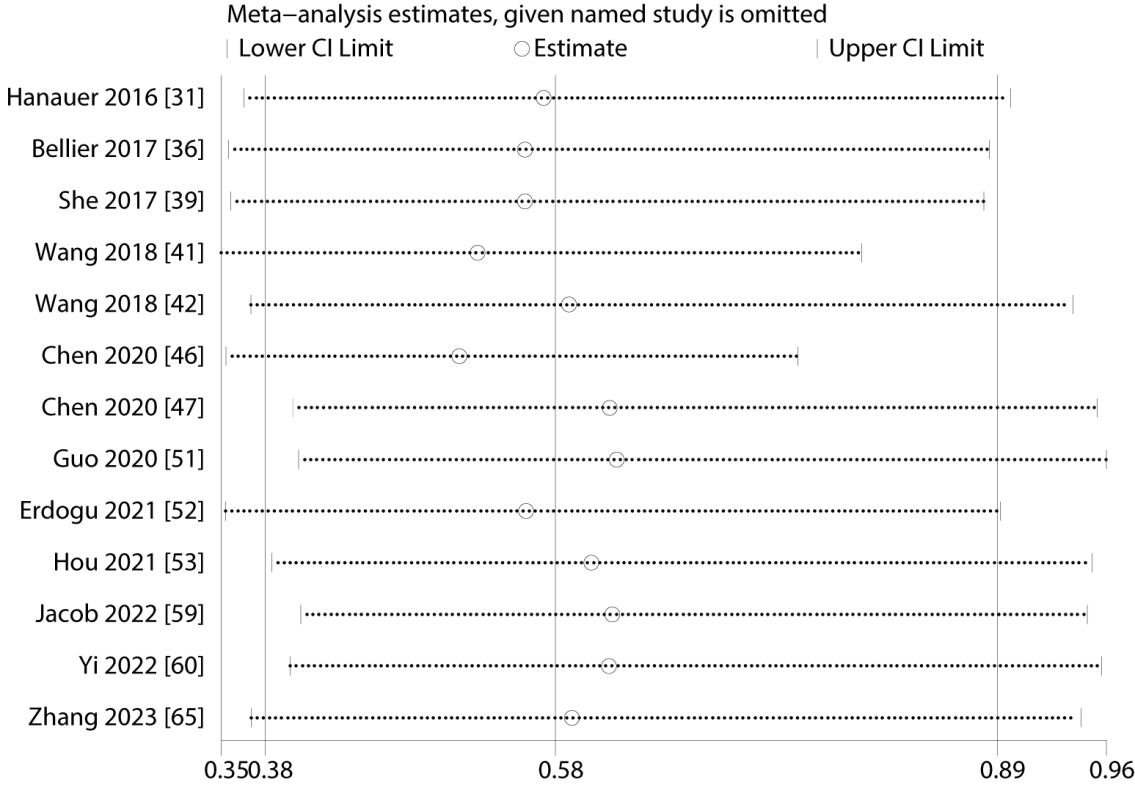


Figure S22. Sensitivity analysis for the association of male vs female with the risk of malignancy in patients with incidental SPNs


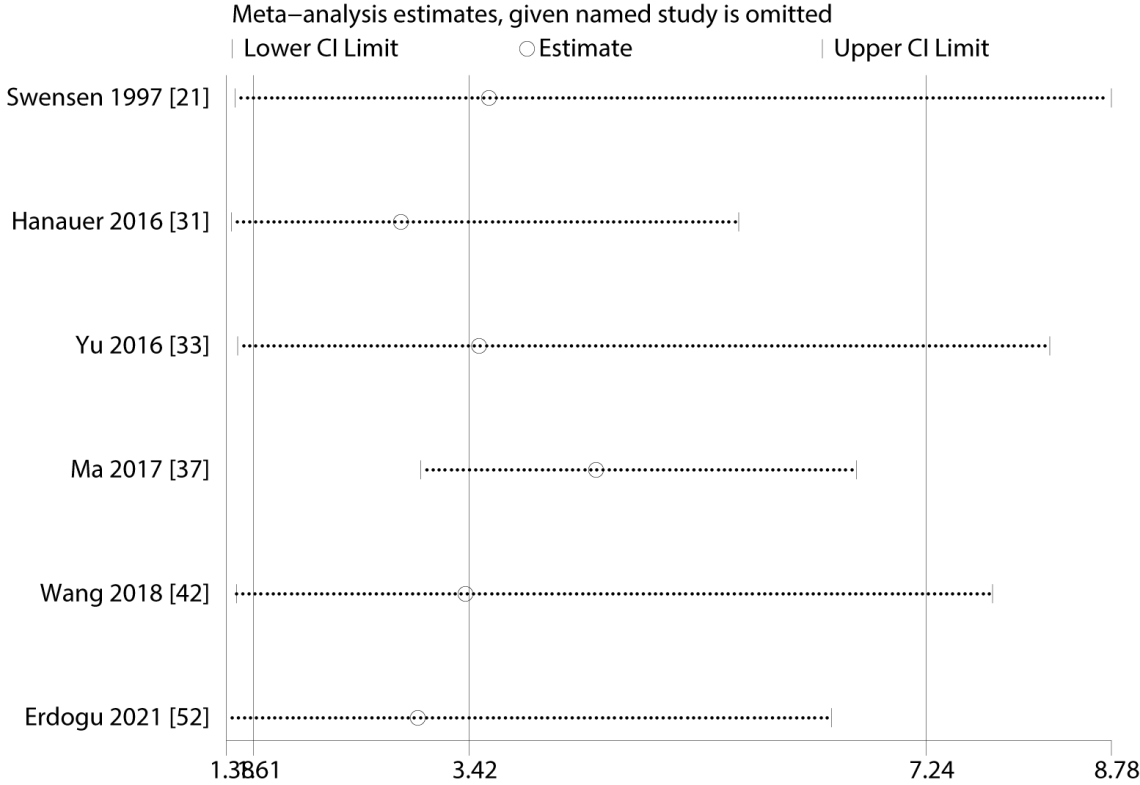


Figure S23. Sensitivity analysis for the association of cancer history with the risk of malignancy in patients with incidental SPNs


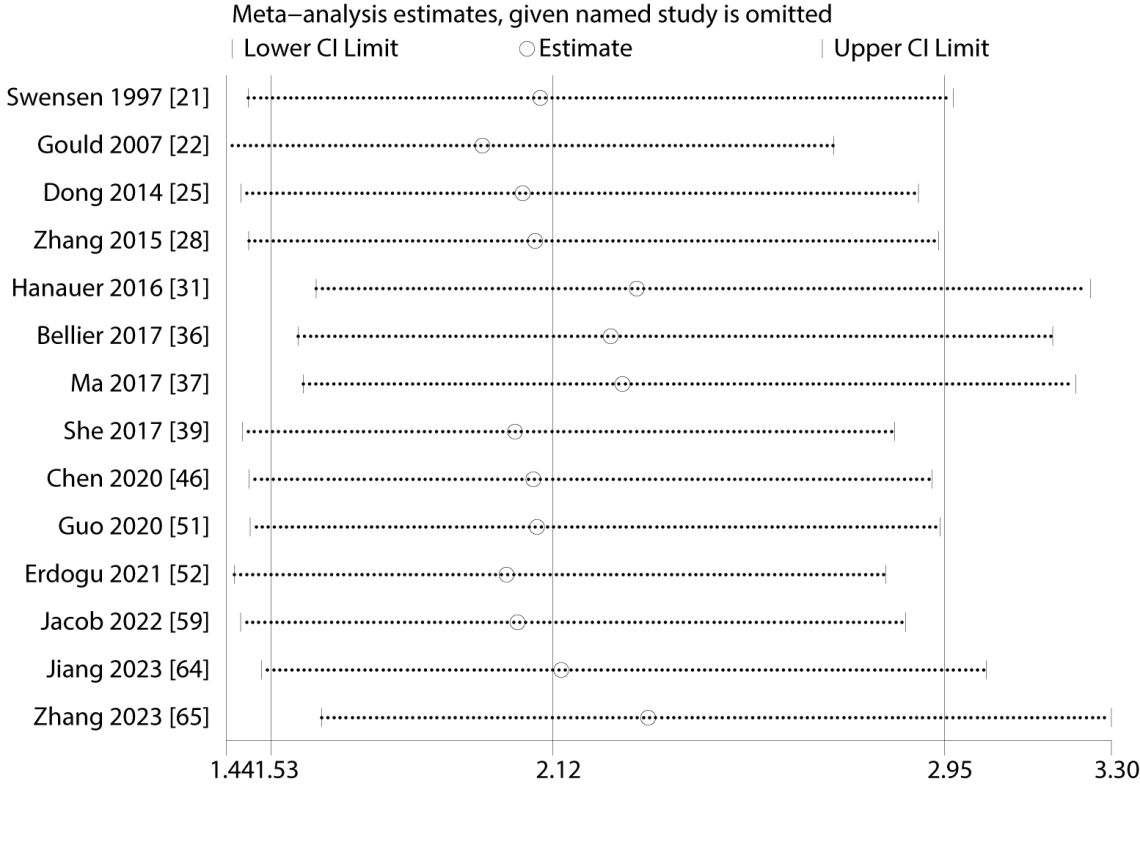


Figure S24. Sensitivity analysis for the association of cigarette smoker with the risk of malignancy in patients with incidental SPNs


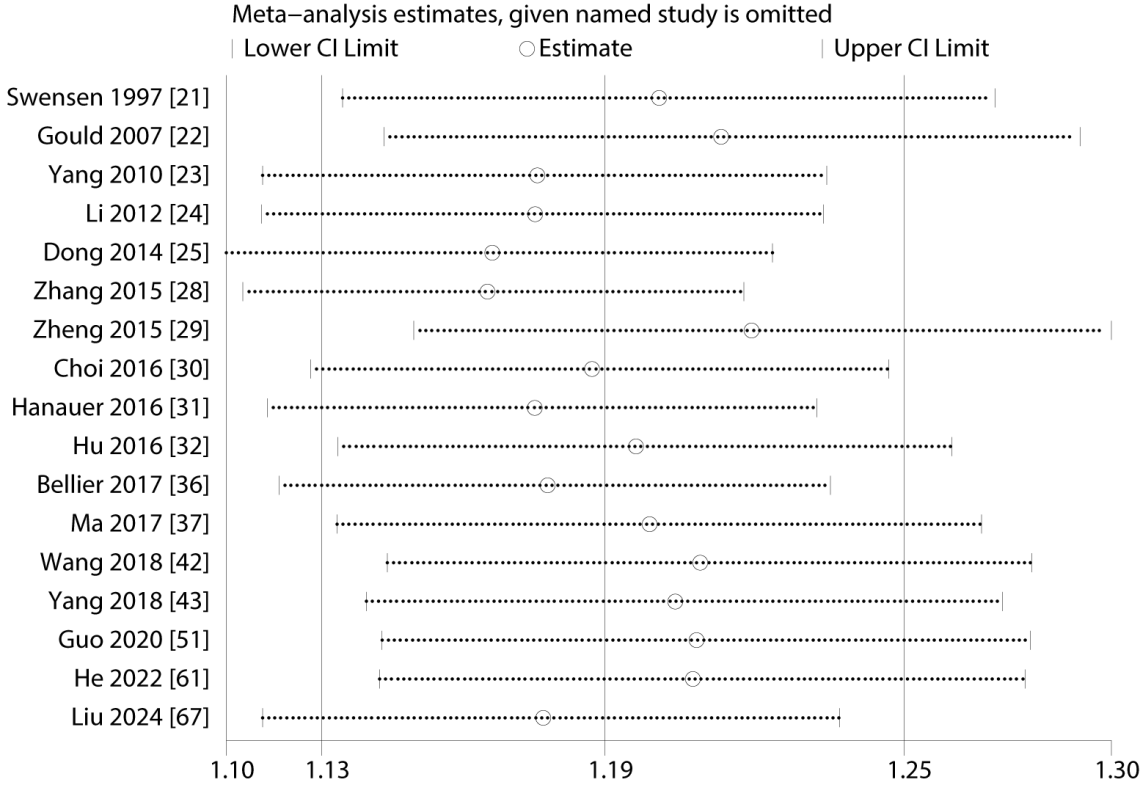


Figure S25. Sensitivity analysis for the association of diameter of nodules with the risk of malignancy in patients with incidental SPNs


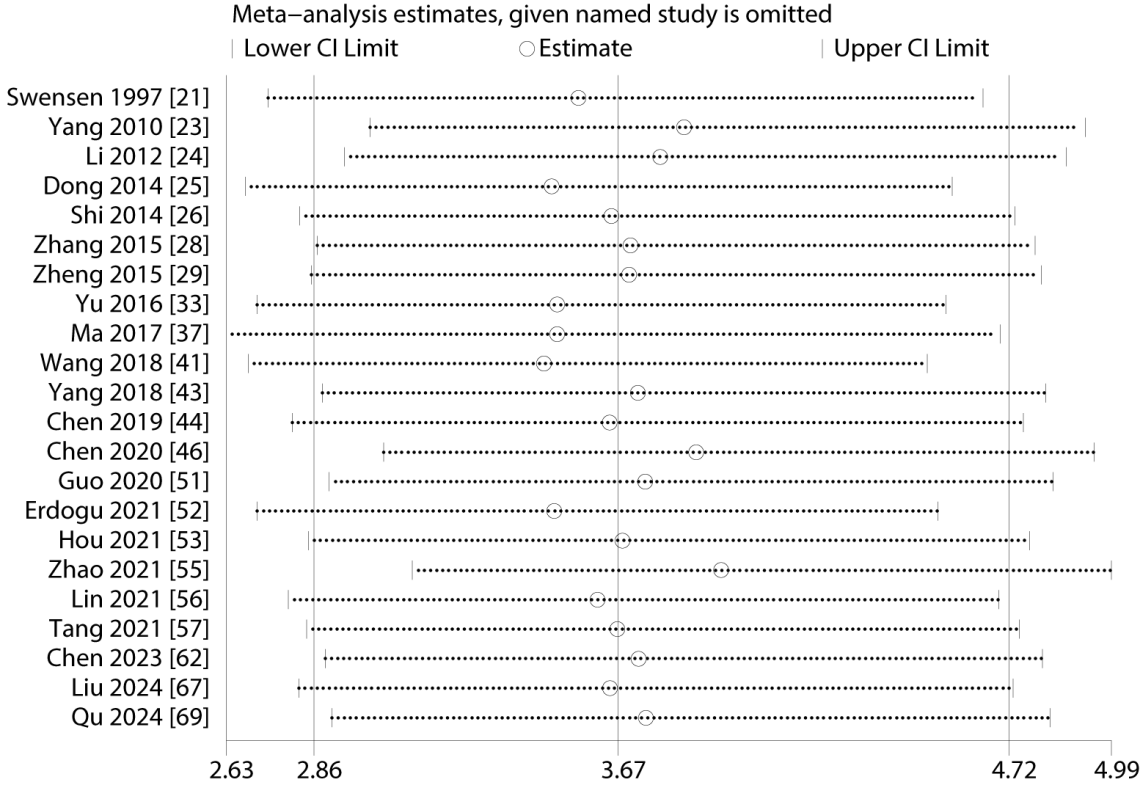


Figure S26. Sensitivity analysis for the association of spiculation with the risk of malignancy in patients with incidental SPNs


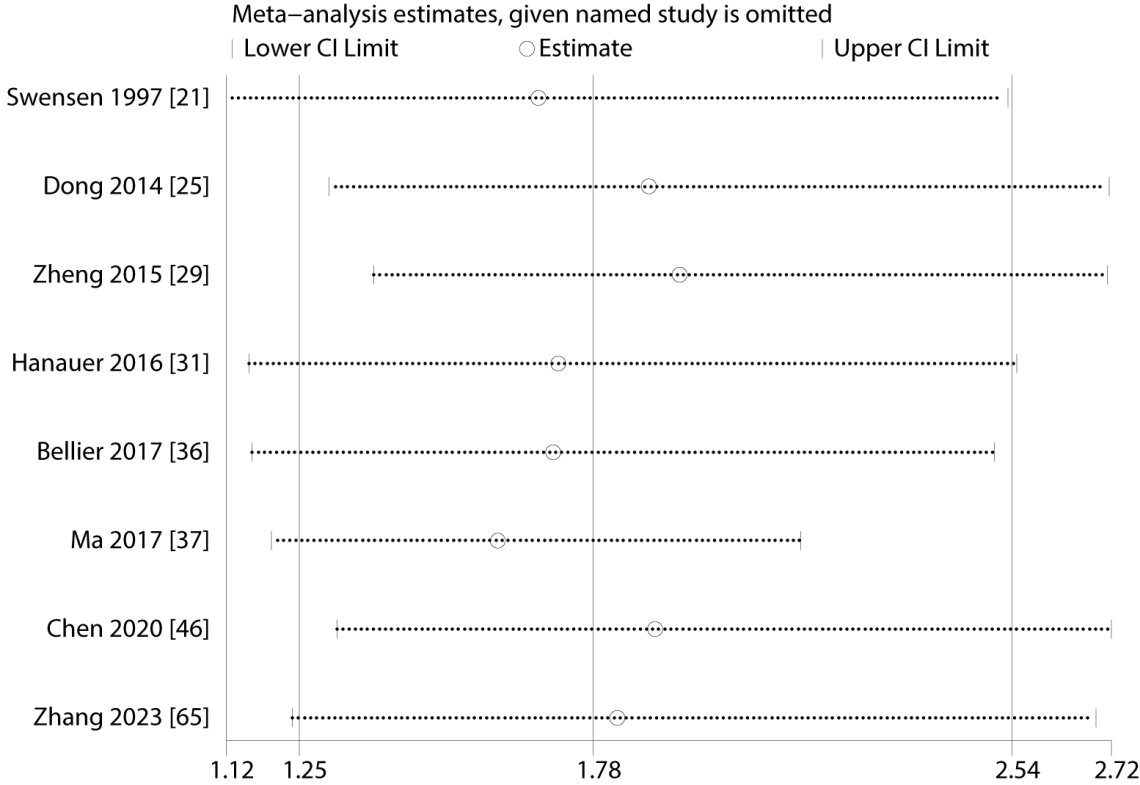


Figure S27. Sensitivity analysis for the association of upper lobe with the risk of malignancy in patients with incidental SPNs


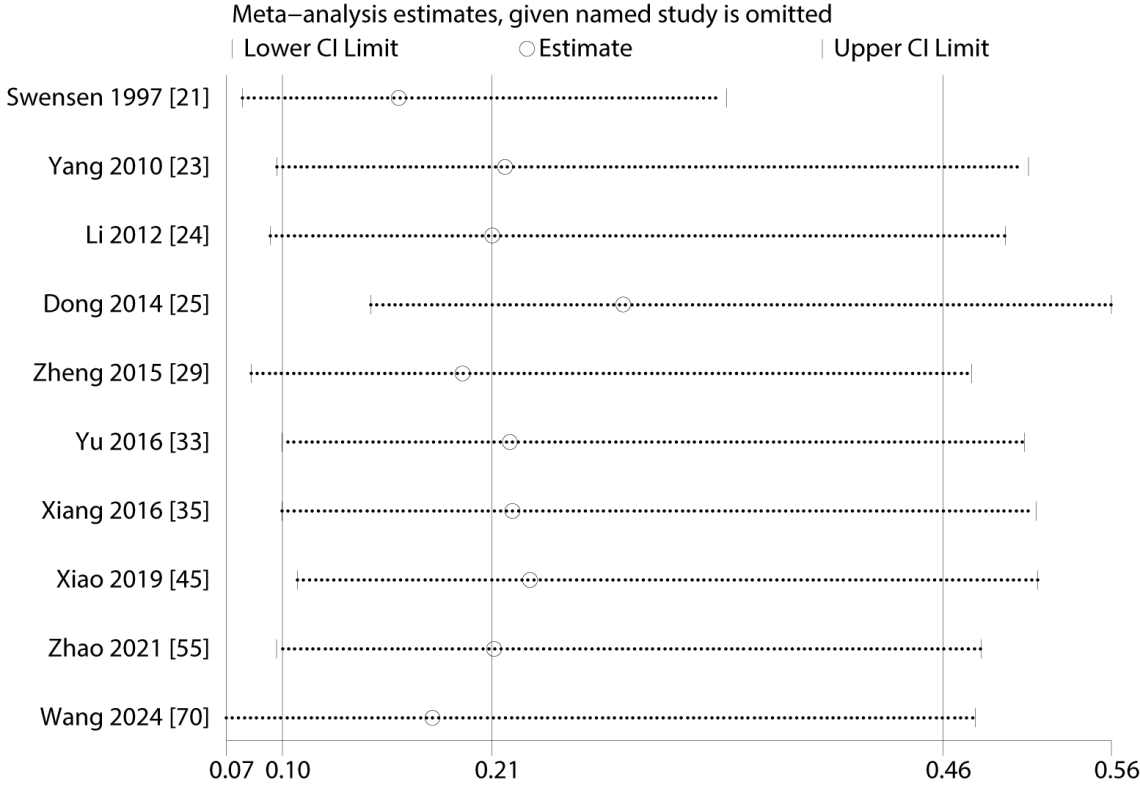


Figure S28. Sensitivity analysis for the association of calcification with the risk of malignancy in patients with incidental SPNs


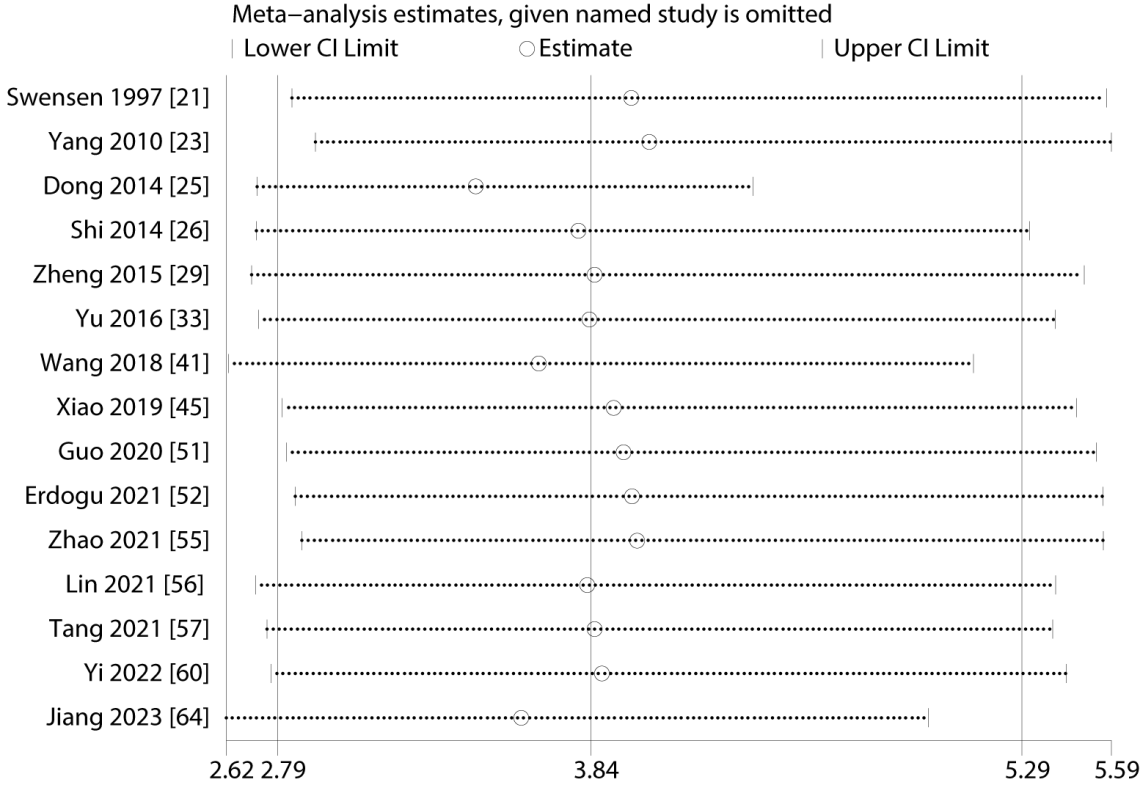


Figure S29. Sensitivity analysis for the association of lobulation with the risk of malignancy in patients with incidental SPNs


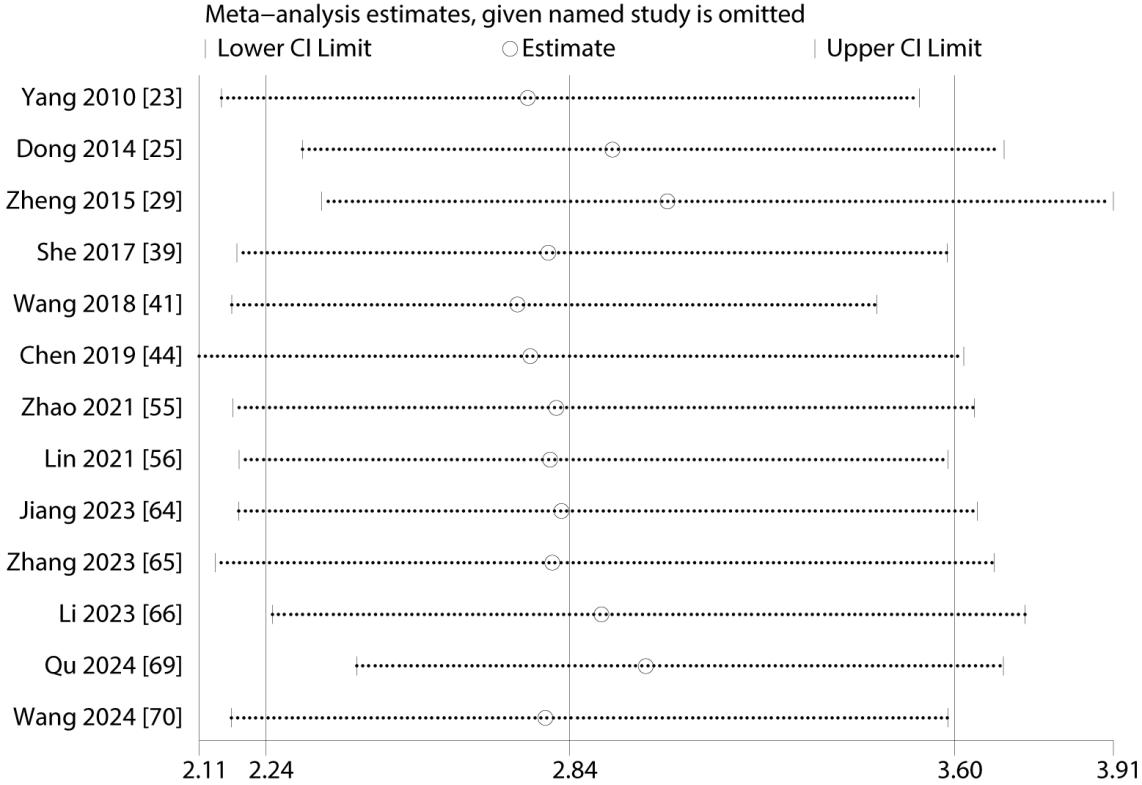


Figure S30. Sensitivity analysis for the association of pleural indentation with the risk of malignancy in patients with incidental SPNs


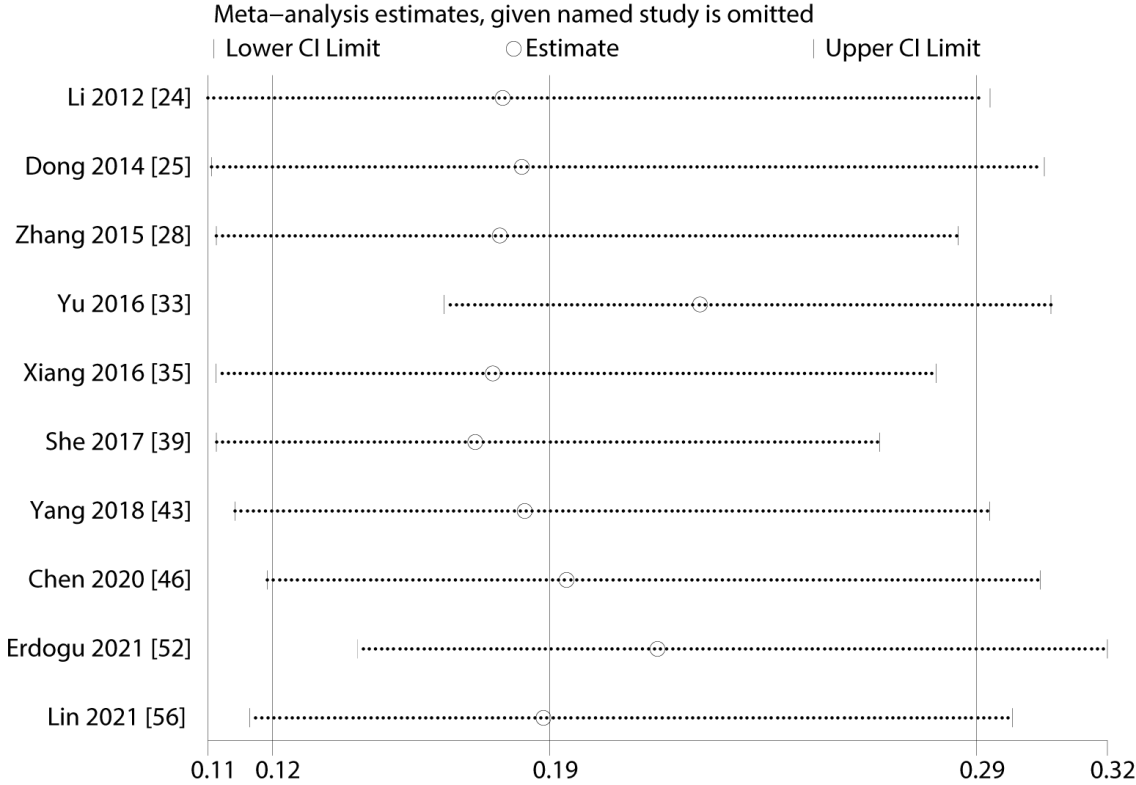


Figure S31. Sensitivity analysis for the association of clear border with the risk of malignancy in patients with incidental SPNs


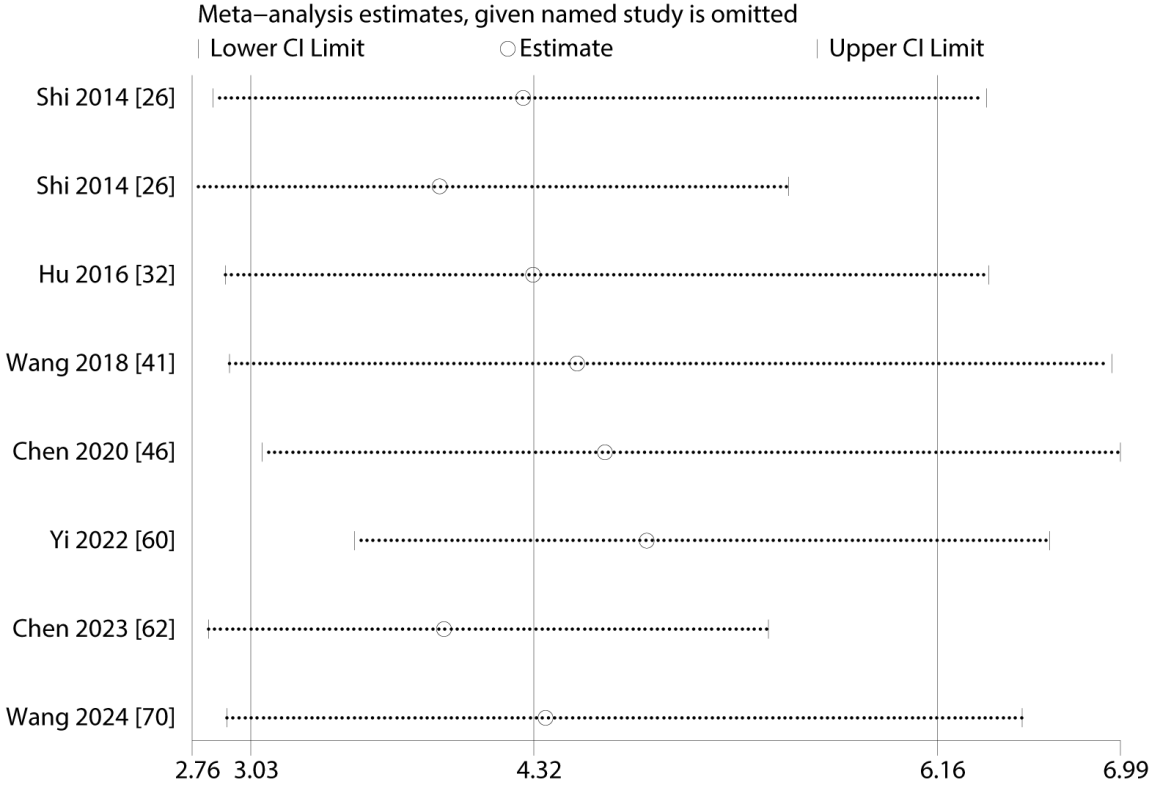


Figure S32. Sensitivity analysis for the association of vascular convergence with the risk of malignancy in patients with incidental SPNs


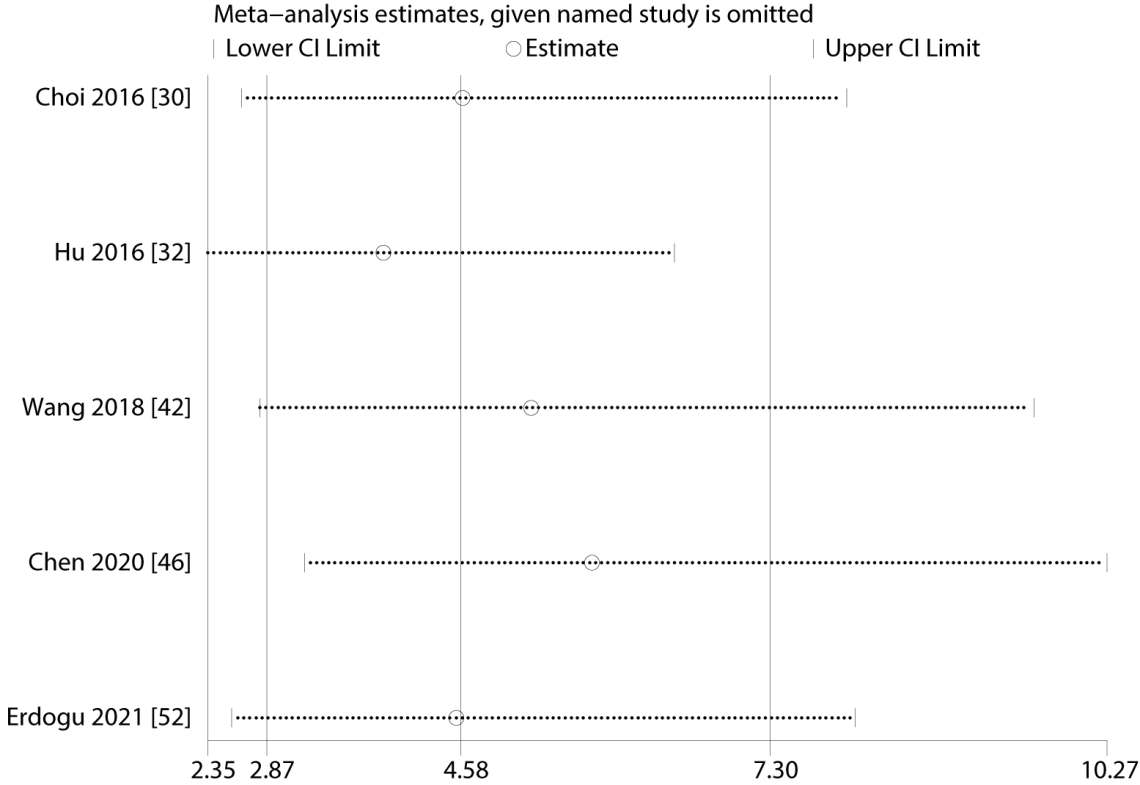


Figure S33. Sensitivity analysis for the association of solid nodules with the risk of malignancy in patients with incidental SPNs


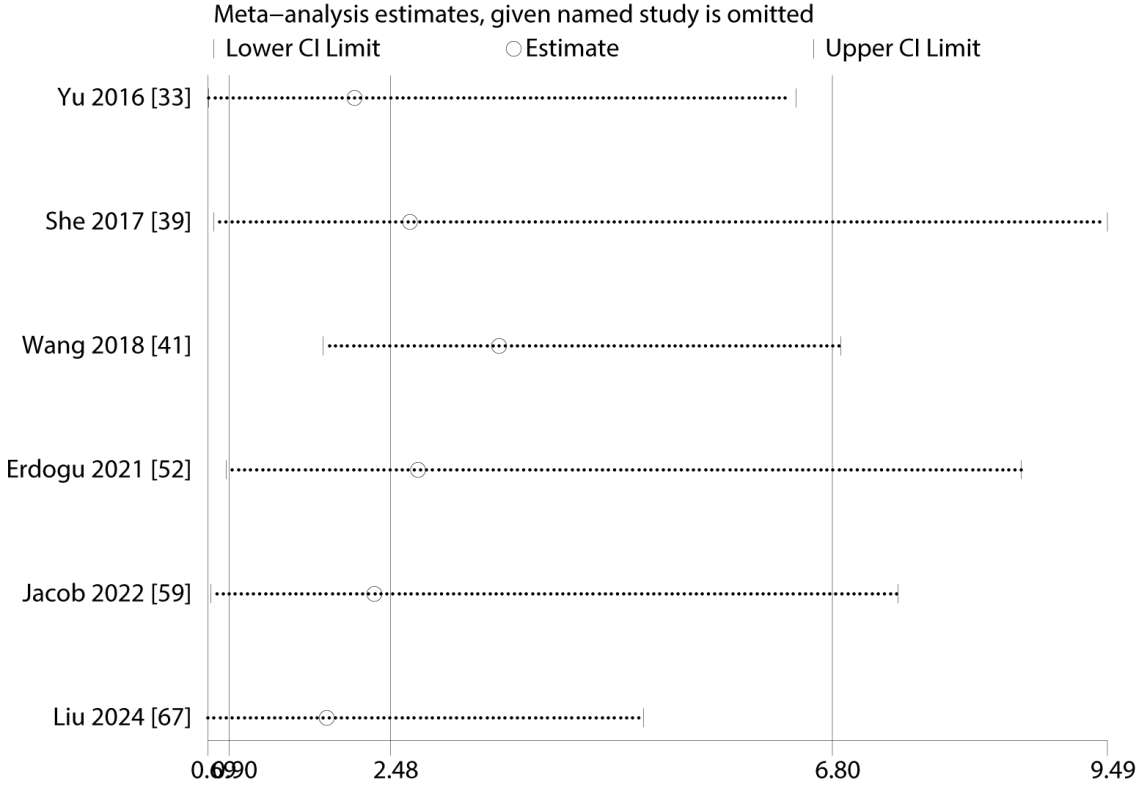


Figure S34. Sensitivity analysis for the association of air bronchogram with the risk of malignancy in patients with incidental SPNs


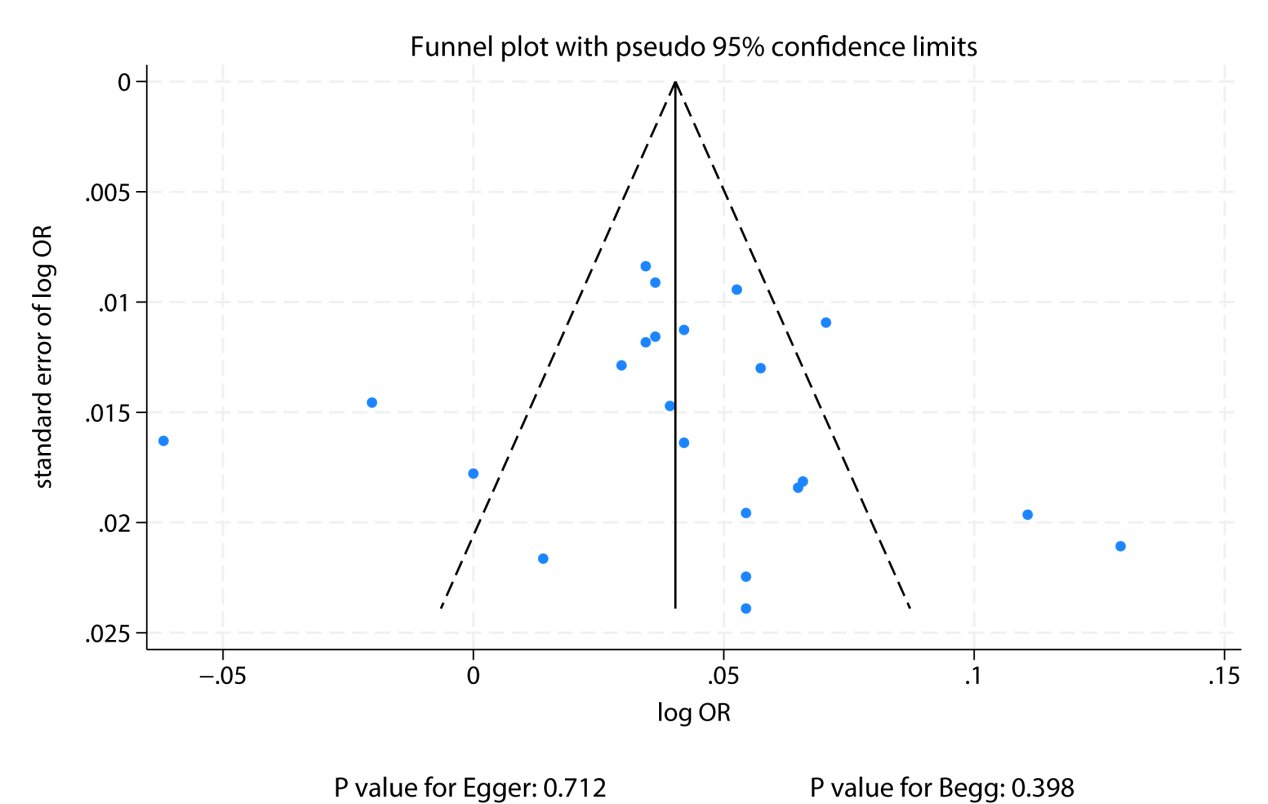


Figure S35. Funnel plot for the association of age with the risk of malignancy in patients with incidental SPNs


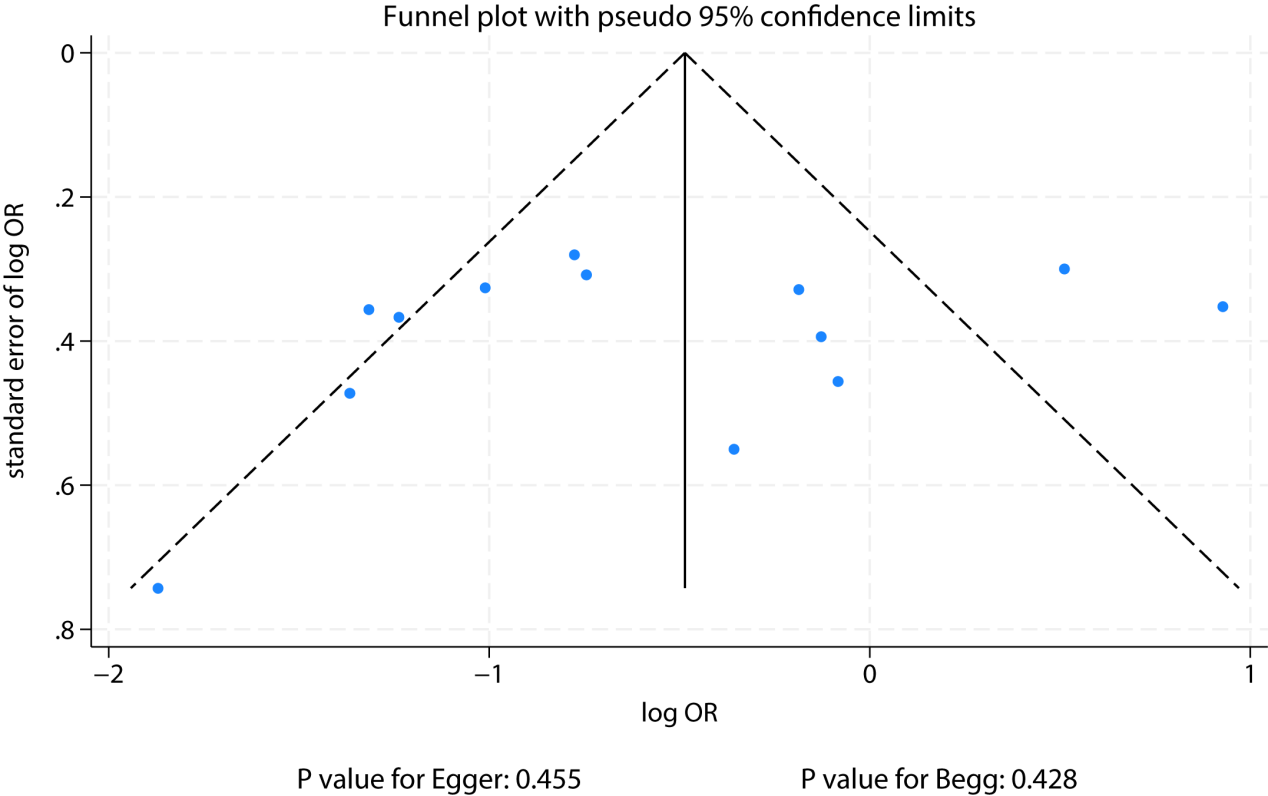


Figure S36. Funnel plot for the association of male vs female with the risk of malignancy in patients with incidental SPNs


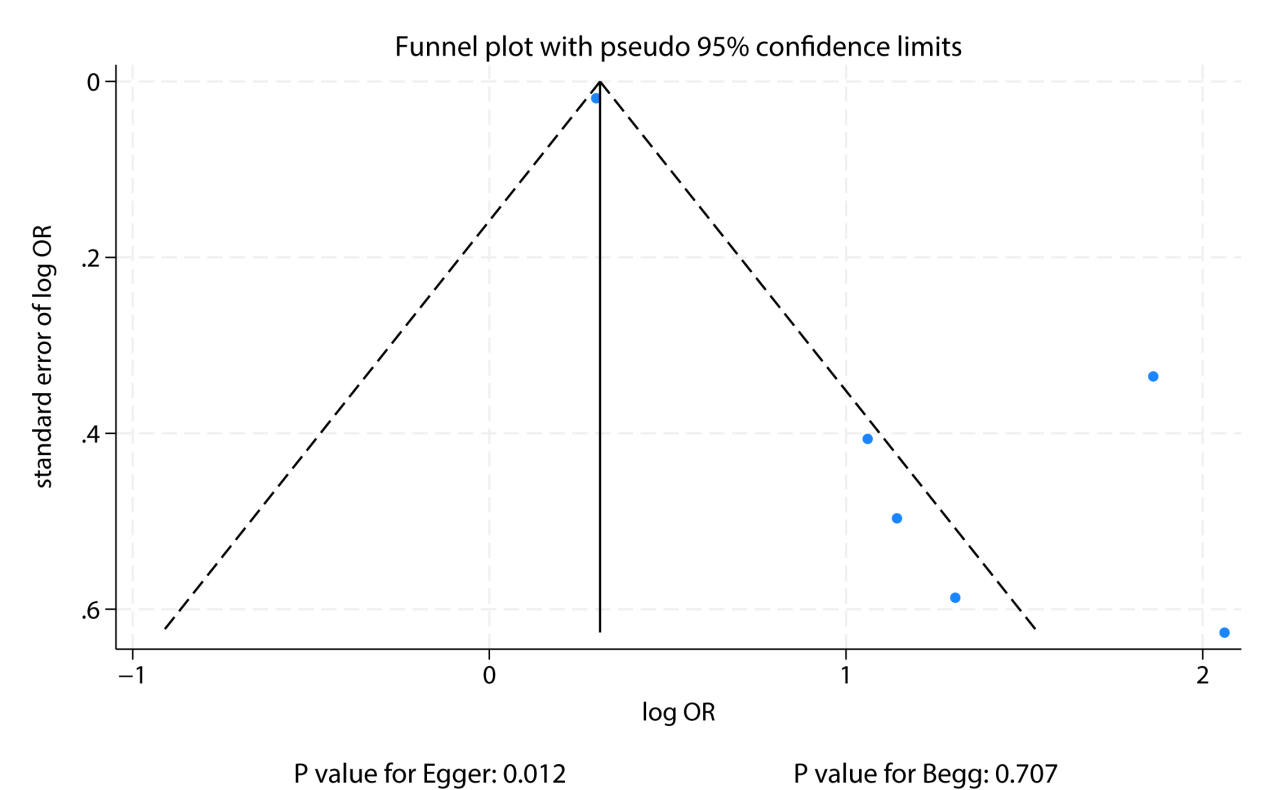


Figure S37. Funnel plot for the association of cancer history with the risk of malignancy in patients with incidental SPNs


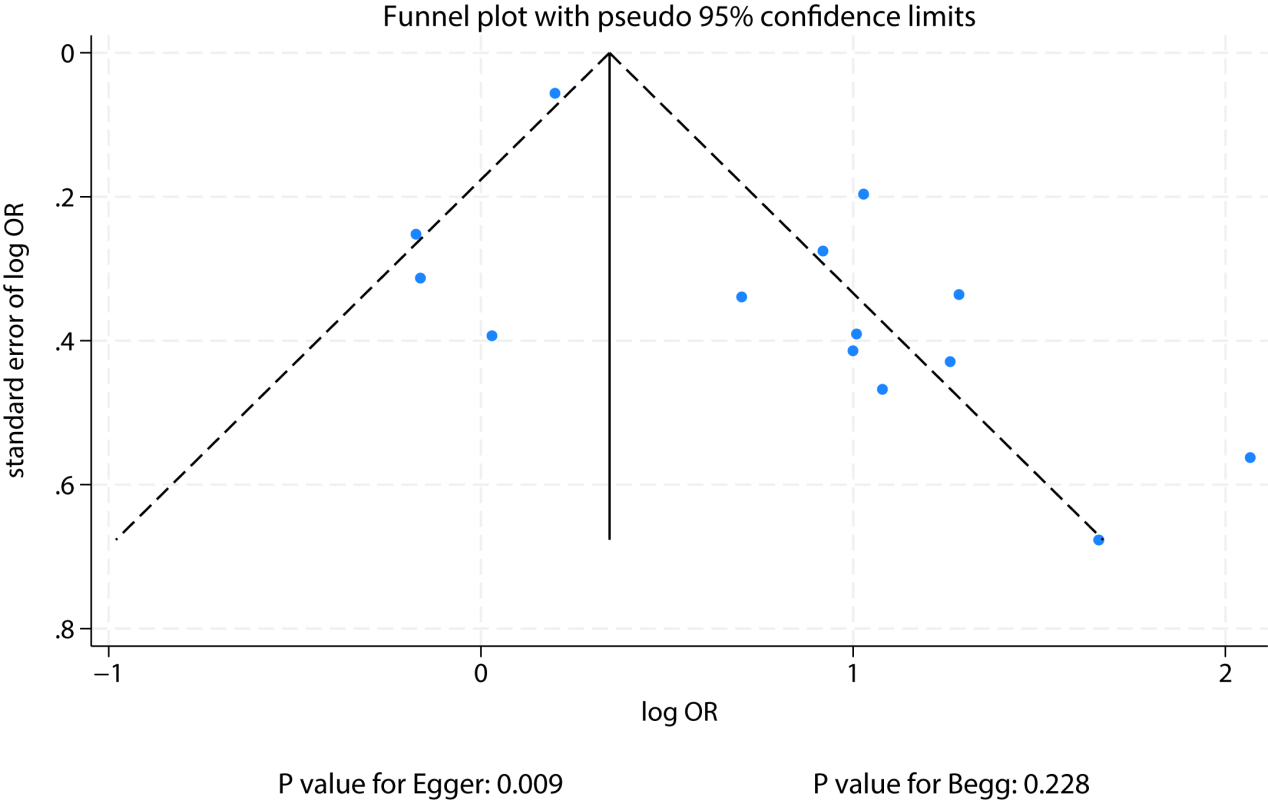


Figure S38. Funnel plot for the association of cigarette smoker with the risk of malignancy in patients with incidental SPNs


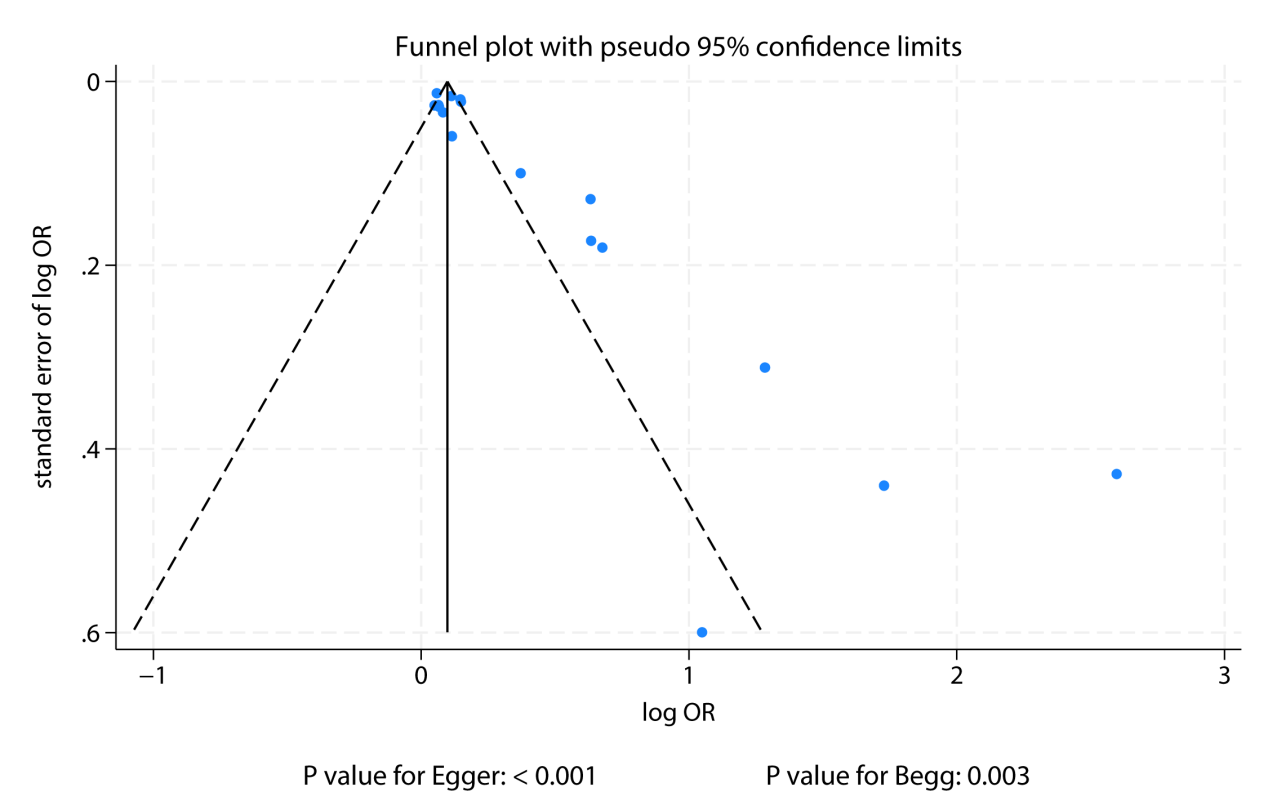


Figure S39. Funnel plot for the association of diameter of nodules with the risk of malignancy in patients with incidental SPNs


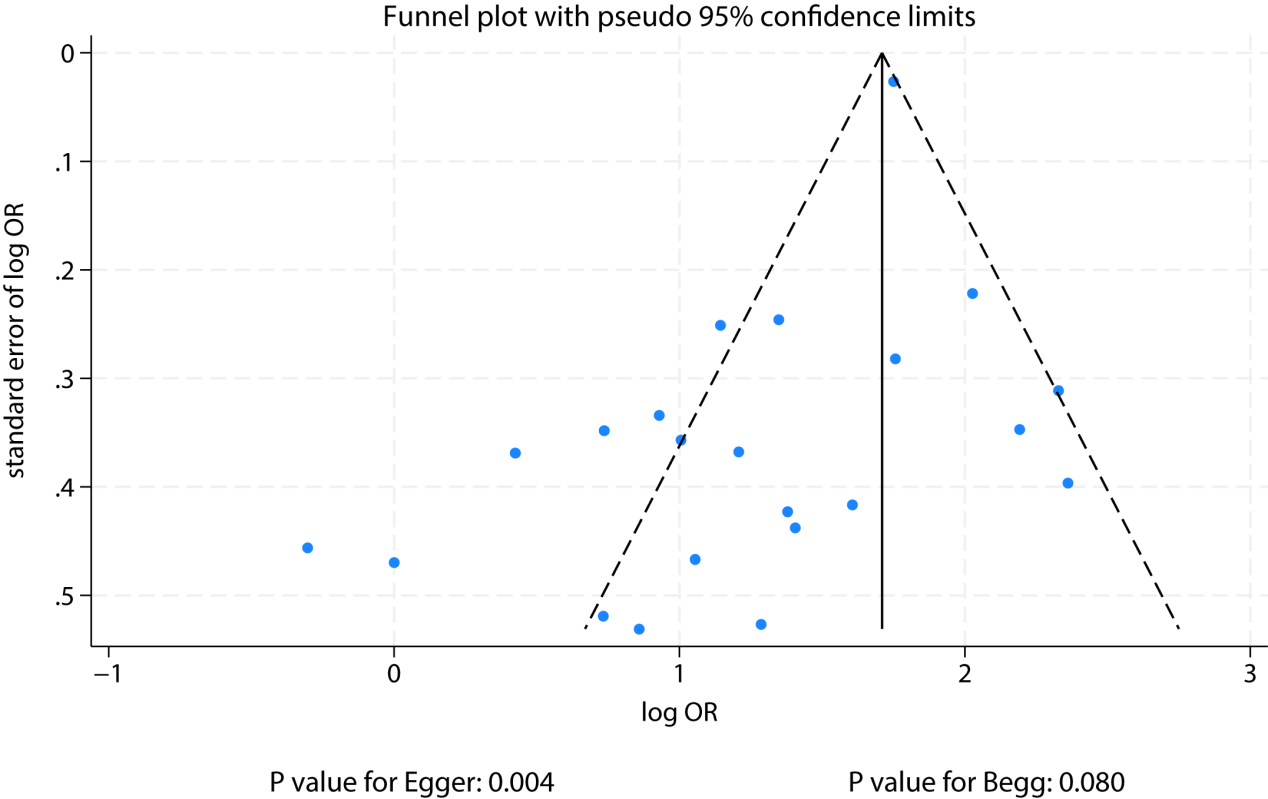


Figure S40. Funnel plot for the association of spiculation with the risk of malignancy in patients with incidental SPNs


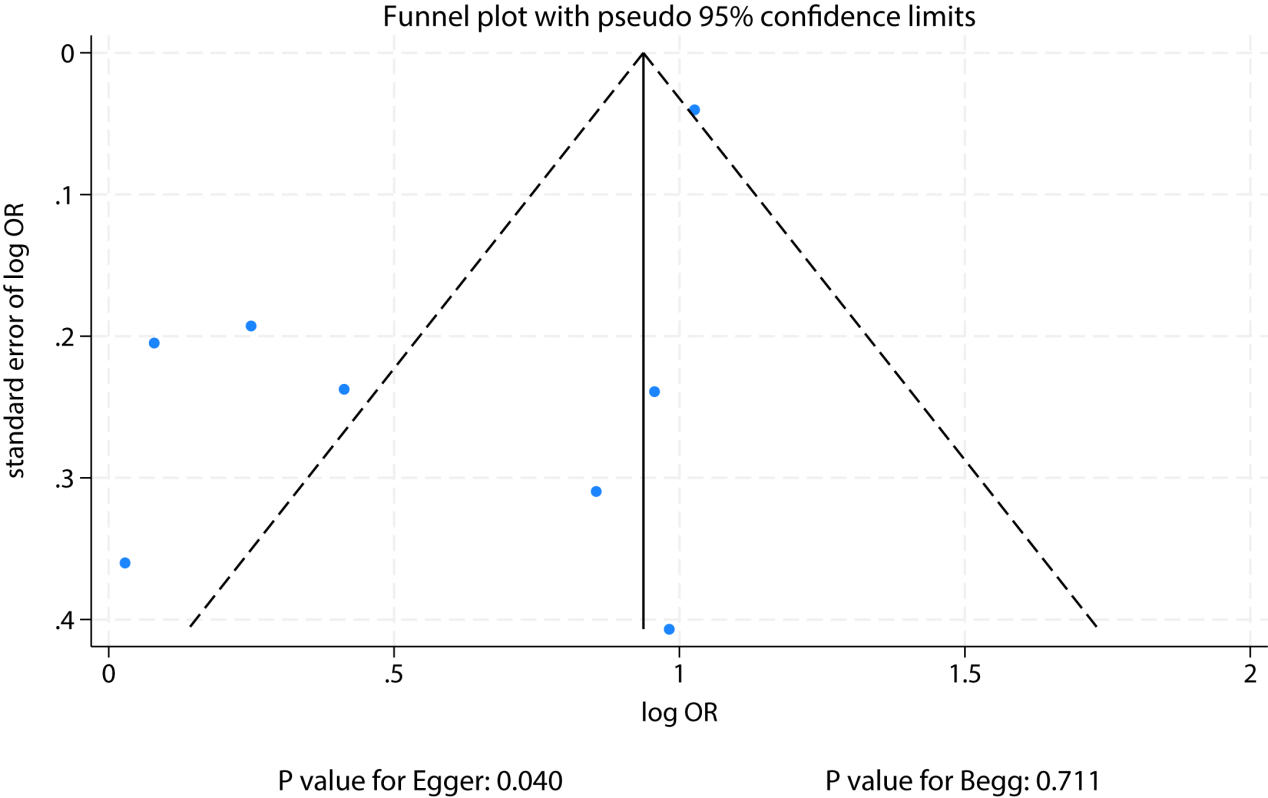


Figure S41. Funnel plot for the association of upper lobe with the risk of malignancy in patients with incidental SPNs


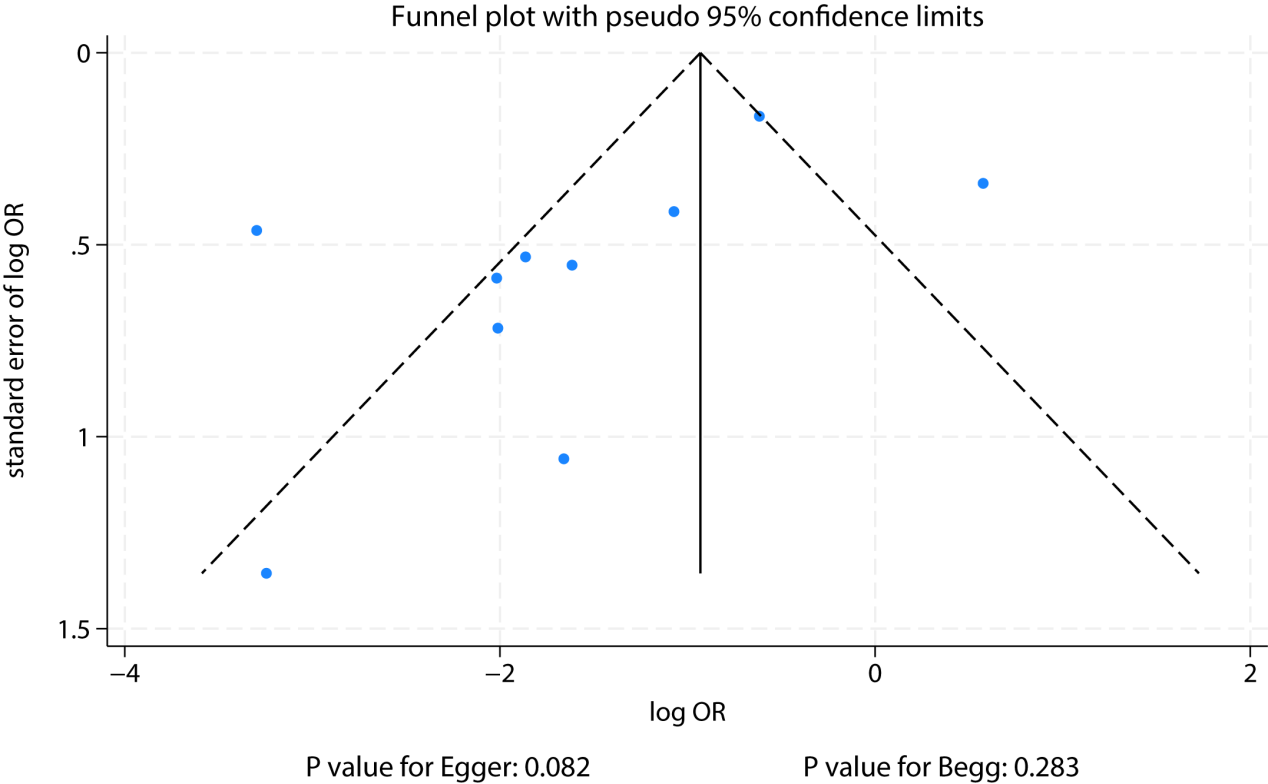


Figure S42. Funnel plot for the association of calcification with the risk of malignancy in patients with incidental SPNs


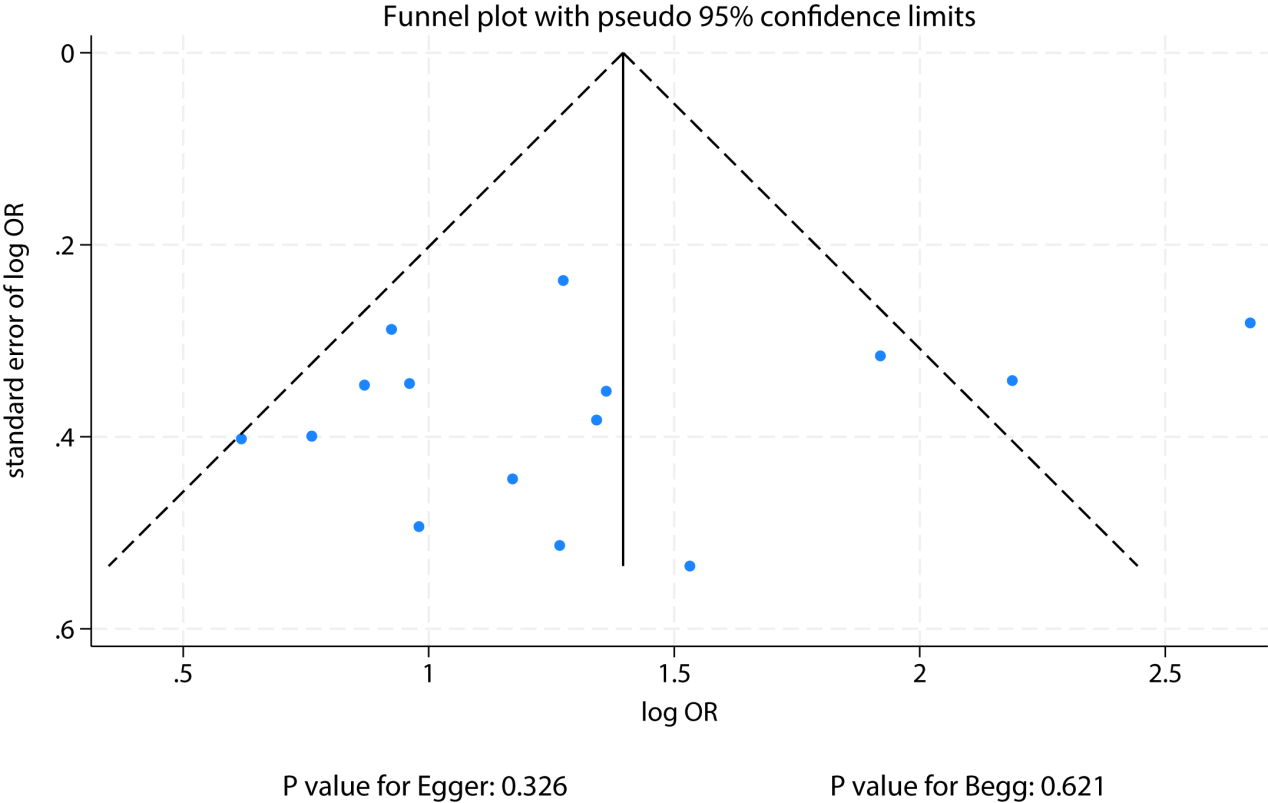


Figure S43. Funnel plot for the association of lobulation with the risk of malignancy in patients with incidental SPNs


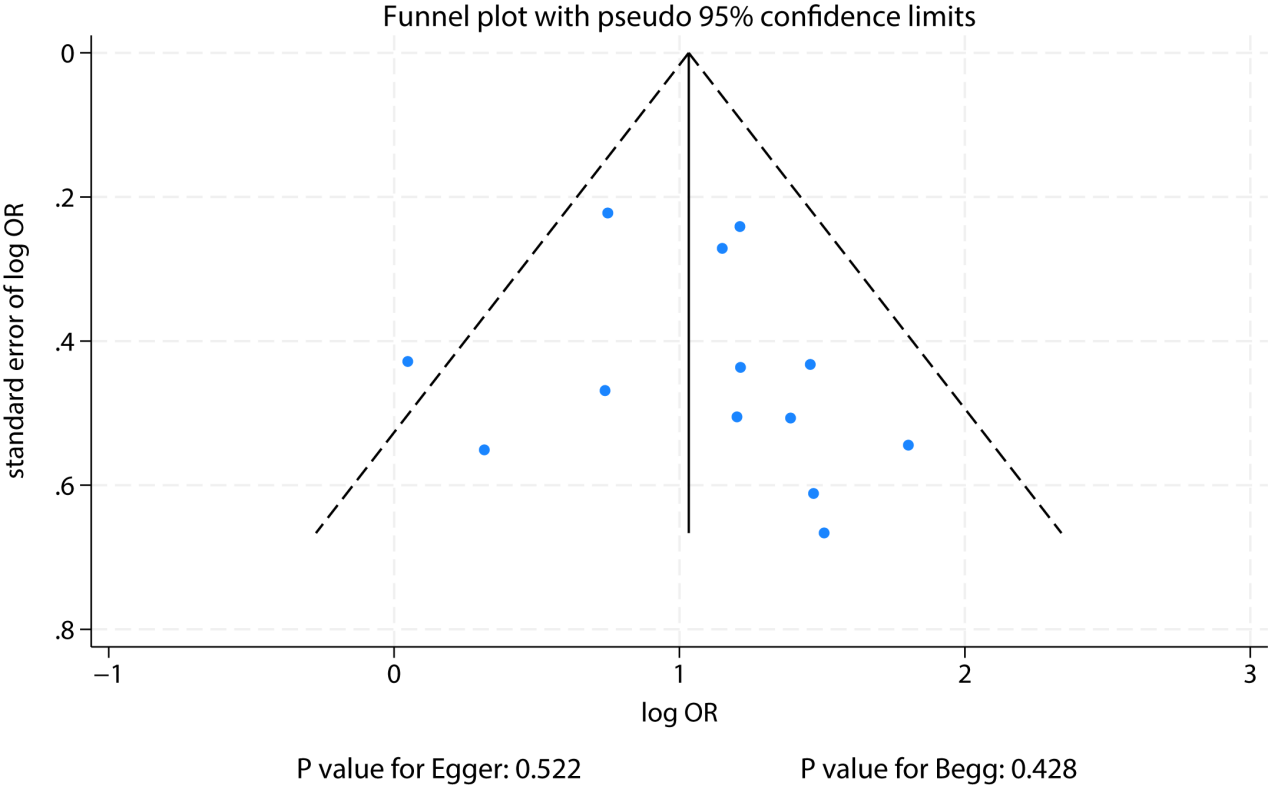


Figure S44. Funnel plot for the association of pleural indentation with the risk of malignancy in patients with incidental SPNs


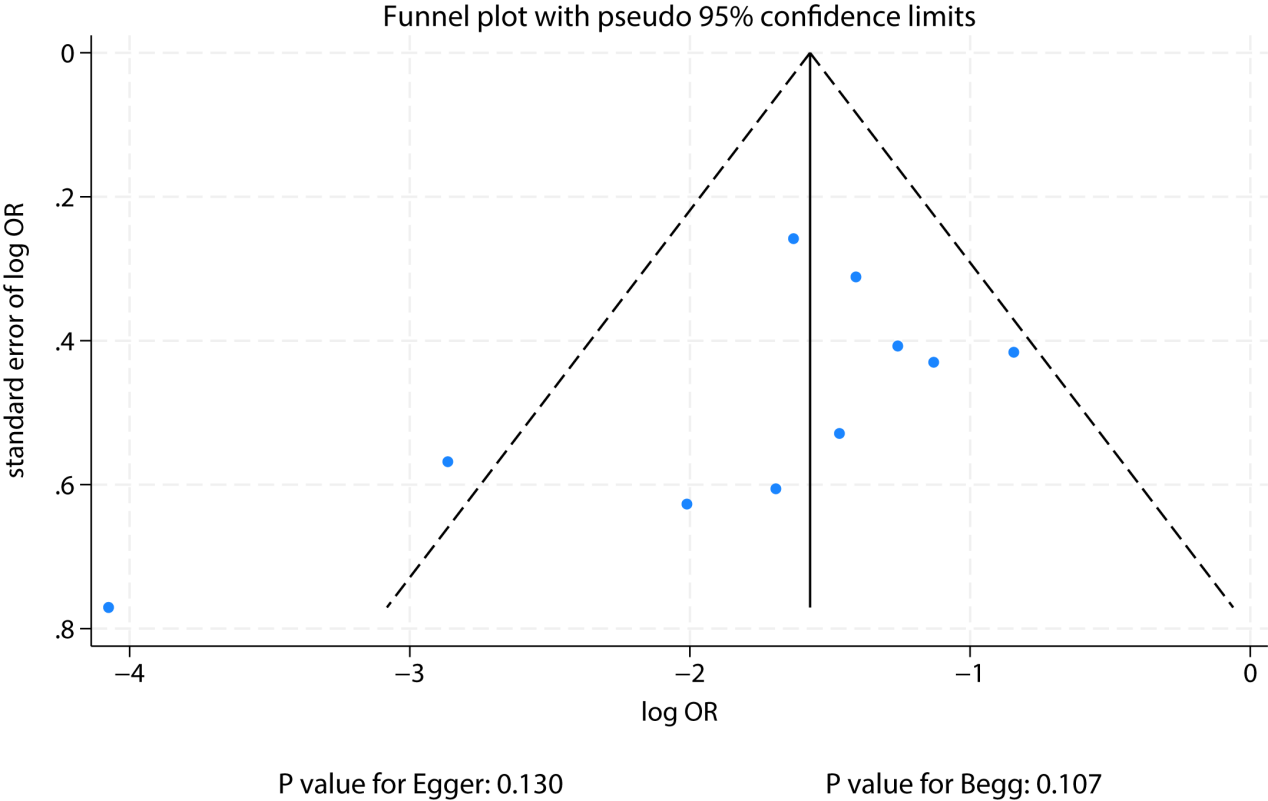


Figure S45. Funnel plot for the association of clear border with the risk of malignancy in patients with incidental SPNs


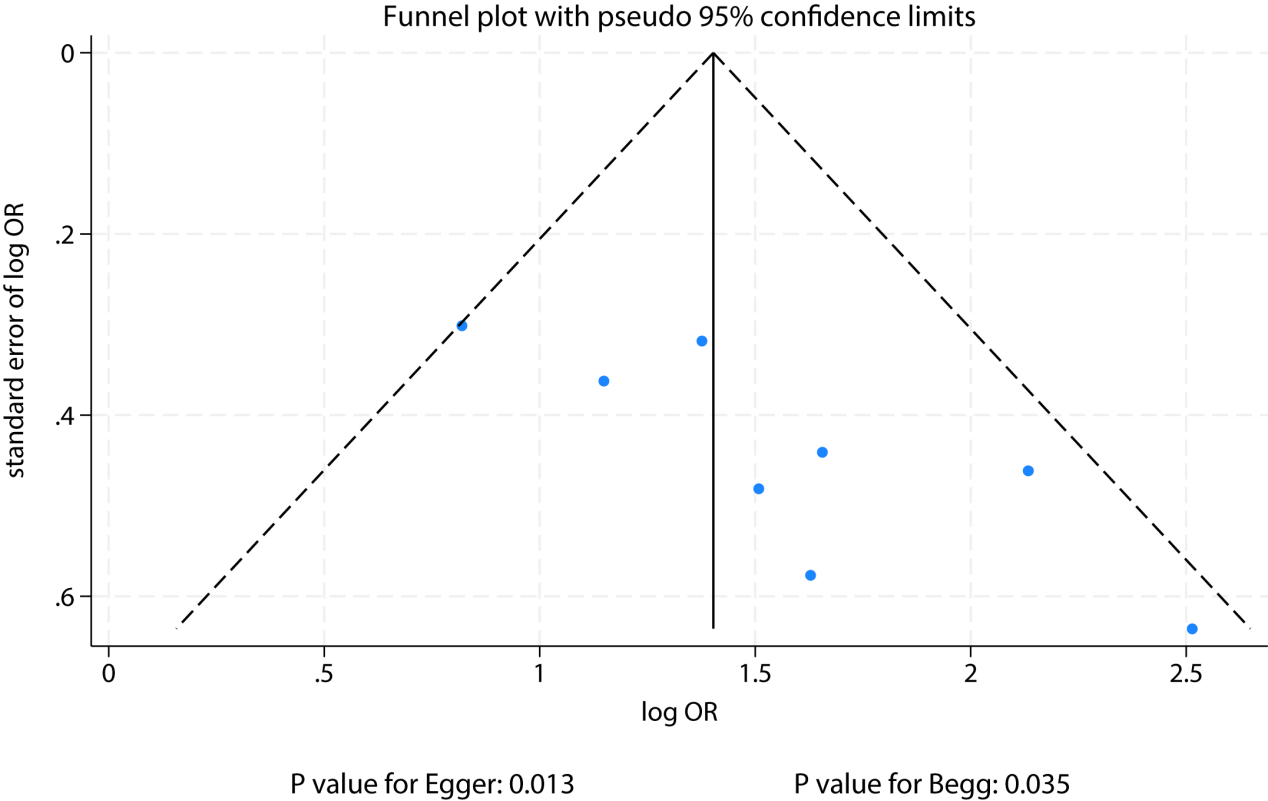


Figure S46. Funnel plot for the association of vascular convergence with the risk of malignancy in patients with incidental SPNs


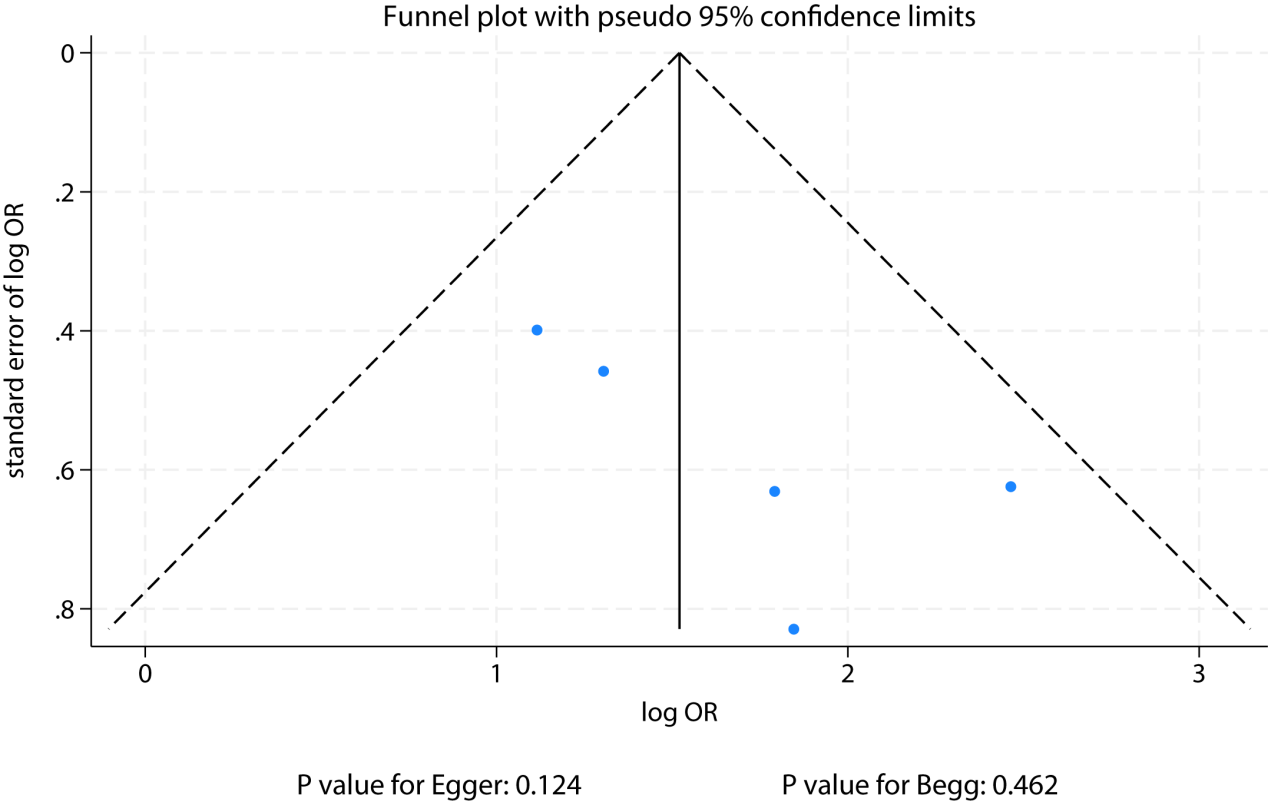


Figure S47. Funnel plot for the association of solid nodules with the risk of malignancy in patients with incidental SPNs


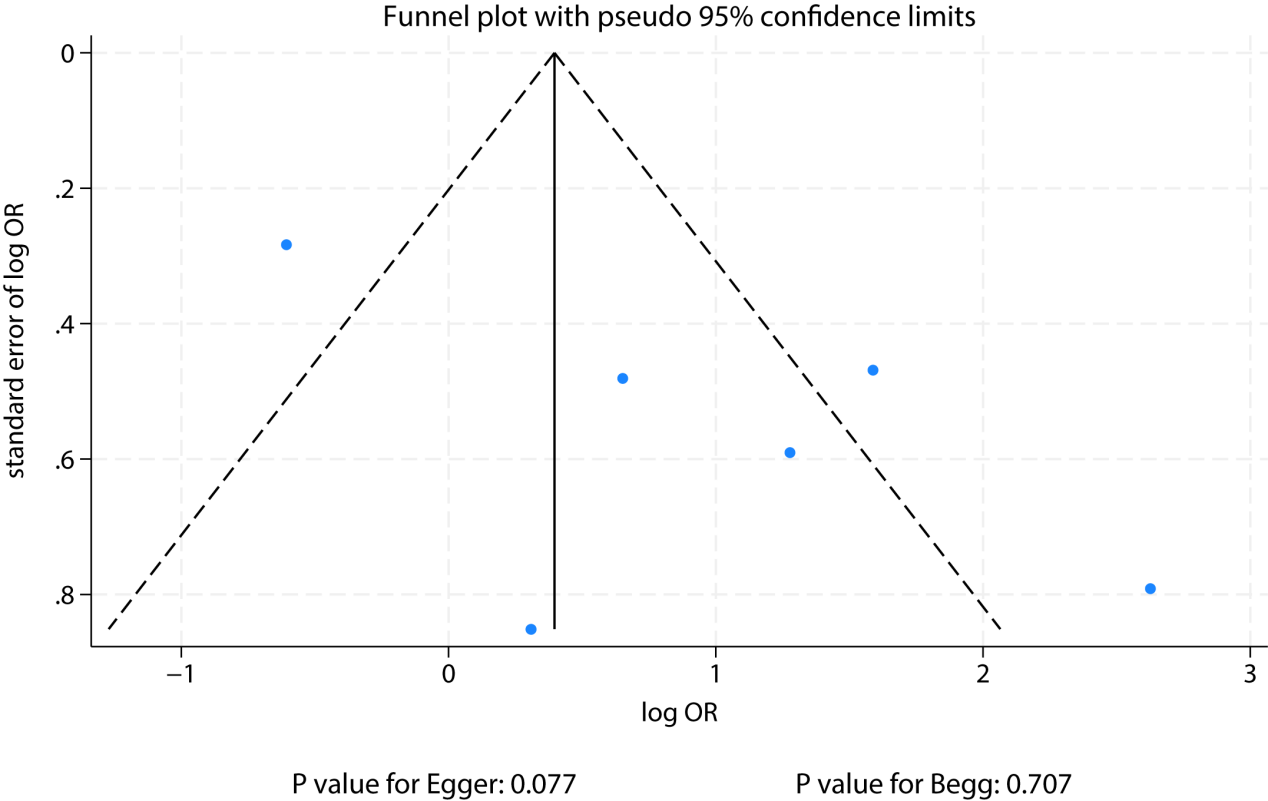


Figure S48. Funnel plot for the association of air bronchogram with the risk of malignancy in patients with incidental SPNs
